# Strategy and Processing Speed Eclipse Individual Differences in Control Ability in Conflict Tasks

**DOI:** 10.1037/xlm0001028

**Published:** 2021-09-30

**Authors:** Craig Hedge, Georgina Powell, Aline Bompas, Petroc Sumner

**Affiliations:** 1 School of Psychology, Cardiff University

**Keywords:** attention control, diffusion model for conflict tasks, individual differences, inhibition, response control

## Abstract

Response control or inhibition is one of the cornerstones of modern cognitive psychology, featuring prominently in theories of executive functioning and impulsive behavior. However, repeated failures to observe correlations between commonly applied tasks have led some theorists to question whether common response conflict processes even exist. A challenge to answering this question is that behavior is multifaceted, with both conflict and nonconflict processes (e.g., strategy, processing speed) contributing to individual differences. Here, we use a cognitive model to dissociate these processes; the diffusion model for conflict tasks (Ulrich et al., 2015). In a meta-analysis of fits to seven empirical datasets containing combinations of the flanker, Simon, color-word Stroop, and spatial Stroop tasks, we observed weak (*r* < .05) zero-order correlations between tasks in parameters reflecting conflict processing, seemingly challenging a general control construct. However, our meta-analysis showed consistent positive correlations in parameters representing processing speed and strategy. We then use model simulations to evaluate whether correlations in behavioral costs are diagnostic of the presence or absence of common mechanisms of conflict processing. We use the model to impose known correlations for conflict mechanisms across tasks, and we compare the simulated behavior to simulations when there is no conflict correlation across tasks. We find that correlations in strategy and processing speed can produce behavioral correlations equal to, or larger than, those produced by correlated conflict mechanisms. We conclude that correlations between conflict tasks are only weakly informative about common conflict mechanisms if researchers do not control for strategy and processing speed.

Controlling our responses in the presence of conflicting information is a core facet of executive function (Miyake et al., 2000). Response control (sometimes called response inhibition or attentional control) is typically measured in commonly used paradigms such as the Stroop (Stroop, 1935), the Eriksen flanker (Eriksen & Eriksen, 1974), Simon (Simon & Rudell, 1967), and the antisaccade (Hallett, 1978) and stop-signal (Logan, 1994) tasks. Individual differences in response control have been linked to several neuropsychological disorders, including substance abuse, attention deficit hyperactivity disorder (ADHD), schizophrenia, and Parkinson’s disease (Chambers et al., 2009; Gauggel et al., 2004; Lansbergen et al., 2007; Moeller et al., 2002; Verdejo-Garcia et al., 2007). Therefore, understanding the source(s) of variation in response control is key to understanding cognition in both healthy and clinical populations.

In both theoretical and applied work, it is common to assume either a common underlying response control trait, or some degree of overlap in response control mechanisms underlying different tasks (for reviews, see Bari & Robbins, 2013; von Bastian et al., 2020). However, the assumption of common mechanisms has received inconsistent support from correlational studies, with performance in different control tasks showing inconsistent or absent correlations with each other (Aichert et al., 2012; Friedman & Miyake, 2004; Hamilton et al., 2015; Hedge et al., 2018b; Ivanov et al., 2011; Stahl et al., 2014; Wager et al., 2005). This has led some theorists to question the value of inhibition as a psychometric construct (Rey-Mermet et al., 2018), which has serious implications for both theoretical work and for the applications of the construct to clinical domains.

Evaluating whether a common and useful inhibition construct exists is obstructed by a key challenge: the way performance is typically measured may be suboptimal for examining individual differences even if the trait does exist (Draheim et al., 2016; Hedge et al., 2018b; Rouder & Haaf, 2019). There is a habit in psychology to use performance in key tasks as proxies for underlying mechanisms, such as memory, attention or control (cf. Verbruggen et al., 2014). But the ingredients to performance are multifaceted, and individual variation does not necessarily come from the same source as the well-studied within-subject effects (Boy & Sumner, 2014). For example, although the main cause of the Stroop effect is conflict, individual differences in the *size* of the Stroop effect could come from differences in strategy, language processing or even visual acuity (e.g., not wearing your glasses), rather than ability to control conflict.

## Strategy and General Processing Speed Contaminate Measures of Inhibitory Ability

We recently conducted a meta-analysis that illustrated the problem of measuring individual differences in inhibitory ability, which are normally captured through congruency effects, because it is generally assumed that subtracting conditions to produce a cost removes speed-accuracy strategy effects. However, some tasks use reaction time (RT) costs and some use error costs and across a wide range of tasks, RT costs and error costs taken from *the same task* show little correlation (*r* = .17; Hedge et al., 2018). In other words, if we were to rank individuals from best to worse in inhibitory ability based on their Stroop cost in RTs, we would come to a very different ordering than if we used the Stroop cost in errors.

To some extent, low correlations between RT costs and error costs are to be expected because subtractions lower reliability, which attenuates correlations (Enkavi et al., 2019; Hedge et al., 2018b; Miller & Ulrich, 2013; Paap & Sawi, 2016). However, this does not fully account for the low and inconsistent pattern, with significant negative correlations sometimes observed between the two purported measures of the same ability. We explain this in the framework of evidence accumulation models (for example, Brown & Heathcote, 2008; Ratcliff, 1978). We assume that individuals differ in at least two dimensions. The first is their *ability* to select the correct response based on the information. Individuals who are ‘better’ at inhibiting conflicting information should show both smaller RT costs and error costs, leading to a positive correlation. The second is their *strategy,* reflecting how much information they wait for before they make a decision. Individuals who are more cautious produce larger RT costs and smaller error costs, leading to negative correlations. Critically, the traditional approach of subtracting conditions does not remove strategy effects, which can mask individual differences in inhibitory ability (Hedge et al., 2018).

In addition to strategy differences, general processing speed can also confound the measurement of response control (Miller & Ulrich, 2013). Using a psychometric model of mean RTs, Miller & Ulrich show that correlation between behaviorally measured RT costs taken from two tasks can be weak despite there being strong underlying correlation in the ability of interest (for example, inhibition). This is because factors such as general speed can be expected to contaminate measured RT costs. Reanalysis of several factor analytic studies observed that individual differences in conflict tasks can be accounted for by a general processing speed factor, without need for a separate inhibition factor (Jewsbury et al., 2016; see also Friedman & Miyake, 2017; Karr et al., 2018; Rey-Mermet, Gade, Souza, et al., 2019). In an evidence accumulation framework, greater efficiency in general information processing produces smaller RT costs and errors costs, thus manifesting in the same way as greater inhibitory ability (Hedge et al., 2018a).

Taken together, the literature paints a challenging picture for assessing whether common mechanisms of inhibition or conflict processing exist. The size of an individual’s RT and/or error cost in a given task reflects some unknown combination of their ability to overcome conflict, their strategy, and other processing abilities. The relative contribution of these processes to behavior will differ between tasks, or between different implementations of a given task (Hedge et al., 2018; Unsworth et al., 2004). To reframe the question, if common mechanisms of inhibition or conflict processing did exist, would we know?

To address this question, we take a cognitive modeling approach to separate out and quantify conflict, strategy and general speed parameters, examine where (if at all) they correlate between tasks when we fit empirical data, and evaluate how each parameter manifests in observable behavior by using simulations.

## Overview of the Study

Our main aim in the first part of this article is to apply a cognitive model (the diffusion model for conflict tasks [DMC]; Ulrich et al., 2015) to multiple empirical datasets to decompose behavior into constituent processes. This allows us to examine correlations in parameters that represent conflict mechanisms separately from parameters that do not directly represent conflict mechanisms. We focus on datasets containing the flanker, Simon, Stroop, and spatial Stroop tasks, and adopt a meta-analytic approach to maximize power and integrate across datasets. To preempt the main findings, we observe no correlation in the model parameters representing conflict processes. We do observe consistent correlations in model parameters representing nonconflict processes (for example, strategy, general processing speed), providing converging evidence for previous claims (for example, Jewsbury et al., 2016).

In the final part of the article, we use the model to simulate data from known theoretical positions to ask whether observable performance would diagnose the difference between the presence or absence of common conflict processing. Here, we use the DMC to generate data for two hypothetical tasks with a known correlation in parameters of conflict processing. We find that any emergent correlation in performance measures is heavily attenuated by variance in nonconflict processes such as strategy. Further, we observe correlations in performance of a similar magnitude when we impose correlations in nonconflict processes as we do when conflict processes are correlated. The implication of this is that the degree of behavioral performance correlation is not diagnostic of shared conflict processing between tasks: shared mechanisms could be masked, while behavioral correlations could be driven by other common processes (for example, a shared strategic approach).

## The Diffusion Model for Conflict Tasks

The DMC (Ulrich et al., 2015) is a mathematical model of choice RT behavior in conflict tasks, and an extension of the drift diffusion model (DDM; Ratcliff, 1978), a general model of choice RT behavior. The standard DDM assumes that individuals sample noisy evidence from their environment over time until a criterion level of evidence is reached for one of the two response options. The three main parameters describe the average rate of evidence accumulation (drift rate), the amount of evidence required (boundary separation), and the duration of motor and perceptual processes (nondecision time). Differences in difficulty between conditions are normally captured by differences in drift rate, with lower drift rates for stimuli that are less discernible.

The standard DDM assumes that the average rate of evidence accumulation within a trial is constant, albeit subject to random noise. This makes it unable to capture data patterns characteristic of conflict tasks, which have automatic response activation that conflicts with the desired response. First, errors in conflict tasks are typically fast in the incongruent condition (Gratton et al., 1988; Ridderinkhof, 2002), interpreted to reflect the automatic activation of the prepotent response. Second, whereas mean RTs in incongruent trials are typically slower than mean RTs on congruent trials in conflict tasks, the magnitude of this effect can vary, decrease, and even reverse when comparing the slower quantiles of the correct and incorrect RT distributions (especially in the Simon task; De Jong et al., 1994). This behavior is interpreted to reflect increasing influence of inhibition over time (or decay; Hommel, 1994), which acts to diminish and sometimes reverse the early influence of the automatic activation.

**Figure 1 fig1:**
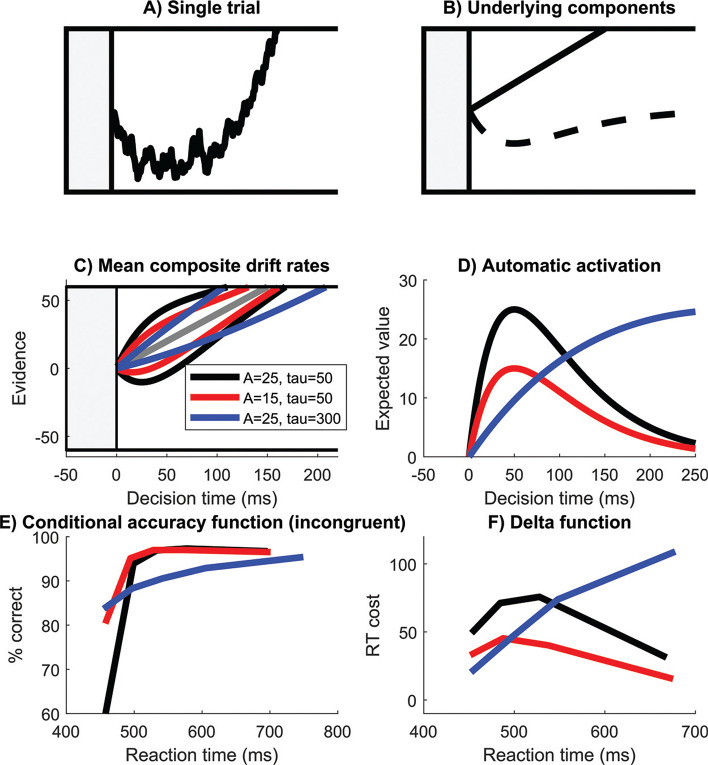
Schematic of the Diffusion Model for Conflict Tasks (Ulrich et al., 2015) *Note*. (A) The decision process is implemented as noisy accumulation of evidence to either the upper (b) or lower (−b) boundary, here representing the correct and incorrect responses respectively. Nondecision time (Ter) refers to sensory and motor processes, which occur before and after the decision phase. (B) The average rate of evidence accumulation is determined by two underlying process. The drift rate of the controlled process (μc) represents the efficiency of processing the task relevant property of the stimulus (e.g., the central arrow in a flanker task). The amplitude (A) and time-to-peak (tau) describe a rescaled gamma function, which represents the automatic activation and subsequent removal of automatic activation (e.g., the processing of the flanking arrows). Here the automatic activation is depicted for incongruent trials (it is reversed for congruent trials). (C) Mean evidence accumulation rates for different values for the amplitude and time-to-peak. The central gray line reflects a controlled drift rate of .4. Colored lines above and below the gray line reflect congruent and incongruent trials respectively (combined controlled and automatic processing). Increasing the amplitude leads to a greater deviation from the central line. Increasing the time-to-peak affects the time at which the maximum deviation is reached. (D) Automatic activation functions for different values for the amplitude and time-to-peak. The amplitude parameter rescales the distribution to a specified maximum. Note the maximum value of the automatic activation may occur later than the typical decision time. (E) Conditional Accuracy functions (CAFs) corresponding to panel C. CAFs show the accuracy of responses in quantiles of the reaction time distribution. The black vs. red (dark gray) line shows the effect of increasing the amplitude parameter. This increases the proportion of fast errors made in incongruent trials, reflecting an increase in response capture. Increasing the time-to-peak leads to errors being more distributed across the RT distribution, reflecting a slower removal (inhibition) of the automatic activation. (F) Delta functions corresponding to panel C. Delta plots show the RT cost at different quantiles of the RT distributions. Increasing the amplitude parameter leads to increased mean RT costs (higher average values of the delta functions on the *y* axis). Increasing the time-to-peak produces more positive going delta slopes, shown by the blue (light gray) vs. black lines. Note the correspondence between the shape of the delta functions and the shape of the automatic activation that produce them (Figure 1D). See the online article for the color version of this figure.

The DMC (Figure 1A–1C) accounts for conflict effects by assuming that the task-irrelevant feature (for example, the flankers in a flanker task) is processed via a fast and automatic route that initially receives a strong activation which is reduced over time. Concurrently, the task-relevant feature (the central arrow in a flanker task) is processed via a slower, deliberate decision route. The controlled route is captured by a drift rate parameter that is held constant over congruency conditions in the DMC. This reflects the assumption that the processing of the task relevant property of the stimulus is equivalent across all conditions. The drift rate parameter in the DMC can therefore be interpreted as general processing efficiency. The automatic route is implemented as a rescaled gamma function, which captures the assumption that prepotent stimulus features influence the early phase of the decision processes more than the later phase (Figure 1D).[Fig fig1]

The DMC takes inspiration from the Activation-Suppression hypothesis (De Jong et al., 1994; Kornblum, 1994; Ridderinkhof, 2002), which posits that the automatic activation is removed through active suppression. However, the DMC is agnostic about what drives the reduction in the influence of automatic activation and has no explicit parameter to represent inhibitory ability. Instead, the ability to overcome conflict is implicit in the degree of susceptibility to prepotent response activation (the amplitude it reaches), and the speed at which automatic activation peaks and is removed/decays. The maximum value of the automatic activation is defined by an amplitude parameter, and the time that the maximum value is reached is defined by a scale parameter—we hereafter refer to the scale parameter as the time-to-peak (following Ulrich et al., 2015).^1^ The gamma function also has a shape parameter, but following Ulrich et al. (2015; see also White et al., 2018) we fixed this to a constant value for all individuals. Therefore, individuals with more efficient inhibition would be expected to have either a lower amplitude and/or a shorter time to peak as these are the parameters that should capture individual differences in conflict processing (Figure 1E and 1F).

We note that our approach here is one of model application, rather than model validation or comparison (Crüwell et al., 2019). We adopt an evidence accumulation framework on the basis of previous demonstrations that they can inform our understanding of individual differences in cognitive abilities in the context of the confounds we have mentioned (Hedge et al., 2018; Ratcliff et al., 2015). Our criteria for selecting an appropriate model were that it has parameters that represent conflict processing, and that it can provide a common framework for all our tasks. The DMC meets these criteria and has previously been applied to both the flanker and Simon tasks (Servant et al., 2016; Ulrich et al., 2015). Since we began this work it has also been applied to the color-word Stroop task (Ambrosi et al., 2019; Hedge et al., 2019). The model could theoretically also be applied to other tasks that show the data patterns that are characteristic of conflict tasks, including the Navon task (fast errors; Hübner, 2014), as well as in the antisaccade task (fast errors and negative delta functions; von Bastian et al., 2020; Wiecki et al., 2016). Alternatives we considered are not capable of producing negative delta functions (Hübner et al., 2010; White et al., 2011) and have parameters that represent task specific processes rather than general conflict processing (for example, spatial attention; White et al., 2011). An alternative model might provide a better theoretical account or empirical fit to certain tasks, although a full comparison is beyond the scope of this article. For our goal of examining whether parameters that capture conflict correlate across tasks, we assume that they can be meaningfully captured within the common framework of the DMC.

## Part I. Are Measures of Conflict Correlated Across Tasks?

### Rationale

The first question is whether model parameters can reveal correlations between conflict tasks—evidence for common mechanisms—that traditional measures are less able to detect. We answer this question by performing a meta-analysis of 12 task pairs taken from seven datasets including new and previously published data (Hedge et al., 2018; Hedge et al., 2018b; Hedge et al., 2019; Whitehead et al., 2019). We fit the DMC to each task and participant separately to extract model parameters.

### Datasets

We selected datasets by updating the available datasets in our recent systematic review (Hedge et al., 2018) and applying the following criteria: (a) They include some combination of the flanker, Simon, color Stroop or spatial Stroop tasks, which have analogous conflict effects suited to modeling in the DMC framework (cf. Ulrich et al., 2015); (b) They have trial level data with at least 200 trials per condition to ensure adequate parameter estimation, based on a parameter recovery simulation using the DMC (White et al., 2018).

**Figure 2 fig2:**
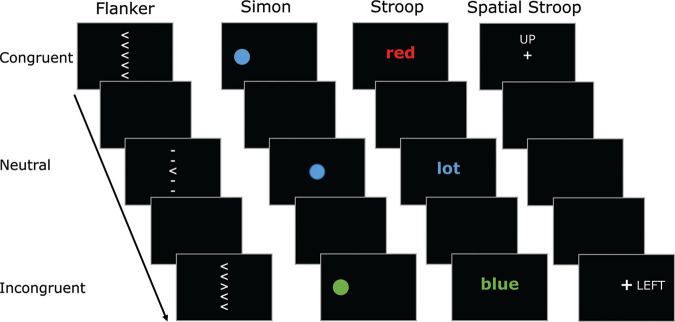
Schematic of Tasks *Note*. In the flanker task (Datasets 1, 2, and 4), participants respond to the central arrow and ignore the flankers. In the Simon task (Datasets 1 and 3), participants respond to the color of the stimulus and ignore the location. In the Stroop task (Dataset 2 and 4), participants respond to the color of the font and ignore the written word. In the spatial Stroop task (Datasets 5–7, referred to as a Simon task by Whitehead et al., 2019), participants respond to the meaning of the written word and ignore its location. Whitehead et al. did not include neutral conditions, so we do not illustrate one for the spatial Stroop. The flanker task in Datasets 5–7 consisted of horizontally distributed letters (e.g., DDDDD, FFKFF) instead of arrows. The flanker and Simon tasks in Datasets 1–4 were two-choice tasks, and all others were four-choice. See the online article for the color version of this figure.

Table 1 summarizes the key information of each dataset, and a schematic is shown in Figure 2. For full methodological details, see Supplementary Material A in the online supplemental materials and the original articles. We draw particular attention to Dataset 3 (Hedge et al., 2018), which consists of two variants of the Simon task. In one variant, congruent and incongruent trials were randomly intermixed (as is standard for the Simon task), whereas in the other congruent and incongruent trials were presented in separate blocks (a common format for the antisaccade task). Thus, surface features are matched, and any processing differences would be introduced by the blocking arrangement. We also note that the tasks in Dataset 4 (Hedge et al., 2019) consisted of separate blocks that instructed participants to emphasize speed, accuracy, or both speed and accuracy.[Table tbl1][Fig fig2]

We collected a self-report measure of impulsivity (the UPPS-P; Lynam et al., 2006) alongside Datasets 1–4, because we were interested in whether trait impulsivity or cautiousness correlated with response caution in the DMC. We report the results of this analysis elsewhere (Hedge, Powell, et al., 2020); briefly, we observed no evidence for a correlation.

### Data Analysis

We applied the same data analysis procedure to all datasets. We excluded participants who were below 60% accuracy in any task in each dataset (lenient in order not to limit variance; Supplementary Material B in the online supplemental materials shows a more conservative cut-off of 80% does not alter our conclusions). We removed RTs that were less than 100 ms and greater than the median plus three times the median absolute deviation for each individual in each condition.

See Appendix A for the technical details of our model fitting approach, which is identical to previously published work (Hedge et al., 2019) and similar to common approaches to fitting evidence accumulation models (Vandekerckhove & Tuerlinckx, 2008; White et al., 2018). Our code is available online (https://osf.io/4c3we/).

### Meta-Analysis of Correlations

We calculated Spearman’s rho correlations for each model parameter for each pair of tasks (e.g., the correlation between the amplitude parameter from the flanker task in Dataset 1 with the amplitude parameter from the Simon task in Dataset 1). This produced 13 correlations for each parameter (15 for boundary separation, as we calculated separate boundary values and correlations for each of the three instruction conditions in the Dataset 4). These correlations were then metaanalysed using a multilevel random effects meta-analysis, implemented in the metafor package in R (R Core Development Team, 2017; Viechtbauer, 2010). The multilevel approach allows us to account for the possibility that correlations taken from the same dataset (as with Datasets 4 to 7) may be more similar to each other than correlations taken from independent datasets. In Supplementary Material B in the online supplemental materials, we also account for the possibility that the correlation in certain task pairs (e.g., spatial Stroop and Stroop) is higher than in other pairs (e.g., flanker and Stroop). This does not alter our conclusions, and we report the simpler analysis here due to the limited number of data points.

We also calculated the I^2^ statistic for each parameter (cf. Viechtbauer, 2019), which is interpreted to represent the heterogeneity of the observed effects. An I^2^ of 0% would indicate that all the variability in the observed effect size estimates is due to sampling error, rather than real differences between datasets and task pairs. We interpret I^2^ values of 25%, 50%, and 75% as low, moderate, and high levels of heterogeneity, respectively (Higgins et al., 2003).

Given that the literature does not find consistent correlations between tasks (Rey-Mermet et al., 2018), it is likely that if a correlation between conflict parameters exists then the effect size would be small. We conducted a sensitivity power analysis to ascertain the strength of correlation that our meta-analysis is able to detect (Pigott, 2012), based on our number of observed effect sizes and average sample size. Assuming either low, moderate, or high levels of heterogeneity, we have 80% power to detect average correlations of *r* = .07, *r* = .09, and *r* = .12, respectively. In other words, we are sensitive to most effect sizes traditionally considered small (*r* = .1, Cohen, 1988).

### Results and Discussion

#### Meta-Analysis of Model Parameters

**Figure 3 fig3:**
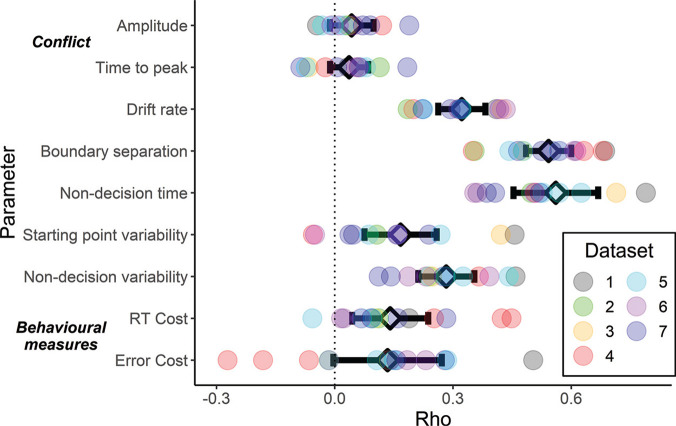
Meta-Analytic (Black Diamonds) and Observed (Circles) Zero-Order Correlations Between Tasks in Parameters of the Diffusion Model for Conflict Tasks (DMC) *Note*. We also plot the traditional behavioral metrics of reaction time (RT) costs and error costs. Error bars show 95% confidence intervals. Because we used zero-order correlations, and some datasets had multiple tasks (5–7) or speed/accuracy conditions (4), these datasets contribute multiple circles of the same color to the plot. A multilevel random effects meta-analysis was performed on Spearman’s rho correlations calculated for each pair of tasks, allowing for clustering where multiple correlations were taken from the same dataset. The Amplitude and time to peak parameters are associated with conflict processing. See the online article for the color version of this figure.

Our main question concerns the correlations between tasks for the model parameters (see Figure 3). We report the results of this analysis first, before considering factors that might moderate our conclusions, such as the reliability of the data and model fits. If we assume that factors such as general processing speed and strategy confound behavioral measures of inhibition, then separating these out using a cognitive model may reveal correlations in the parameters representing conflict processing—the amplitude and time-to-peak of automatic activation. Figure 3 shows the weighted average correlation for each parameter, along with the individual correlations for each pair of tasks.[Fig fig3]

We observed a very small and nonsignificant positive correlation for both the amplitude parameter (*r =* .04, 95% CI [−.01, .10], *p* = .13, *I*^2^ = 18.5%) and the time-to-peak parameter (*r =* .04, 95% CI [−.01, .08], *p* = .14, *I*^2^ = 20.5%). Note from the *I*^2^ values that the estimated heterogeneity is low (<25%), which is also reflected in the narrow range of *r* values in Figure 3. These correlations correspond to less than 1% of common variance on average, providing no support for the hypothesis of a common mechanism of conflict processing between tasks. The low I^2^ values suggest this to be the case consistently across all datasets. We again draw particular attention to Dataset 3, which did not deviate from the trend of low correlations in amplitude (*r =* .04) and time-to-peak (*r = −*.07) despite consisting of the same Simon task performed with intermixed and blocked trials.

In contrast to the weak correlations observed for the conflict parameters, estimates for the nonconflict parameters were consistently positive and statistically significant. In particular, we observed moderate to strong correlations in drift rate (*r =* .32, 95% CI [.26, .38], *p* < .001, *I*^2^ = 33.6%) and boundary separation (*r =* .54, 95% CI [.49, .60], *p* < .001, *I*^2^ = 50%). These parameters represent the efficiency of processing (i.e., general processing speed) and response caution, respectively. Finally, we also observed significant positive correlations in the mean (*r =* .56, 95% CI [.45, .67], *p* < .001, I^2^ = 85.6%) and variability of nondecision time (*r =* .28, 95% CI [.21, .35], *p* < .001, I^2^ = 57.1%), as well as in start point variability (*r =* .17, 95% CI [.08, .26], *p* < .001, I^2^ = 72.9%). The model parameter correlations therefore provide good evidence for commonality in the mechanisms underlying general performance in conflict tasks, but not for the conflict and inhibition processes themselves.

#### Behavioral Performance

For completeness, we applied the same meta-analytic approach to the traditional behavioral indicators of conflict processing: the RT costs (*r =* .14, 95% CI [.04, .24], *p* = .004, *I*^2^ = 64.4%) and error costs (*r =* .13, 95% CI [−.00, .27], *p* = .056, *I*^2^ = 83.1%). These are plotted at the bottom of Figure 3. It is notable that both showed positive correlations of a similar magnitude, with the RT cost reaching significance, though the effect sizes are small and heterogenous.

In all tasks, we observed the expected pattern of increased error rates and slower RTs in incongruent trials relative to congruent trials (Supplementary Material C in the online supplemental materials).

#### Reliability and Parameter Recovery

Weak correlations in model’s conflict parameters could reflect instability in the parameter estimates. This is plausible, for two reasons: (a) conflict parameters are essentially derived from differences between conditions, and differences are typically less reliable than their components (Cronbach & Furby, 1970); (b) cognitive tasks developed initially for within-subject analyses have to some degree been naturally selected for low between-subjects variance in the mechanisms of interest, which causes reliability to be lower in correlational research (Hedge, Bompas et al., 2020; Hedge et al., 2018b; Miller & Ulrich, 2013).

We evaluated the parameter recovery of the model for our empirical fits (see Appendix B), as well as the split-half reliability of our behavioral measures (for full details, see Supplementary Material C in the online supplemental materials). Across all tasks and datasets, we observed sufficient recovery of the amplitude parameter (median *r* = .84) and the main nonconflict parameters: drift rate (median *r =* .93) and boundary separation (median *r =* .94). Our ability to detect correlations in the time-to-peak parameter is likely to be limited by its poor recovery outside of the Simon and spatial Stroop tasks (median *r =* .48).

We have also previously examined the four week test–retest reliability of the DMC parameters in Dataset 4 (Hedge et al., 2019). Consistent with our parameter recovery exercise here, the amplitude parameter showed moderate reliability (ICC = .55 and .47 in the flanker and Stroop task, respectively), and the reliability of the time-to-peak parameter was poor (ICC = −.04 and .19). For comparison, these fall within the ranges seen for the reliabilities of the RT costs (ICCs ranging from .38 to 66) and error costs (ICCs from .09 to .53) in these tasks. Drift rate (ICC = .77 and .48) and boundary separation (ICCs ranging from .39 to .71) tended to show similar or better reliability than the conflict parameters. Note that we had a total of six separate behavioral costs and boundary estimates in this study, corresponding to the three speed–accuracy trade-off instruction conditions in each task.

#### Model Fits and Sanity Checks

We report the means and standard deviations for the model parameters in Appendix C. For the two-choice tasks in Datasets 1–4, parameters were similar to those reported using comparable tasks (Ulrich et al., 2015). We observed slower RTs in the four-choice tasks (Stroop, Datasets 5–7), which corresponded to increases in average boundary separation and nondecision time, and a decrease in drift rate and the amplitude of automatic activation. The time-to-peak of automatic activation values were similar for different variants of commonly named tasks (e.g., the two-choice flanker and the four-choice flanker) and followed the expected pattern of being shortest for the Simon tasks and longest for the Stroop.

**Figure 4 fig4:**
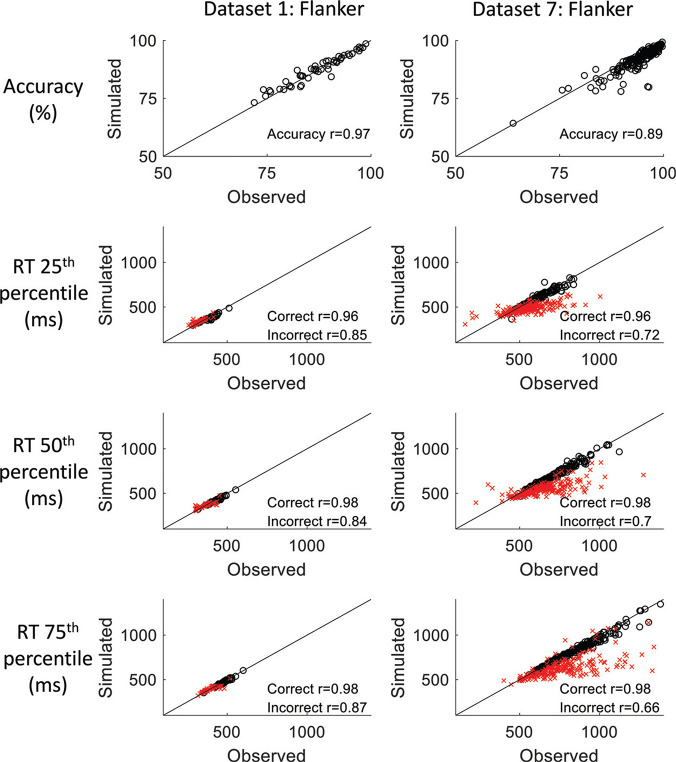
Scatter Plots Showing Fits for Incongruent Trials in the Flanker Task in Dataset 1 (Two-Choice, Left Column) and 7 (Four-Choice, Right Column) *Note*. We chose these for illustration because Dataset 1 shows a good fit whereas Dataset 7 shows a clear underestimation of the speed of slow RTs. We calculated Pearson correlations for accuracy (top row) and RT quantiles (25th, 50th, 75th; second, third, and fourth row respectively) of the observed data against data simulated using the best fitting model parameters for each participant. For RTs, black circles represent correct responses, red crosses represent errors. A good fit is indicated by a strong positive correlation and a tight clustering of the points around the diagonal identity line. Note that in the right column, the red crosses cluster below the identity line, indicating that errors produced by the model fits tend to have lower RTs in a more restricted range than is observed in the data. Despite this underestimation, the correlations between observed and simulated data are reasonably strong. See the online article for the color version of this figure.

If the DMC is an appropriate model for these tasks, then the best fitting parameters should reproduce both individual differences in the data and capture key data patterns. We evaluated the model fits by calculating Pearson correlations for accuracy and RT quantiles (25th, 50th, 75th) of the observed data against data simulated using the best fitting model parameters for each participant (Voss et al., 2015). RTs for correct and incorrect responses were evaluated separately. We illustrate this with incongruent trials from two tasks in Figure 4, which are representative of the range of fits we observed. In addition, we evaluated the extent to which the fits could qualitatively reproduce the conditional accuracy functions and delta plots in the observed data. We report the correlations and figures in Appendix C and Supplementary Material F respectively and focus here on the implications for our interpretations of the model parameters.[Fig fig4]

Focusing first on individual differences, the model fits generally captured accuracy well. The minimum correlation between observed and simulated accuracy for any task/dataset were *r =* .73 and *r =* .86 for congruent and incongruent trials, respectively. Correct RTs were also captured well across all RT quantiles for congruent (minimum *r =* .85) and incongruent trials (minimum *r =* .91). The reproduction of RT for error trials showed more variability, ranging from .61 to .96 for incongruent trials. This is to be expected as error RTs are based on fewer trials, so the estimates are noisier. Notably, the model tended to systematically underestimate RTs for tasks that had slower RTs overall, particularly for errors (Stroop, Datasets 5–7; see Figure 4).

A consequence of the underestimation of slow incongruent RTs was the underestimation of the RT cost in tasks with slower (correct) RTs. We elaborate on this behavior in Supplementary Material D in the online supplemental materials and consider the theoretical implications of these patterns in the discussion. A consequence for our meta-analysis is that the DMC parameters may be poorly estimated for these tasks where the data are less-well captured. This could contribute to the small correlations seen in the conflict parameters in Figure 3. We opted to include all the datasets in our meta-analysis despite this observation. We reasoned that the pattern of fast errors in most tasks was reflected in the model fits, which indicates that they are capturing the timing and strength of conflict effects to some degree. Further, the strong positive correlations in accuracy and RT quantiles indicate that individual differences are being captured by the model. The consistency of the conflict parameter correlations observed in our meta-analysis, indicated by the low *I*^2^ values, suggests that our conclusions are not dependent on the inclusion of particular datasets.

#### Representativeness of Datasets

The datasets included in our modeling were selected to have larger trial numbers than is normally seen in the literature. We might question whether this criterion or the limited number of sources (two labs, including our own) affects the representativeness of correlations seen in these datasets. A recent analysis by von Bastian et al. (2020) surveyed between-task correlations for “attention control” tasks, including the conflict tasks we examine here, and others such as n-back and working memory updating. The median correlation between all task pairs was *r =* .16 (*n* correlations = 2114), and correlations were typically lower when pairs included at least one of the flanker, Simon or Stroop tasks. This overall value is similar to the averages we observe in our meta-analysis of RT costs (*r =* .14) and error costs (*r =* .13). von Bastian et al. further note that most correlations did not exceed *r =* .3. Similarly, most of our behavioral correlations fell between *r* = 0 and *r =* .3, with a few exceeding this (min *r = −*.27, max *r =* .50; see Figure 3). Thus, the correlations in our datasets appear to be representative of those seen in the broader literature.

#### Summary of Empirical Data

Overall, we observe weak or no correlation between tasks in DMC parameters representing conflict processing. However, we do observe consistent correlations in model parameters reflecting nonconflict decision processes. We see small but significant correlations in RT costs, although these could also be driven by common variance in strategy and processing speed across tasks. A critical step toward interpreting these effects is to understand the source(s) of individual differences in these measures.

## Part II. Could Performance Measures Diagnose Shared Conflict Mechanisms?

We might interpret the weak correlations between parameters of conflict processing in our datasets as an indication of independent mechanisms underlying each task. However, a domain-specific account of conflict control is difficult to apply to Dataset 3, where the intermixed and blocked variants of the Simon task share surface characteristics. Although we expect trial arrangement and proportions to affect the processing demands of a task (Unsworth et al., 2004), there ought to be at least some degree of common conflict processing for the incongruent trials in blocked or random arrangement. But we observed no better correlation than for other task pairings, suggesting that it is difficult to isolate individual differences in conflict processing among other processes that contribute to behavior.

Despite the absence of correlations in conflict model parameters, we did observe a small but significant positive correlation in RT costs, as well as a similar correlation in error costs. Can these correlations provide evidence of common conflict-processing mechanisms? We know they are not perfect evidence, as performance costs do not isolate ability in a specific cognitive domain (Hedge et al., 2018; Hedge et al., 2018a; see also Draheim et al., 2016; Miller & Ulrich, 2013). However, this is not to say that they carry no information. In Part II, we evaluate this through simulation.

First, we ask whether detectable correlation in task performance is a necessary consequence of underlying common conflict-processing mechanisms. In other words, when we impose a correlation in conflict parameters in the model, how does this manifest in behavioral correlations in RT costs and/or error costs (when participants vary randomly in other ways)?

Second, we ask whether correlation in performance measures is sufficient evidence of common conflict-processing mechanisms. In other words, are correlations in RT costs and error costs driven just as well by shared nonconflict processes?

We conducted a set of simulation studies to assess these questions. We imposed correlations in conflict model parameters (amplitude and/or time-to-peak) between two tasks to represent a common mechanism for conflict. We then compared this with an alternative, in which there are no correlations in conflict parameters, but the nonconflict decision parameters (drift rate and boundary separation) were correlated instead. We tested how these underlying structures would emerge in RT costs and error costs. Our simulations have the additional benefit that we are not limited by measurement noise attributable to low trial numbers or reliability, so this approach provides a theoretical upper limit for the effect sizes we could expect to see in real data.

### Method

We based our parameter ranges on a previous parameter recovery study (White et al., 2018), which themselves were based on previous studies that had applied the DMC (Servant et al., 2016; Ulrich et al., 2015). White et al. observed high correlations between simulated and recovered parameters (*r* > .93 for all parameters when shape is held constant), so we can be confident that these ranges produce discriminable variation in behavior.

We simulated multiple scenarios that varied on three dimensions. The first dimension reflected different hypothetical tasks. We simulated hypothetical Simon, flanker, and Stroop tasks by varying the average value of the time-to-peak parameter to match what we observed in our model fits. We did this because this parameter has previously accounted for differences in behavioral patterns between tasks (Ulrich et al., 2015), and we reasoned that these different dynamics may affect the correlations observed in RT cost and error costs. For simplicity, and to maintain the approach of testing the upper limit of correlations we would expect in real data, we used the same means and standard deviations for the parameters in both simulated tasks within each scenario (i.e., we test for correlation between two versions of the same task). We also used the same mean and variance for the other parameters across all tasks to aid comparisons (see Table 2). We report correlations across different simulated tasks in Supplementary Material E in the online supplemental materials. As expected, these were generally smaller than those we report here, but they followed the same patterns.[Table tbl2]

The second dimension that we varied across scenarios was which mechanisms had correlations imposed across tasks in the underlying model. We imposed a common conflict-processing mechanism in three ways: a correlation in the amplitude parameter only, the time-to-peak parameter only, and both the amplitude and the time-to-peak parameters. In the fourth scenario, the conflict parameters were uncorrelated, and we imposed correlation in drift rate and boundary separation. We assumed no correlation (*r* = 0) for all parameters other than those named in each scenario.

The third dimension that we varied was the magnitude of the correlation that we imposed (*r* = .3, .5, and .7). We did this to evaluate whether RT costs and error costs were sensitive to changes in correlation in the underlying mechanisms.

For each scenario and effect size, we simulated datasets for 2,000 participants comprising 5,000 congruent and 5,000 incongruent trials each. This is more trials than would typically be run in an empirical study, but it allows us to minimize the impact of noise on our estimates. We expect behavioral correlations with lower trial numbers would be smaller. Parameters were generated from a multivariate normal distribution using Matlab’s *mvnrnd* function. This allows for the generation of two variables with specified means, standard deviation, and covariance (correlation). We derived the standard deviations by dividing the range of the uniform distributions used by White et al. (2018) by six, to obtain a similar range. In other words, the upper limit of the uniform distribution used by White et al. corresponds to three standard deviations above the mean of the normal distribution used in our simulation. For simplicity we did not include variability in nondecision time, and we fixed the shape parameter for automatic activation to 2, as in our empirical fits and Ulrich et al. (2015).

### Results and Discussion

#### Performance Correlations Are Not Necessary Evidence for Common Mechanisms of Conflict Processing

**Figure 5 fig5:**
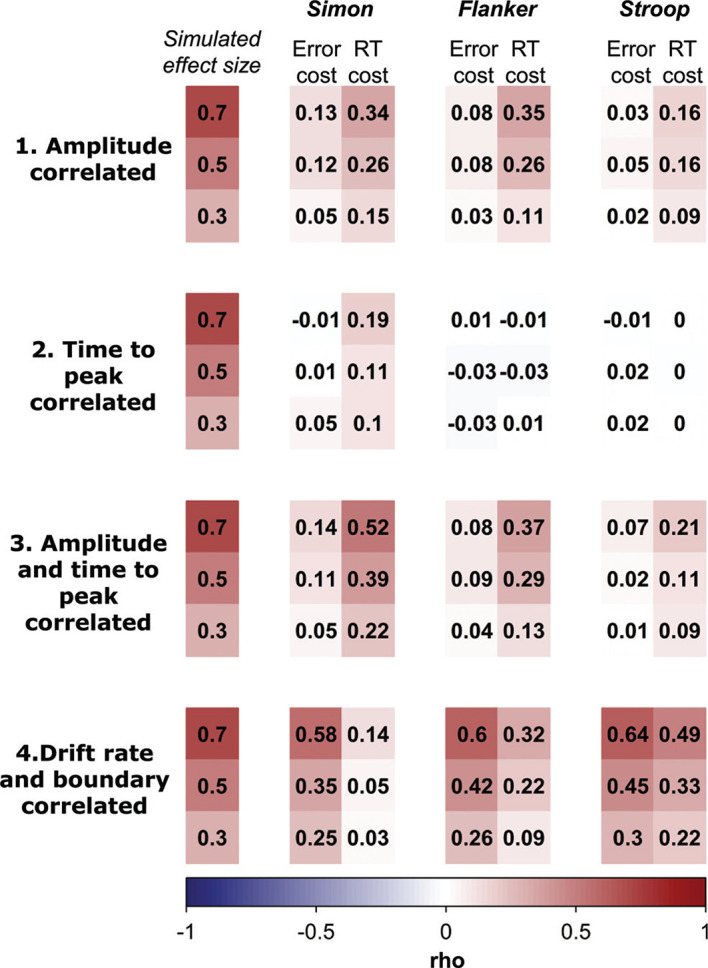
Spearman’s Rho Correlations Between Performance Costs Calculated From Two Simulated Datasets Using the Diffusion Model for Conflict Tasks *Note*. The strength of the between-task correlation in the model parameter(s) is given in the “Simulated effect size” column. The columns to the right of this show the between-task correlations in the simulated error and RT costs, respectively. The correlation between other model parameters (boundary separation, drift rate, and nondecision time) was set to 0 in the first three scenarios. In the fourth scenario, the correlation in conflict parameters was set to zero, and the nonconflict parameter correlations were varied. We used the same parameter ranges for both tasks within each scenario. For example, the Simon column shows the correlations between two versions of a Simon task. Note that the size of the correlations in the fourth scenario are comparable to, and in some cases exceed, those observed in the first three scenarios. See the online article for the color version of this figure.

Spearman’s rho correlations between performance measures calculated from the two simulated tasks are shown in Figure 5. First, we evaluated whether correlations in performance are a necessary outcome of introducing correlations in the model conflict parameters. The white/pale sections in the first three scenarios (see Figure 5) illustrate that this condition is not met. It was possible to observe no correlation in both RT costs and error costs in the presence of very strong (*r* = .7) correlations in the time-to-peak parameter.[Fig fig5]

The correlation in RT costs generally increased as the underlying correlation in the amplitude parameter increased and were largest in the scenarios where correlations were imposed in both the amplitude and time-to-peak parameters. However, the behavioral correlations were heavily attenuated in some cases, and to different degrees in different tasks. For example, whereas a correlation of (*r =* .52) was observed in RT costs in the Simon task when the correlation in both amplitude and time-to-peak was very strong (*r =* .7), the corresponding correlation in the Stroop scenario was small (*r =* .21). This occurs because independent variance in the nonconflict parameters masks the effect of the conflict parameters and does so to different degrees depending on the temporal dynamics of the conflict process in each task. This pattern could lead researchers to incorrect conclusions about shared mechanisms across different types of task; correlations can be smaller simply because of slower activation of the conflict process, not necessarily because of more independence. Note that most correlations in RT and error costs predicted in the first three scenarios are below what is traditionally considered moderate (.3), except when the correlation in amplitude is very large (.7), or both the amplitude and time to peak parameters show strong (>.5) correlations. Based on our empirical fits, where the largest correlation we saw in conflict parameters in any dataset was *r =* .19, we do not expect underlying correlations in currently used tasks to be strong.

#### Performance Correlations Are Not Sufficient Evidence for Common Mechanisms of Conflict Processing

Next, we evaluated whether it is possible to observe correlations in RT costs and error costs in the absence of common mechanisms of conflict processing. In the fourth scenario (see Figure 5), the mechanisms underlying conflict processing are independent (*r* = 0), but we imposed correlations in parameters representing strategy and general processing efficiency. The key observation here is that the correlations can be similar to, and even exceed, those we see in the first three scenarios. This illustrates that nonconflict processes (e.g., strategy, processing speed) can create correlations in measures of inhibition when the mechanisms of conflict processing are in fact independent.

The magnitude of the correlations we observe in the fourth scenario may surprise some readers, although they are in line with previous simulations (Hedge et al., 2018; Hedge et al., 2018a). The reason is that both RT costs and error costs are correlated with drift rate and boundary separation, and we impose a correlation on both these parameters simultaneously here, so they have a strong impact on behavior. We show the correlations between the behavioral measures and parameters in Supplementary Material E in the online supplemental materials.

#### Caveats and Considerations

A key inference from our simulations is that individual differences in nonconflict decision processes could mask individual differences in conflict processing in performance measures. In our first three scenarios, our simulated individuals varied in boundary separation and drift rate, but this variation was uncorrelated between tasks, and therefore adds noise to the performance measures. The extent of noise is dependent on the standard deviations used to generate the parameters (see Table 2). Smaller standard deviations for nonconflict parameters would allow stronger correlations in performance measures to emerge as a function of the conflict parameters. The standard deviations we chose were based on previous simulations (White et al., 2018) and empirical observations (Ulrich et al., 2015). Are they too large? In fact, we observed greater variance, not less, in several parameters in the fits to our data (see Appendix C). To check the robustness of our conclusions, we conducted an additional simulation in which we generate parameter sets using the means and standard deviations we observed in the DMC fits to our flanker, Simon and color-word Stroop data (Supplementary Material E in the online supplemental materials). The resulting between-task correlations in simulated performance measures did not exceed those reported for the analogous scenarios in Figure 5. Thus, our interpretation that shared conflict processing would have a relatively small effect on behavior is not specific to the source of simulated parameter ranges.

A second consideration is that we simulated the scenarios of shared conflict or nonconflict mechanisms in isolation. When we assumed that the amplitude and time-to-peak parameters were correlated, we assumed that drift rate and boundary separation were uncorrelated and vice-versa. In reality these are not mutually exclusive—it is possible that both conflict and nonconflict processes are correlated in some scenarios, both of which contribute to positive correlations in performance costs. However, the challenge faced by researchers remains the same: The magnitude of correlations in RT costs or error costs cannot be interpreted as the degree of shared conflict processing or inhibition.

We reiterate that our simulations represent scenarios where the underlying variance is not restricted (because the parameters can be recovered well; White et al., 2018), where the variance is similar between the two tasks, and where there is minimal noise in the behavioral measures due to the large number of simulated trials. Thus, if the model is an appropriate one, the results represent the upper limit of what would be expected in real data. For example, in Figure 5, we see that large correlations in nonconflict processes lead to moderate correlations in error costs. However, despite our empirical meta-analysis showing that moderate to large correlations are present in strategy and processing speed in real data, the corresponding average correlation in error costs is small. Error rates are often low in empirical data, making them difficult to measure reliably. As we and others have previously noted, poor reliability and low trial numbers can make it difficult to draw conclusions from small correlations (Hedge et al., 2018b; Miller & Ulrich, 2013; Rouder et al., 2019).

#### Summary of Simulations

Correlations in conflict parameters do not always translate into behavioral congruency effects. On the other hand, correlations in nonconflict parameters can produce large correlations in behavioral congruency effects. Taken together, correlations in performance costs are neither necessary nor sufficient to infer there are common underlying conflict-processing mechanisms.

## General Discussion

The overarching questions we address here are: *is there a common mechanism of conflict processing underlying performance across ‘inhibition’ tasks and, if there were, would we be able to detect it from RT and error costs?* Our data and simulations suggest the presence or absence of correlations across conflict tasks is only weakly informative as to whether common conflict control mechanisms underlie performance.

The meta-analysis of model parameters fit to multiple empirical datasets, parameters associated with conflict processing correlated weakly or not at all. This pattern persists even when we examine two variants of the same task, which we assume share more common elements than tasks from different conflict domains.

Our simulations indicate that it might be difficult to detect behavioral correlations even if shared conflict mechanisms exist, and that the degree of behavioral correlation cannot be specifically attributed to the degree of shared conflict processing. Parameters reflecting response caution and general processing efficiency contribute substantially to performance measures. In the presence of correlated conflict parameters, these nonconflict parameters add noise if they are uncorrelated between tasks, potentially leading us to conclude that conflict processing mechanisms are relatively independent. Alternatively, if these general processes are correlated between tasks—as they seem to be in the datasets presented above—they drive correlations in performance measures and could mislead researchers searching for common conflict mechanisms.

### Should We Stop Thinking About Individual Differences in Inhibition?

The construct of response control or response inhibition has been a core component of cognitive theorizing for at least several decades (Logan et al., 1984; Miyake et al., 2000) and one that has been heavily implicated in neuropsychological disorders and brain dysfunction (Bari & Robbins, 2013; Chambers et al., 2009). Rey-Mermet et al. (2018) pose the question of whether inhibition is a useful psychometric construct, citing low and inconsistent correlations reported in the literature and their own data. Instead, they suggest that the ability to resolve interference is task specific, challenging the often-made assumption that performance on any given response control task can be interpreted in a broader context. Our findings are consistent with this position but highlight that it is very difficult to draw any conclusions about inhibition constructs from the degree of behavioral correlations.

One clear finding from our meta-analysis was that we consistently observed little correlation in conflict-related model parameters. We could interpret this as evidence for modality-specific mechanisms; however, we still could not detect correlation between conflict parameters in our intermixed and blocked versions of the Simon task (Dataset 3). One explanation for this is that our blocking manipulation changed the way the stimuli were processed (Gehring et al., 1992; Hedge et al., 2018; Unsworth et al., 2004), to the point where automatic process are engaged differently by individuals in each context. We do not assume to have equated the way the stimuli are processed by changing only the blocking format, and we treat them as independent tasks in our fitting. Our assumption is that if there is a common inhibitory ability that manifests across tasks that differ in their blocking format *as well as* their stimulus features and response format, then a dataset with fewer differences is a low hanging fruit for observing correlations (for a similar approach, see Snyder et al., 2019). That we do not observe a correlation when using two versions of (nominally) the same task has implications for studies that attempt to correlate different tasks that typically use blocked trials (e.g., the antisaccade) with tasks that typically intermix them (e.g., flanker, Simon, Stroop).

The absence of correlations between two variants of the Simon task also raises the consideration of how perhaps seemingly neutral differences in task implementation can change what our tasks are measuring. Factor analytic studies of inhibition often include multiple versions of a flanker task (e.g., using letters or arrows; Kane et al., 2016; Rey-Mermet et al., 2018; Rey-Mermet, Gade, Souza, et al., 2019) or Stroop-like tasks (e.g., color-word, number, spatial; Chuderski et al., 2012; Kane et al., 2016; Pettigrew & Martin, 2014; Rey-Mermet et al., 2018; Rey-Mermet, Gade, Souza, et al., 2019; Salthouse & Meinz, 1995; Shilling et al., 2002). However, there is limited evidence for higher correlations between these commonly named tasks than between differently-named inhibition tasks in young adults (for a discussion of the Stroop, see Rey-Mermet et al., 2020). There has been recent interest in how design (e.g., trial numbers) and analysis choices impact the reliability of a measure (Hedge et al., 2018b; Parsons, 2020; Parsons et al., 2019; Rouder & Haaf, 2019; von Bastian et al., 2020), and a similar approach to validity would improve our ability to construct a task in a way that maximally captures the process(es) that we are interested in. This could be done by systematically varying features of the task design (cf. Baribault et al., 2018), in combination with modeling how these affect the relative contribution of different underlying processes.

Alternatively, we could conclude that it is simply too difficult to recover meaningful information about conflict from correlating tasks (Rouder et al., 2019). We believe that models are a useful tool for individual difference research, but that they are not a panacea (Hedge, Bompas et al., 2020). We have shown here that correlations in nonconflict processes can confound the correlations we observe in behavior, so there is a benefit to separating these out from conflict processes. Further, although we cannot expect to simply sidestep the reliability problems associated with difference scores (Hedge et al., 2018b; Miller & Ulrich, 2013) by replacing them with model parameters that account for those same differences, there is a potential for improvement by utilizing more information from the data we collect, including the simultaneous modeling of both accuracy and the shape of RT distributions. However, cognitive models should not be expected to create reliable individual differences in tasks that are not suited to eliciting them (Hedge et al., 2018b). If common mechanisms of inhibition do exist, they appear to be too fragile to detect in the context of individual differences in other mechanisms in our current tasks, such as those related to caution and processing speed.

The answer to the question of whether we should stop thinking about inhibition as a general construct likely depends on why the researcher is interested in it. Researchers who are interested in answering theoretical questions about the structure of executive functions (e.g., Friedman & Miyake, 2004) often administer multiple conflict tasks, use latent variable approaches to account for measurement error, and small but nonzero correlations can be theoretically meaningful. Research in this area is likely to continue, seeking improvements to task design and measurement (Draheim et al., 2020; Rey-Mermet, Gade, Souza, et al., 2019; Rouder et al., 2019; von Bastian et al., 2020). In contrast, some researchers use inhibition tasks as one of many tools to understanding individual differences in outcomes such as cognitive development (Carver et al., 2001; Dahlin, 2011), neuropsychological conditions (Hutton & Ettinger, 2006), or impulsivity (Skippen et al., 2019). Researchers in these contexts may use a single task, implicitly assuming it represents inhibition measures in general. For this assumption, *large* correlations between tasks are a prerequisite for interpreting any one task as a measure of general inhibitory ability. Our data, and the literature more widely, do not support such a generalization. Instead, researchers in these areas might be better served by focusing on tasks that are sensitive to the domain of interest (cf. Hutton & Ettinger, 2006; Rey-Mermet & Gade, 2018).

### Common Nonconflict Processes in Conflict Tasks

Our meta-analysis revealed consistent evidence for moderate to strong correlations in drift rate and boundary separation, which represent the efficiency of task-relevant processing and strategy/caution respectively. These parameters are notable because our simulations show that these nonconflict processes contribute substantially to individual differences in RT costs and error costs (see also Hedge et al., 2018; Hedge et al., 2018a; Miller & Ulrich, 2013). These findings also converge with evidence from factor analytic studies that performance in inhibition tasks can be (at least partly) accounted for by processing speed (Jewsbury et al., 2016; Rey-Mermet, Gade, Souza, et al., 2019) or goal maintenance and implementation (Friedman & Miyake, 2017; Kane & Engle, 2003). Overall, it appears that there are common mechanisms underlying performance in inhibition tasks, though they are not unique to conflict processing.

Our findings and approach contribute to the discussion in several ways. First, multiple studies have assumed that strategy may confound the measurement of individual differences and take steps to control for it (e.g., Draheim et al., 2016; Rey-Mermet, Gade, Souza, et al., 2019). However, they do not measure response caution and examine whether it correlates across tasks as we do here. Second, the finding that general processing speed is sufficient to account for individual differences in inhibition tasks in factor analytic studies is partly based on a failure to derive a unique inhibition factor (Karr et al., 2018; Rey-Mermet, Gade, Souza, et al., 2019). By using a model to dissociate and quantify the efficiency of controlled processing, captured by the drift rate parameter, we can provide positive evidence for common mechanisms.

Finally, although we draw parallels between the drift rate parameter and latent perceptual/processing speed factors identified in factor analytic studies (Hedden & Yoon, 2006; Jewsbury et al., 2016), it is not a given that they refer to the same underlying ability. A perceptual speed task might involve comparing the size of two letter strings to determine which is longest, with performance measured by the number completed in a fixed time limit (Hedden & Yoon, 2006). A latent variable—which might be called perceptual speed—is then derived from behavior across multiple tasks assumed to measure the same construct. In contrast, a cognitive model attempts to dissociate latent processes that contribute to behavior *within* a task. From an evidence accumulation model perspective, individual differences in this ‘perceptual speed’ factor could be driven by some combination of drift rate, boundary separation, and nondecision time. These two approaches to capturing latent psychological processes are not mutually exclusive, and some studies have used diffusion model parameters in a factor analysis in place of behavioral measures (e.g., Schmiedek et al., 2007). Such an integration may a useful approach to overcome the impurity of behavioral measures that we evidence here.

### Alternative Models

Our approach is only useful if the model employed is relevant to the way human brains process these tasks. All models make assumptions; we do not know the true model and the DMC may be a mischaracterization of the mechanisms of response control. We chose the framework of evidence accumulation models because they have previously offered valuable insights into individual differences in choice RT behavior (e.g., Hedge et al., 2018; Ratcliff et al., 2015). Further, we chose the DMC specifically because we needed a common framework for all tasks, whereas some alternative models invoke task specific mechanisms (White et al., 2011). Would we have reached different conclusions had we used a different evidence accumulation model, or a different family of models altogether?

It is common for evidence accumulation models to show a high degree of mimicry. Different models can often reproduce the same data patterns even though they make different assumptions (Donkin et al., 2011; Teodorescu & Usher, 2013). There are alternative sequential sampling models that have been applied to response control tasks, which involve extensions from standard diffusion or accumulator models (Bompas & Sumner, 2011; 2020; Bompas et al., 2017, Dillon et al., 2015; Hübner et al., 2010; Noorani & Carpenter, 2013; Weigard et al., 2019; White et al., 2011). Many of these extensions are designed to capture the observation that errors to incongruent stimuli are typically fast in tasks such as the flanker. They do this by assuming that there is a nonlinearity in the evidence accumulation process; information from the prepotent stimulus feature contributes more to the early period of the decision than it does to the late period. If we were to examine the evidence for common mechanisms in a different model, then we would inevitably look at correlations in the parameters responsible for this nonlinearity. We expect that this would lead to similar conclusions as we reach here because the challenge remains that these mechanisms contribute only in part to individual differences in behavior. In no commonly used accumulation model would behavioral congruency effects be unaffected by parameters representing strategy or overall processing speed (Hedge et al., 2018; Hedge et al., 2018a). Neither is this general point specific to evidence accumulation models (Miller & Ulrich, 2013; Pachella, 1974).

Outside of the accumulation model framework, different modeling approaches have been applied to conflict tasks. Perhaps most notable is the Stroop task, for which there are models based in a connectionist framework (e.g., Cohen et al., 1990), reinforcement learning (Verguts & Notebaert, 2009), and others (for a review, see Chuderski & Smolen, 2016). These models do not necessarily conflict with an evidence accumulation model account, and they sometimes share similar assumptions (Hübner et al., 2010; van Maanen & van Rijn, 2007). Here, we started with the working assumption that all tasks could be explained using a common framework. Instead, there may be value in using different models that are tailored to the assumptions underlying each task and examining correlations in conceptually related parameters across different models. For our current purposes, alternative models would still need to deal with the difficulty in distinguishing individual differences in conflict processing among the other processes that contribute to behavior.

An alternative model could possibly provide better quantitative fits to some of our data than the DMC does here. Indeed, our fits reveal some data patterns that may challenge the assumptions of the DMC (see Supplementary Material E in the online supplemental materials). In particular, in our implementation, the time-to-peak parameter couples the speed at which automatic activation peaks with the speed at which it is removed. This led to our fits erroneously predicting negative delta functions in data that had fast errors and slow RTs. It could be argued that this is an unfair test of the DMC, because it is designed as a model of two-choice behavior, and the data patterns that produced poorer fits were from four-choice tasks. The DMC reproduced the data patterns from our two-choice tasks well and was able to capture individual differences in all datasets to a degree. However, we are not the first to observe an underestimation of the conflict effect in slower RTs with the DMC (Hübner & Töbel, 2019). Notably, Hübner and Töbel also observed negative going delta functions in the flanker task when the onset of the flankers preceded the onset of the target. This suggests transient activation elicited by the conflicting stimulus feature is a plausible account of both the flanker and Simon tasks, though additional flexibility may be required to model it within a common framework.

We reiterate that our approach here is one of model application (Crüwell et al., 2019), and we are not testing the validity of the DMC. The primary motivation for developing the DMC was to demonstrate that positive and negative going delta functions can be understood within a common framework (Ulrich et al., 2015). The ability to capture individual differences is not a central assumption of the model, nor does the model assume that parameters should correlate across tasks.

### Alternative Perspectives on Response Control

To some theoretical perspectives, it may not be surprising that parameters derived from different tasks and modalities show weak correlations. Starting with Friedman and Miyake’s (2004; see also Miyake et al., 2000) influential work, many studies have used factor analysis to distinguish different subtypes of response control tasks (though earlier work had made conceptual distinctions (for example, Nigg, 2000). The three factors identified were inhibition of prepotent responses (antisaccade, Stroop, and stop-signal tasks), resistance to distractor interference (flanker, word naming, shape matching) and resistance to proactive interference (Brown-Peterson, AB-AC-AD, cued recall). It could be suggested that low correlations between some of our task pairs (for example, flanker, Simon) occur because they span different subfactors of this framework. However, this interpretation would not account for the low correlations we observe between more closely related task (Stroop, spatial Stroop), or the blocked and intermixed Simon task variants in Dataset 3.

We did not base our task selection on these previous taxonomies as they do not consistently replicate (Karr et al., 2018; Rey-Mermet et al., 2018). In recent revisions of their model of executive functioning, Friedman & Miyake (2017) have suggested that performance in inhibition tasks may be best explained by a more general construct, such as the ability to maintain and implement task goals. Recently, a large survey of the literature found that intertask correlations were not substantially larger within theoretical subgroups of tasks compared with between-subgroup pairs (von Bastian et al., 2020), so we do not expect to have observed different results had we used different tasks.

Beyond the individual differences context, Egner and colleagues (Egner, 2008; Egner et al., 2007) have suggested a dissociation between conflict arising from mismatched stimulus features (for example, the font color and the written word in the Stroop), and conflict arising through response mapping incompatibility (for example, stimulus location and response hand in the Simon). Egner et al. (2007) found in an fMRI study that stimulus-based and response-based conflict modulated activity in parietal and premotor cortex respectively. Thus, processing bottlenecks may occur at different stages of the complex brain pathways dealing with each task, but the overarching principles of conflict control may still be similar. Differences in stimulus properties, task relevance, and response modality may all modulate the weighted engagement of different underlying mechanisms (Bompas & Sumner, 2011; Bompas et al., 2017). Using models such as the DMC to decompose performance into underlying components might reveal common principles across tasks without necessitating common neural mechanisms.

Mechanisms of control go beyond reactively coping with conflict within a trial. For example, individuals adjust their behavior for following trials after experiencing conflict or errors (for example, Braem et al., 2014; Egner, 2008; Whitehead et al., 2019). Whitehead et al. (2019) found that the size of error-related slowing (Rabbitt, 1966) correlated across the flanker, Simon, and Stroop tasks, whereas the sequential congruency or Gratton effect (Gratton et al., 1992) did not. Further, the sequential congruency effect appears not to generally transfer from one type of conflict (for example, a Stroop stimulus) to another (for example, a flanker stimulus) when these different sources of conflict are intermixed (for reviews, see Braem et al., 2014; Egner, 2008; though there are exceptions, for example, Freitas et al., 2007). This represents converging evidence that there are task-specific mechanisms that process conflict, rather than shared.

### Summary and Conclusions

In Part I of this article, a meta-analysis showed no evidence for correlated conflict mechanisms, and robust evidence for correlations in strategy and processing speed across tasks. In Part II, our simulations show that correlations in traditional behavioral measures (RT costs and error costs) are not diagnostic of the source of common variance. Individual differences in strategy and processing speed can create or mask correlations in behavior depending on whether or not they are correlated themselves. Taken together, these findings show that drawing conclusions from individual differences in response control tasks, and, conversely, attempting to directly measure inhibition ability is a difficult task. This difficulty is an obstacle both to theory development, and to the study of neuropsychiatric disorders and socially problematic behaviors. We urge researchers to take into account individual differences in strategy and processing speed where possible, either at the task or analysis level.

## Supplementary Material

10.1037/xlm0001028.supp

## Figures and Tables

**Table 1 tbl1:** Summary of Datasets That Were Used for Modelling

Dataset	Source	Tasks	Neutral condition	*N*	Trials per condition
1	New data	Flanker Simon	Yes	50	336
2	Hedge et al. (2018)	Flanker Color-word Stroop	Yes	103	480
3	Hedge et al. (2018)	Simon (blocked trials) Simon (intermixed trials)	No	102	288
4	Hedge et al. (2019)	Flanker Color-word Stroop	Yes	43	576
5	Whitehead et al. (2019)	Flanker Color-word Stroop Spatial Stroop^a^	No	187	512
6	Whitehead et al. (2019)	Flanker Color-word Stroop Spatial Stroop^a^	No	203	256 Congruent 768 Incongruent
7	Whitehead et al. (2019)	Flanker Color-word Stroop Spatial Stroop^a^	No	213	360
*Note*. *N* refers to the number of participants retained after exclusions.					
^a^ The authors refer to this as a Simon task, noting that it can also be thought of as a spatial Stroop. We refer to it as a spatial Stroop to distinguish it from the format of the Simon task in Datasets 1 and 3. See Supplementary Material A in the online supplemental materials for details.					

**Table 2 tbl2:** Parameters Used for Model Simulations

Parameter	*M*	*SD*
Amplitude of activation (A)	27.5	4.17
Time-to peak of activation (tau)	72	16.67
	135	
	505	
Upper boundary (b)	62.5	5.83
Nondecision time (Ter)	335	21.67
Drift rate (μc)	0.5	0.1
Starting point shape (a)	2.5	0.167
Nondecision time variability (TerSD)	0	0
*Note*. *M* and *SD* refer to the population values used to generate parameters for simulations, based on the ranges reported in Table A1 (Appendix A) and White et al. (2018). The three *M* time-to-peak values correspond to separate simulations designed to represent the Simon, flanker, and Stroop tasks.					

**Table A1 tbl3:** Parameter Values Used in Model Fitting and Simulations, Based on White et al. (2018)

Parameter	Minimum	Maximum
Amplitude of activation (A)	15	40
Time-to peak of activation (tau)	20 (100)	120 (600)
Upper boundary (b)	45	80
Nondecision time (Ter)	270	400 (500)
Drift rate (μc)	0.2	0.8
Starting point shape (a)	1	3 (10)
Nondecision time variability (TerSD)	20	50
*Note*. Minimum and maximum refer to the edges of a uniform distribution used to generate parameters for our initial fitting. The same ranges were used for all datasets except where values given in parentheses were used instead (for the four-choice and Stroop tasks). White et al. report the boundary separation (upper boundary × 2). We fix the shape parameters of the automatic activation to two. The diffusion constant (within-trial noise) was fixed to four.										

**Table B1 tbl4:** Parameter Recovery Correlations (Pearson’s r) for the Diffusion Model for Conflict Tasks

Dataset	Task	Amplitude	Time-to-peak	Drift rate	Boundary separation	Nondecision time	Starting point variability	Nondecision variability
1	Flanker	0.90	0.48	0.92	0.94	0.99	0.81	0.88
	Simon	0.86	0.86	0.96	0.94	0.98	0.78	0.94
2	Flanker	0.93	0.53	0.91	0.96	0.98	0.69	0.96
	Stroop	0.84	0.01	0.94	0.94	0.97	0.62	0.98
3	Simon intermixed	0.79	0.86	0.93	0.92	0.92	0.64	0.84
	Simon blocked (cong.)	0.70	0.42	0.81	0.62	0.91	0.66	0.88
	Simon blocked (incong.)				0.88			
4	Flanker (Standard)	0.88	0.48	0.86	0.97	0.98	0.70	0.98
	Flanker (Speed)				0.97			
	Flanker (Accuracy)				0.94			
	Stroop (Standard)	0.59	0.17	0.95	0.99	0.95	0.37	0.97
	Stroop (Speed)				0.98			
	Stroop (Accuracy)				0.98			
5	Flanker	0.58	0.55	0.93	0.95	0.98	0.62	0.90
	Spatial Stroop	0.93	0.81	0.96	0.93	0.99	0.63	0.92
	Stroop	0.85	0.33	0.90	0.90	0.94	0.46	0.83
6	Flanker	0.67	0.11	0.95	0.94	0.88	0.58	0.87
	Spatial Stroop	0.95	0.67	0.97	0.96	0.96	0.53	0.90
	Stroop	0.80	0.20	0.91	0.92	0.92	0.47	0.94
7	Flanker	0.56	0.38	0.87	0.91	0.89	0.43	0.89
	Spatial Stroop	0.95	0.84	0.95	0.94	0.98	0.58	0.92
	Stroop	0.84	−0.08	0.81	0.81	0.95	0.67	0.94
	Median	0.84	0.48	0.93	0.94	0.96	0.62	0.92
	Minimum	0.56	−0.08	0.81	0.62	0.88	0.37	0.83
	Maximum	0.95	0.86	0.97	0.99	0.99	0.81	0.98
*Note*. Data were simulated from the best fitting parameters to our empirical datasets, and simulated data were subsequently fit using the same pipeline as our main analysis (see Appendix A).										

**Table C1 tbl5:** Means and Standard Deviations for Best Fitting Model Parameters to Empirical Datasets

Dataset	Task	Boundary	Nondecision	Amplitude	Drift rate	Time to peak	Start shape	Nondecision variability
1	Flanker	55.6 (10)	334 (27)	31.5 (9)	.76 (.13)	113 (23)	2.1 (0.8)	34 (8)
	Simon	51.2 (12)	302 (23)	18.4 (4.9)	.60 (.13)	72 (36)	2.4 (0.8)	38 (9)
2	Flanker	53.5 (10.2)	343 (31)	23.8 (8)	.65 (.15)	135 (24)	1.9 (0.8)	46 (15)
	Stroop	71.7 (11.5)	435 (63)	19.3 (9.4)	.33 (.07)	501 (192)	6.5 (2.9)	92 (38)
3	Simon Mix	59.2 (11.8)	310 (22)	14 (5.7)	.50 (.12)	69 (42)	2.1 (0.9)	42 (12)
	Simon Cong.	48.3 (9.4)	264 (19)	22.9 (8.5)	.87 (.21)	108 (31)	2 (0.7)	40 (9)
	Simon Incong.	60.4 (13.8)						
4	Flanker Spd.	28.7 (10.7)						
	Flanker Std.	45.5 (12.4)	312 (22)	24 (7.3)	.68 (.13)	129 (40)	1.8 (0.7)	49 (8)
	Flanker Acc.	59.5 (11.5)						
	Stroop Spd.	27.3 (13.9)						
	Stroop Std.	58.7 (13.8)	384 (29)	21 (7.6)	.28 (.06)	634 (227)	8.3 (4.3)	82 (18)
	Stroop Acc.	67.4 (13.7)						
5	Flanker	85.5 (24.1)	461 (85)	10.8 (5.8)	.42 (.13)	100 (56)	2.8 (0.8)	72 (37)
	Spatial Stroop	78 (12.5)	413 (51)	26.1 (8)	.50 (.13)	87 (27)	2.6 (0.6)	58 (18)
	Stroop	84.1 (16.5)	446 (76)	18 (10.1)	.29 (.07)	538 (207)	6 (3)	83 (50)
6	Flanker	84.3 (22)	468 (48)	7.4 (4.7)	.40 (.12)	99 (50)	3.1 (0.9)	74 (36)
	Spatial Stroop	78.2 (13.8)	427 (33)	20.7 (7.3)	.48 (.14)	105 (29)	2.8 (0.5)	52 (15)
	Stroop	87 (17.7)	427 (59)	16.1 (8.4)	.28 (.07)	548 (194)	6.5 (3.2)	85 (50)
7	Flanker	95.2 (28.8)	474 (70)	12.2 (5.8)	.41 (.11)	101 (48)	2.7 (0.7)	81 (59)
	Spatial Stroop	84.1 (13.6)	416 (35)	32.2 (9.8)	.48 (.11)	122 (33)	2.7 (0.6)	51 (17)
	Stroop	94.3 (17.4)	447 (92)	22.8 (10.1)	.30 (.08)	495 (200)	5.2 (2.7)	93 (75)
*Note*. Multiple boundary separation values are given for tasks in which the parameter could vary between conditions. All other parameters were constrained across conditions.										

**Table C2 tbl6:** Pearson Correlations Between Observed Accuracy and Accuracy in Data Simulated From Best Fitting Model Parameters for Each Individual

Dataset	Task	Congruent	Neutral	Incongruent
1	Flanker	0.94	0.87	0.97
	Simon	0.95	0.96	0.98
2	Flanker	0.96	0.95	0.96
	Color word Stroop	0.93	0.94	0.94
3	Simon blocked	0.82		0.95
	Simon intermixed	0.92		0.95
4	Flanker Speed	0.94	0.91	0.88
	Flanker Accuracy	0.87	0.73	0.91
	Flanker Standard	0.96	0.91	0.93
	Stroop Speed	0.94	0.93	0.95
	Stroop Accuracy	1	0.99	0.99
	Stroop Standard	0.99	0.99	0.99
5	Spatial Stroop	0.92		0.99
	Color word Stroop	0.92		0.92
	Flanker	0.84		0.96
6	Spatial Stroop	0.84		0.99
	Color word Stroop	0.73		0.9
	Flanker	0.85		0.95
7	Spatial Stroop	0.94		0.97
	Color word Stroop	0.79		0.87
	Flanker	0.78		0.89
*Note*. Correlations ranged from .73 to 1 (*M* = .92).										

**Table C3 tbl7:** Pearson Correlations Between Percentiles of Correct Reaction Times in Data Simulated From Best Fitting Model Parameters for Each Individual

		25th Percentile			50th Percentile			75th Percentile		
Dataset	Task	Congruent	Neutral	Incongruent	Congruent	Neutral	Incongruent	Congruent	Neutral	Incongruent
1	Flanker	0.98	0.96	0.96	0.99	0.97	0.98	0.98	0.97	0.98
	Simon	0.98	0.98	0.98	0.99	0.99	0.98	0.99	0.99	0.99
2	Flanker	0.96	0.97	0.96	0.98	0.98	0.97	0.99	0.97	0.98
	Color word Stroop	0.96	0.97	0.96	0.97	0.98	0.97	0.98	0.97	0.96
3	Simon blocked	0.95		0.96	0.97		0.98	0.97		0.98
	Simon intermixed	0.97		0.96	0.98		0.99	0.98		0.98
4	Flanker Speed	0.97	0.97	0.96	0.96	0.96	0.97	0.96	0.94	0.96
	Flanker Accuracy	0.96	0.94	0.95	0.98	0.96	0.99	0.98	0.97	0.99
	Flanker Standard	0.96	0.97	0.97	0.98	0.98	0.98	0.98	0.98	0.98
	Stroop Speed	0.95	0.96	0.94	0.94	0.98	0.96	0.95	0.98	0.96
	Stroop Accuracy	0.99	0.99	0.99	0.99	1	0.99	0.99	0.99	0.99
	Stroop Standard	0.99	0.99	0.99	0.99	1	1	0.99	0.99	0.99
5	Spatial Stroop	0.99		0.98	1		0.99	0.99		0.99
	Color word Stroop	0.97		0.97	0.97		0.97	0.96		0.96
	Flanker	0.99		0.99	0.99		0.99	0.99		0.99
6	Spatial Stroop	0.94		0.97	0.95		0.98	0.96		0.99
	Color word Stroop	0.85		0.91	0.89		0.96	0.88		0.95
	Flanker	0.95		0.98	0.98		0.99	0.97		0.99
7	Spatial Stroop	0.96		0.92	0.99		0.96	0.98		0.98
	Color word Stroop	0.95		0.95	0.94		0.95	0.93		0.94
	Flanker	0.96		0.97	0.98		0.98	0.98		0.98
*Note*. Correlations ranged from .85 to 1 (*M* = .97).										

**Table C4 tbl8:** Pearson Correlations Between Percentiles of Incorrect Reaction Times in Data Simulated From Best Fitting Model Parameters for Each Individual

		25th Percentile			50th Percentile			75th Percentile		
Dataset	Task	Congruent	Neutral	Incongruent	Congruent	Neutral	Incongruent	Congruent	Neutral	Incongruent
1	Flanker	0.71	0.72	0.84	0.72	0.74	0.84	0.66	0.75	0.87
	Simon	0.71	0.59	0.88	0.83	0.86	0.87	0.78	0.8	0.75
2	Flanker	0.75	0.79	0.89	0.71	0.76	0.91	0.67	0.75	0.9
	Color word Stroop	0.81	0.84	0.77	0.83	0.81	0.8	0.79	0.79	0.78
3	Simon blocked	0.55		0.7	0.56		0.73	0.56		0.73
	Simon intermixed	0.77		0.83	0.83		0.87	0.81		0.87
4	Flanker Speed	0.82	0.87	0.92	0.83	0.89	0.91	0.86	0.84	0.87
	Flanker Accuracy	0.71	0.7	0.81	0.66	0.71	0.89	0.7	0.67	0.89
	Flanker Standard	0.83	0.87	0.91	0.82	0.91	0.93	0.78	0.92	0.9
	Stroop Speed	0.89	0.91	0.91	0.9	0.91	0.92	0.96	0.95	0.93
	Stroop Accuracy	0.96	0.97	0.96	0.94	0.97	0.96	0.91	0.96	0.95
	Stroop Standard	0.97	0.97	0.95	0.98	0.96	0.95	0.97	0.96	0.94
5	Spatial Stroop	0.62		0.86	0.67		0.89	0.68		0.87
	Color word Stroop	0.73		0.69	0.66		0.68	0.65		0.68
	Flanker	0.83		0.82	0.82		0.79	0.79		0.78
6	Spatial Stroop	0.34		0.8	0.42		0.85	0.56		0.79
	Color word Stroop	0.62		0.8	0.57		0.71	0.53		0.65
	Flanker	0.57		0.81	0.63		0.77	0.7		0.75
7	Spatial Stroop	0.42		0.9	0.51		0.85	0.55		0.76
	Color word Stroop	0.56		0.7	0.67		0.65	0.68		0.61
	Flanker	0.71		0.7	0.72		0.68	0.72		0.68
*Note*. Correlations ranged from .34 to .98 (*M* = .78). Correlations are expected to be lower for incorrect RTs because they are based on fewer data points.										

## Footnotes

1 Note that the time at which the peak amplitude is reached is only equal to the scale parameter when the shape parameter is fixed to 2 (Ulrich et al., 2015), which was our case. It is defined by: *tamx* = *tau* × (shape−1)

## A Model Fitting Details

To fit the DMC to experimental data, we adapted the approach of White et al. (2018). We estimated seven parameters of the DMC separately for each participant in each task. The parameters representing conflict processing were the amplitude of automatic activation (A for congruent trials, −A for incongruent trials), and the time to peak automatic activation (tau). The nonconflict decision parameters are boundary separation (b), drift rate of the controlled process (µc), and the shape parameter of the beta distribution used to represent starting points of the accumulation process (α). Finally, nondecision time is implemented as a Gaussian distribution with parameters for the mean (Ter) and variability (TerSD). In Datasets 3 and 4, we estimated additional boundary separation parameters to capture the experimental manipulations. In Dataset 4, we estimated three separate boundary separation values to capture strategic differences between blocks in which we emphasized either speed, accuracy, or both speed and accuracy. We calculated the between-task correlation in boundary separation under each instruction condition and entered all three into our meta-analysis. In Dataset 3 (intermixed versus blocked Simon task), we derived separate boundary separation estimates for congruent-only and incongruent-only blocks. As our mixed-trial Simon variant produced a single boundary separation estimate, we averaged the two values from the blocked variant to obtain a single correlation for this parameter.

For Datasets 1, 2, and 4, we also had data from a neutral condition, which we included in the fitting with the amplitude of the automatic activation fixed to zero. For each participant within each task only the amplitude parameter provides the difference between congruent, neutral, and incongruent trials; all other parameters were constrained to be equal across conditions. As with Ulrich et al. (2015), the diffusion constant/within-trial noise (σ) was fixed to 4. We fixed the shape parameter of the automatic activation function to 2 for all tasks, following Ulrich et al. (2015).

We accuracy-coded our data, so that the upper and lower response boundaries correspond to thresholds for correct and incorrect responses, respectively. Note that the DMC is a model of a two-choice task, whereas some of our datasets contained four-choice tasks. Multichoice tasks can be accommodated by accuracy coding, which, although not ideal, allowed us to interpret all the datasets within a common framework. Correct and incorrect RTs from congruent, neutral (where available), and incongruent conditions were separately binned into quantiles. Correct RTs were binned into five quantiles (.1, .3, .5, .7, .9) for each condition separately. The same approach was applied for incorrect RTs in each condition when the total number of errors in that condition ≥ 10. When between five and 10 errors were made, three quantiles were used (.3, .5, .9) for incorrect RTs. If fewer than five errors were made, we fit the median RT of the errors. We calculated the deviance (−2 log-likelihood) between observed and simulated quantiles, which was minimized with a Nelder-Mead simplex (Nelder & Mead, 1965) implemented in the fminbnd function in Matlab. We constrained the search such that all free parameters were positive, and the shape of the starting point distribution was greater than one.

We first fit the data using 5000 parameter sets generated from a uniform distribution within the minimum and maximum values given in Table A1 (based on White et al., 2018), with simulations consisting of 5,000 trials per condition. We then took the 15 best parameter sets resulting from this initial search, and submitted each of those to the simplex algorithm, in which we simulated 10,000 trials per condition at each iteration. The simplex was reinitialized 3 times to avoid local minima. After the process was completed, we took the single best fitting parameter set for each individual. This process took approximately 30–40 hr per individual per task and was performed on Cardiff University Brain Research Imaging Centre’s (CUBRIC) high performance computer cluster.[Table tbl3]

At the time of fitting, we were the first to apply the DMC to a Stroop task (though see Ambrosi et al., 2019 for a recent analysis with child data), and we noticed during preliminary examination of our data that our fitting routine would typically converge to values outside our initial search space for the nondecision time, time-to-peak, and shape of the starting distribution parameters. Unlike the flanker and Simon tasks, participants did not make fast errors in our Stroop task (see Appendix C; see also Figure 3 in Vandenbossche et al., 2012, for a similar pattern of errors in the Stroop task.). To aid parameter optimization, we refit the Stroop data using a higher range of starting parameters, noted in Table A1. It is plausible that interference in the Stroop task has a later time course compared with the flanker task or Simon task, since semantic word processing is expected to be slower than processing of location or simple visual symbols. This is supported by evidence from event-related potentials (ERPs). In a study that combined flanker and Stroop stimuli, ERPs for congruent and incongruent stimuli diverged earlier for flanker conflict than for Stroop conflict (Rey-Mermet, Gade, & Steinhauser, 2019; see also Kałamała et al., 2018; Liotti et al., 2000). We also used the higher range of nondecision time when fitting Datasets 5 to 7, as these datasets typically had slower RTs.

## B Parameter Recovery

A parameter recovery exercise tells us whether the model and our fitting procedure can consistently identify different levels of a parameter in data. For example, if data are generated with a relatively high amplitude of automatic activation then we want our fitting to return a relatively high value. We simulated data from the best fitting parameters for each task and individual in a dataset, with the number of trials corresponding to what was originally administered in the task. We then fit the simulated data using the same procedure that we used on the empirical data and correlated the best fitting parameters with those used to generate the data. These correlations (Pearson’s *r*) are shown in Table B1.[Table tbl4]

The amplitude parameter was recovered well for most tasks and datasets (median *r =* .84, range .56 to .95). Recovery of the time-to-peak parameter was relatively poor (median *r =* .48, range −.08 to .86). Recovery of the drift rate, boundary separation, mean nondecision time, and nondecision time variability parameters was good (median *r* ≥ .90 for all). Starting point variability could also be recovered to a lesser extent (median *r* = .62). The poor recovery for the time-to-peak parameter contrasts to the good recovery reported by White et al. (2018), using a similar approach. We suspect that the reason for this is that the time-to-peak values produced in our empirical fits of exceeded the maximum of the ranges used by White et al. (20–120), particularly in the flanker and Stroop tasks (see Appendix C). For example, the mean time-to-peak values range across datasets from 99 to 135 for the flanker tasks and 495 to 634 for the Stroop tasks. It is possible that the time-to-peak parameter is not uniquely identifiable in tasks/ranges that do not produce negative going delta functions.

In the main text, we reported the results of a sensitivity power analysis that showed that our meta-analysis had 80% power to detect an average correlation of *r =* .07 in the presence of low heterogeneity (which we observe in the conflict parameters). These parameter recovery simulations do not change the size of correlation that we can detect in the data. However, if we assume that this observable correlation is attenuated due to less-than-perfect parameter recovery, in the same way than unreliability attenuates correlations, then we can calculate the corresponding true correlation using Spearman’s (1904) diattenuation formula below. For illustration, we apply this formula assuming a worst-case scenario for the amplitude parameter, where we assume all tasks in all datasets had recovery equal to the worst that we observed for any task (*r =* .56). Note that for most tasks and datasets it was much higher.

$$True\;r\left(x,y\right)=\frac{\text{Observed r}\left(\text{x},\text{y}\right)}{\sqrt{\text{Reliability}\left(\text{x}\right)\cdot \text{Reliability}\left(\text{y}\right)}}\;=0.13\;=\frac{0.07}{\sqrt{0.56.0.56}}\;$$

The demonstrates that a correlation of *r =* .07 in the data corresponds to an estimated true correlation of *r =* .13, which is on the lower end of what is traditionally considered to be a small effect size (*r =* .1; Cohen, 1988). In other words, our parameter recovery is sufficiently sensitive for our current purposes.

## C Descriptive Statistics for Model Parameters and Model Fits

Here we report the means and standard deviations for the best fitting model parameters in our empirical fits (Table C1). As an indication of model fits, we also report the Pearson correlations between the empirical data and data simulated from the best fitting parameters (Tables C2–C4). Scatter plots for these fits are shown in Supplementary Material F, along with empirical and simulated conditional accuracy and delta functions.[Table tbl5][Table tbl6][Table tbl7][Table tbl8]

The high positive correlations show that the model fits capture the rank order of participants in all task/datasets. Correlations are lower for incorrect reaction times (Table C4), which are based on fewer data points. However, note from the plots in Supplementary Material F that model fits tended to underestimate the speed of slower reaction times in some tasks and datasets (Datasets 5 to 7; also see Figure 4 in the main text). link tables, figures and Supplementary Material F.
