# Supplementary material for: Strategy and Processing Speed Eclipse Individual Differences in Control Ability in Conflict Tasks
Source: J Exp Psychol Learn Mem Cogn. 2021 Sep 30;48(10):1448–69. doi: 10.1037/xlm0001028 (PMC9899369; doi:10.1037/xlm0001028)

**Supplementary Material A: Detailed methods**

Here we report the detailed methods for dataset 1, which have not previously been published. We give a brief summary for the other datasets. For detailed information on dataset 2, we refer to the method section of Hedge et al., (2018). For dataset 3, we refer to the method section of Hedge et al. (2018). For dataset 4, see the method section of (Hedge, Vivian-Griffiths, Powell, Bompas, & Sumner, 2019). For datasets 5 to 7, see Whitehead et al. (2018).

**Method.** Participants completed both flanker and Simon tasks in a single hour-long session. After completing both behavioural tasks, participants completed the UPPS-P (self-reported impulsivity). Participants alternated between blocks of each task throughout the session, with the starting task counterbalanced across participants. Prior to testing, participants performed a practice block of 24 trials for each task. There were seven test blocks of 144 trials for each task, divided equally between congruent, neutral and incongruent conditions. Participants therefore completed 336 trials in each condition in each task. A schematic of the tasks used in all datasets is shown in Figure 3.

***Participants.*** Participants were 50 (12 male) undergraduate students aged 18-28 (M=20.06, SD=2.24). All participants gave their informed written consent prior to participation in accordance with the revised Declarations of Helsinki (2013), and the experiments were approved by the local Ethics Committee.

***Eriksen flanker task.*** Participants responded to the direction of a centrally presented arrow (left or right) using the \ and / keys. On each trial, the centrally presented arrow (1cm x 1cm) was flanked above and below by two other symbols separated by 0.75cm. Flanking stimuli were either arrows pointing in the same direction as the central arrow (congruent condition), straight lines (neutral condition), or arrows pointing in the opposite direction to the central arrow (congruent condition). Stimuli were presented until a response was given, with an Inter-Stimulus Interval (ISI) of 750ms.

***Simon task.*** Participants responded to the colour (green or blue) of a circle (2cm in diameter) using the \ and / keys. On each trial, the circles were presented either centrally (neutral condition) or 4cm to the left or right of centre. Trials on which the circle was presented on the same side as the required response hand were congruent, and those on which it was presented on the opposite side were incongruent. Stimuli were presented until a response was given, with an Inter-Stimulus Interval (ISI) of 750ms.

**Dataset 2: Flanker and Stroop.** These data were originally collected to assess the test-retest reliability of response control tasks (Hedge, Powell, & Sumner, 2018b). In two studies with identical procedures, participants (combined N = 103) completed four tasks (flanker, Stroop, stop-signal and go/no-go). We do not model the stop-signal and go/no-go tasks here as they are not suited to the DMC framework. The flanker task was as described in Dataset 1. We used a four choice Stroop task, in which participants responded to the font colour (red, blue, green, yellow) of a centrally presented word. The word could either match the font colour (congruent), refer to a different colour used in the response set (incongruent), or be a non-colour word. Participants completed all the tasks in two sessions, three weeks apart. We combine the data from both sessions and both studies for the analyses reported here. Full details can be seen in Hedge et al. (2018b).

**Dataset 3: Intermixed and blocked Simon task.** Dataset 3 was previously collected to test the prediction that the correlation between RT costs and error costs would be more positive when congruent and incongruent trials were randomly intermixed compared to separate blocks of congruent and incongruent trials (Hedge, Powell, Bompas, et al., 2018). In a single session**,** participants (N=102) completed blocks consisting of congruent trials only (two blocks), incongruent trials only (two blocks), and both congruent and incongruent intermixed (four blocks). A mixed-trial block always occurred between congruent only and incongruent only blocks, with the starting block counterbalanced across participants.

**Dataset 4: Flanker and Stroop.** Dataset 4 was collected to examine the test retest reliability of the speed-accuracy trade-off induced by instructing participants to emphasise either speed, accuracy or both speed and accuracy (Hedge et al., 2019). Participants completed the flanker and Stroop tasks as described in datasets 1 and 2, in two sessions separated by four weeks. For each session and tasks, participants completed 4 blocks each for speed, standard (both speed and accuracy) and accuracy conditions. Participants were shown instructions at the beginning of each block to emphasise the relevant performance dimension. We combine the data from both sessions for the analyses here.

**Datasets 5 to 7: Flanker, Stroop and spatial Stroop.** Datasets 5 to 7 are three experiments taken from Whitehead et al. (2019). All three experiments were designed to test whether individual differences in the sequential congruency effect (SCE) and error-related slowing (ERS) correlate across tasks.

All tasks used by Whitehead et al. (2019) were four choice tasks. In their flanker task, participants responded to the identity of a letter (D, F, J, or K) while ignoring flanking letters that could be either congruent or incongruent. The Stroop task was as described for dataset 2. In the spatial Stroop task, participants responded to the identity of a directional word (right, left, up or down) which could be presented in a congruent location (e.g. “up” above central fixation) or an incongruent location (e.g. “below” to the right of central fixation). The datasets differed in the ratio of congruent to incongruent trials (Datasets 5 and 7 were 50/50, Dataset 6 consisted of a 25/75 congruent), and Datasets 6 and 7 allowed for feature-repetitions and both feature-repetitions and target-distractor contingencies respectively. The manipulations were designed to modulate the size of conflict adaptation effects and are not directly relevant to our goal here. Participants completed eight blocks of 128 trials (Datasets 5 and 6) or six blocks of 120 trials (Dataset 7), of which the first two or one respectively were considered practice blocks. In Dataset 7 and the practice blocks of Dataset 6, trials that produced an error or an RT >3000ms were repeated at the end of a block. We include all trials in our analysis here as we previously did not observe discernible practice effects in comparable tasks, but we did observe a benefit to having more trials overall (see Supplementary Material D of Hedge, Powell & Sumner, 2018b).

**Supplementary Material B: The impact of alternative analysis choices on the meta-analysis**

**Stricter accuracy cut-off**

In the analysis in the main text, our inclusion criteria was that individual’s average accuracy across all conditions was at least 60%. In order to verify that our findings were not dependent on the inclusion of participants who were not fully attentive, we rerun the correlations reported in the main text a stricter cut-off of 80%. We are looking at whether we observe correlations in conflict parameters which we did not observe in the main analyses. The results did not noticeably differ from those in the main text (see Figure B1). Correlations in conflict parameters still do not significantly differ from zero, and we still consistently see moderate to strong correlations in drift rate, boundary separation and non-decision time.

*Figure B1. Meta-analytic (black diamonds) and observed (circles) correlations between tasks in parameters of the diffusion model for conflict tasks (DMC) with stricter accuracy cut-offs (>80% correct compared to >60% in main analysis). Error bars show 95% confidence intervals. A multi-level random effects meta-analysis was performed on Spearman’s rho correlations calculated for each pair of tasks, allowing for clustering where multiple correlations were taken from the same dataset. The amplitude and Time to peak parameters are those that capture conflict processing.*

*Table B1.*

*Meta-analysis of model parameters with a stricter accuracy cut-off (>80%)*

| Parameter | Rho | Lower CI | Upper CI | I2 | p |
| --- | --- | --- | --- | --- | --- |
| Amplitude | 0.05 | -0.01 | 0.11 | 20.70 | 0.11 |
| Time to peak | 0.04 | -0.02 | 0.09 | 27.04 | 0.18 |
| Drift rate | 0.32 | 0.28 | 0.37 | 4.04 | 0.00 |
| Boundary separation | 0.56 | 0.50 | 0.61 | 47.22 | 0.00 |
| Non-decision time | 0.58 | 0.49 | 0.68 | 81.13 | 0.00 |
| Starting point variability | 0.14 | 0.02 | 0.26 | 80.91 | 0.02 |
| Non-decision variability | 0.26 | 0.19 | 0.33 | 61.75 | 0.00 |

**Including task pair as a factor**

Our datasets included repetitions of certain task pairs. For example, there were multiple correlations between a flanker and colour word Stroop task. In total there were five combinations, with the number of data points per pair given in parentheses:

- Flanker & Simon (1)
- Flanker & colour word Stroop (5)
- Simon blocked & Simon intermixed (1)
- Flanker & Spatial Stroop (3)
- Spatial Stroop & colour word Stroop (3)

It is plausible that some tasks share more in common than others, leading to variance in the effect sizes that can be attributed to task pairing. Due to the limited number of data points (only one data point for two of the pairs), we did not include this additional complexity in our main analysis. We report the results of an analysis that includes this additional level below. As in our main analysis, we see small and non-significant average correlations in the conflict parameters, and moderate to large correlations in the non-conflict parameters. Further, the variance account for by task pair is low (<25%). Thus, though we acknowledge the limitations of this analysis due to the limited data, we find no evidence of heterogeneity in the conflict parameters associated task pairs.

*Table B2.*

*Meta-analysis of model parameters including task pair variability*

| **Parameter** | **rho** | **LowerCI** | **Upper**  **CI** | **I2 dataset** | **I2 Dataset/Effect** | **I2 Task Pair** | **p** |
| --- | --- | --- | --- | --- | --- | --- | --- |
| Amplitude | 0.04 | -0.01 | 0.1 | 18.54 | 0 | 0 | 0.13 |
| Time to peak | 0.03 | -0.03 | 0.09 | 0 | 5.74 | 17.45 | 0.29 |
| Drift rate | 0.32 | 0.26 | 0.39 | 30.09 | 0 | 6.18 | 0 |
| Boundary separation | 0.54 | 0.48 | 0.6 | 46.84 | 0 | 5.68 | 0 |
| Non-decision time | 0.58 | 0.46 | 0.7 | 26.35 | 2.42 | 57.22 | 0 |
| Starting point variability | 0.22 | 0.07 | 0.38 | 0 | 5.69 | 76.86 | 0 |
| Non-decision variability | 0.29 | 0.2 | 0.39 | 27.75 | 0 | 37.35 | 0 |

**Supplementary Material C: Descriptive statistics and reliability for empirical data**

Table C1. Means and standard deviations (parentheses) for error rates and reaction times.

|  |  | Errors (%) | | | Reaction times (ms) | | |
| --- | --- | --- | --- | --- | --- | --- | --- |
| Dataset | Task | Congruent | Neutral | Incongruent | Congruent | Neutral | Incongruent |
| 1 | Flanker | 2.6 (2.4) | 4 (3.2) | 12.5 (6.9) | 391 (33) | 391 (33) | 443 (38) |
|  | Simon | 6.1 (3.7) | 7 (5.1) | 13.1 (7.8) | 366 (37) | 380 (38) | 400 (39) |
| 2 | Flanker | 5.8 (5.6) | 6.8 (5.7 | 14.9 (8.3) | 413 (42) | 408 (40) | 454 (51) |
|  | Stroop | 6.6 (3.5) | 8 (4.1) | 9.6 (4.9) | 597 (65) | 626 (71) | 670 (77) |
| 3 | Simon Block | 3.2 (2.8) |  | 6.8 (5.9) | 308 (26) |  | 354 (40) |
|  | Simon Mix | 7.9 (4.4) |  | 11.2 (5.9) | 408 (47) |  | 429 (43) |
| 4. Speed | Flanker | 13.1 (6.7) | 15.2 (7.1) | 28.7 (8.5) | 345 (27) | 341 (26) | 366 (37) |
|  | Stroop | 28.6 (10.6) | 31.9 (9.2) | 36.1 (8.8) | 434 (38) | 446 (44) | 450 (49) |
| 4. Standard | Flanker | 6.7 (5) | 8 (5.2) | 18.8 (7) | 371 (35) | 366 (33) | 405 (49) |
|  | Stroop | 12.2 (6.7) | 14.9 (6.4) | 17.5 (6.5) | 514 (50) | 543 (58) | 573 (78) |
| 4. Accuracy | Flanker | 2.9 (2.3) | 3.9 (2.4) | 11.6 (5.8) | 390 (35) | 384 (33) | 430 (45) |
|  | Stroop | 8.1 (5) | 10 (5.7) | 11.8 (6.1) | 540 (55) | 569 (64) | 609 (78) |
| 5 | Flanker | 4.7 (3.9) |  | 6.4 (5.8) | 641 (129) |  | 682 (137) |
|  | Spatial Stroop | 2.4 (2.6) |  | 10.1 (7.6) | 528 (74) |  | 596 (78) |
|  | Stroop | 7.3 (7.1) |  | 9.7 (7.8) | 662 (109) |  | 749 (139) |
| 6 | Flanker | 5.5 (4.1) |  | 6.7 (4.6) | 659 (95) |  | 687 (95) |
|  | Spatial Stroop | 2.6 (2.8) |  | 8.4 (5.7) | 547 (56) |  | 615 (64) |
|  | Stroop | 7.5 (6.7) |  | 9.7 (7.7) | 660 (92) |  | 735 (112) |
| 7 | Flanker | 4.4 (3.9) |  | 5.5 (4.8) | 673 (117) |  | 731 (126) |
|  | Spatial Stroop | 2 (4.1) |  | 9.6 (7) | 531 (57) |  | 635 (62) |
|  | Stroop | 4.6 (4.3) |  | 8.2 (7) | 680 (105) |  | 809 (132) |

Table C2. Split-half reliability for behavioural measures in empirical data. Reliability coefficients are calculated on odd vs. even trials, using the formula for the agreement of the average rater (ICC[2,k]).

|  | Task | Average Accuracy | Average RT | Error cost | RT cost |
| --- | --- | --- | --- | --- | --- |
| 1 | Flanker | 0.96 | 0.99 | 0.89 | 0.88 |
|  | Simon | 0.98 | 0.99 | 0.82 | 0.74 |
| 2 | Flanker | 0.99 | 1.00 | 0.84 | 0.89 |
|  | Stroop | 0.97 | 0.99 | 0.67 | 0.89 |
| 3 | Simon Block | 0.95 | 0.98 | 0.89 | 0.91 |
|  | Simon Mix | 0.95 | 0.97 | 0.83 | 0.60 |
| 4 | Flanker Speed | 0.96 | 0.99 | 0.82 | 0.83 |
|  | Stroop Speed | 0.99 | 0.99 | 0.49 | 0.49 |
|  | Flanker Standard | 0.97 | 0.99 | 0.84 | 0.93 |
|  | Stroop Standard | 0.99 | 0.99 | 0.65 | 0.75 |
|  | Flanker Accuracy | 0.95 | 1.00 | 0.85 | 0.89 |
|  | Stroop Accuracy | 0.98 | 0.99 | 0.34 | 0.72 |
| 5 | Flanker | 0.86 | 0.89 | 0.88 | 0.46 |
|  | Spatial Stroop | 0.86 | 0.89 | 0.78 | 0.67 |
|  | Stroop | 0.10 | 0.72 | 0.15 | 0.11 |
| 6 | Flanker | 0.97 | 0.98 | 0.29 | 0.42 |
|  | Spatial Stroop | 0.98 | 0.97 | 0.90 | 0.72 |
|  | Stroop | 0.57 | 0.75 | 0.11 | -0.02 |
| 7 | Flanker | 0.97 | 0.99 | 0.65 | 0.63 |
|  | Spatial Stroop | 0.98 | 0.99 | 0.94 | 0.80 |
|  | Stroop | 0.45 | 0.69 | -0.08 | 0.06 |

**Supplementary material D: Delta functions**

In the main text, we discuss in more detail how the DMC fits underestimated the incongruent RTs in slower individuals/tasks. This resulted in an underestimation of the RT cost, in slow RTs in particular, and a qualitative discrepancy between the delta functions in empirical and simulated data (see Appendix C).

We believe that this occurs due to an interaction between boundary separation and the time-to-peak parameter. A short time-to-peak is necessary to produce both fast errors and negative going delta functions (see Figure 1 in main text). The model can produce fast errors and *positive* going delta functions when the decision time is short (i.e. boundary separation is low), as the decision process terminates before the reduction in automatic activation becomes apparent. When the decision time is longer (i.e. boundary separation is high), it is difficult to accommodate the combination of fast errors and positive going delta functions that are observed in Datasets 5 to 7.


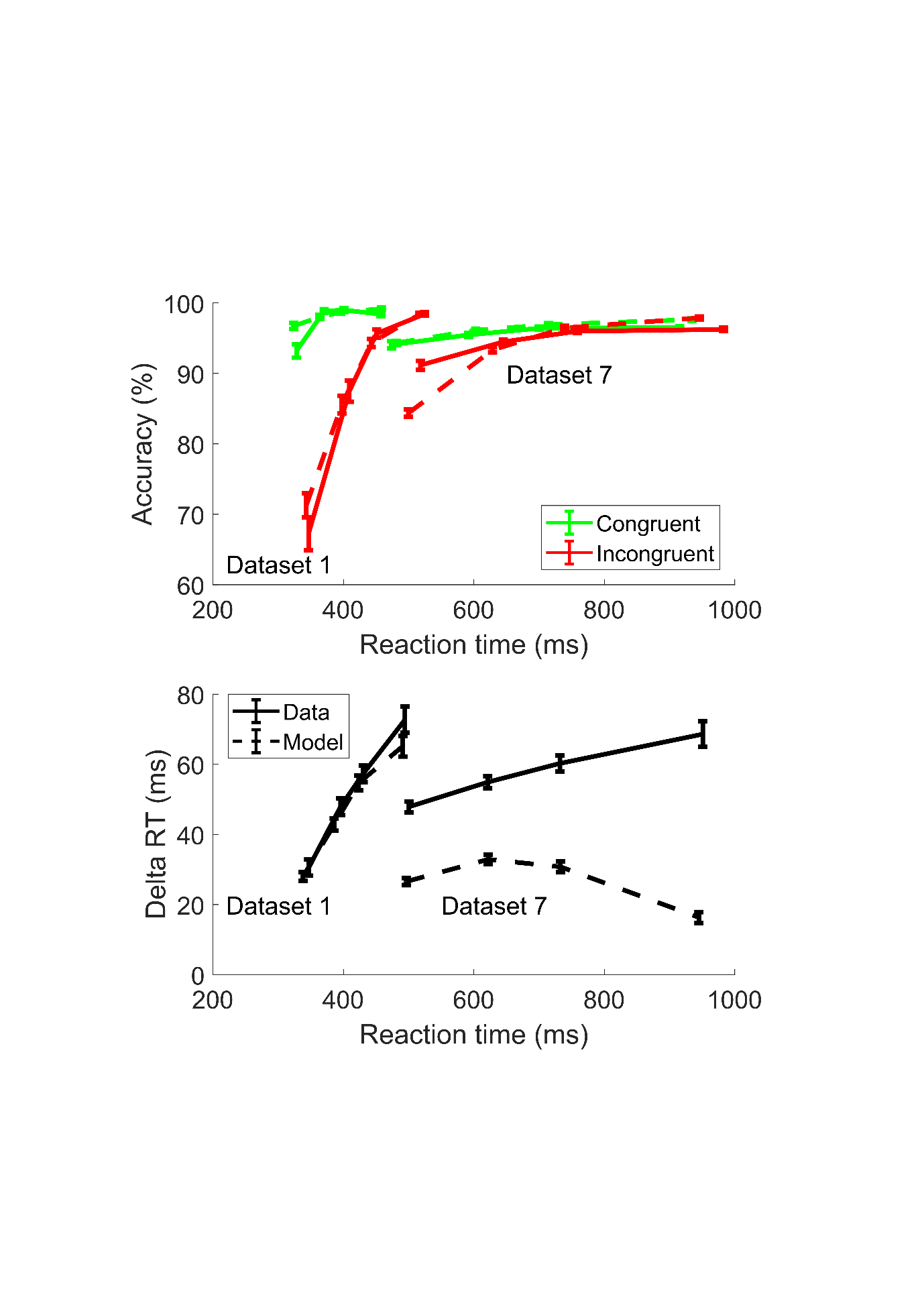


*Figure D1. Conditional accuracy functions (upper panel) and delta functions (lower panel) from the flanker task in Dataset 1 (two-choice, left) and 7 (four-choice, right). These data were chosen for illustration as the Dataset 7 task shows one of the largest discrepancies between the empirical and simulated data. Solid lines represent the empirical data, dashed lines represent data simulated from the best fitting parameters of the diffusion model for conflict tasks. Error bars represent ± 1 standard error. In the upper panel, it can be seen that both tasks produce relatively fast errors in incongruent trials (solid red lines), despite slower RTs overall in Dataset 7. The model (dashed red line) reproduces this pattern, albeit slight overestimating the errors in the fastest quantile in Dataset 7. In the lower panel, the model captures the delta function for Dataset 1 well, but underestimates both the slope and overall RT cost in Dataset 7.*

**Supplementary Material E: Additional simulation analyses**

**Correlations between diffusion model for conflict task parameters and simulated behaviour**

Figure E1 shows the correlations between model parameters and simulated RT costs and error cost for each task scenario (Simon, flanker and Stroop). Within each task scenario we pooled the data points across the two simulated tasks, scenarios (which parameters were correlated), and effect sizes (.3, .5 .7). This resulted in a 40,000 data points per correlation.

If RT costs or error costs were specifically sensitive to conflict processes, they should show strong positive correlations (red) to the conflict parameters in the top two rows, while showing weak or no correlation with the other parameters. Neither RT costs nor error costs correlated exclusively with a single parameter. Both measures correlated with either or both the parameters that constitute the conflict effect - the amplitude and time-to-peak. But each also correlated at least moderately with boundary or drift rate (or both).

Note that the correlation between the RT cost and boundary separation is dependent on the task scenario. There is no correlation in the simulated Simon task, whereas the correlations are positive for the flanker and Stroop. The difference between these task scenarios is that the Simon includes a lower range of the time to peak parameter, which is how the DMC accounts for negative going delta functions (Ulrich et al., 2015). A negative going delta corresponds to an RT cost that decreases in slower RTs. Increasing decision time (boundary separation) under these circumstances allows the RT cost to reduce further, resulting in a negative correlation between boundary separation and RT costs when the time to peak is short.

**
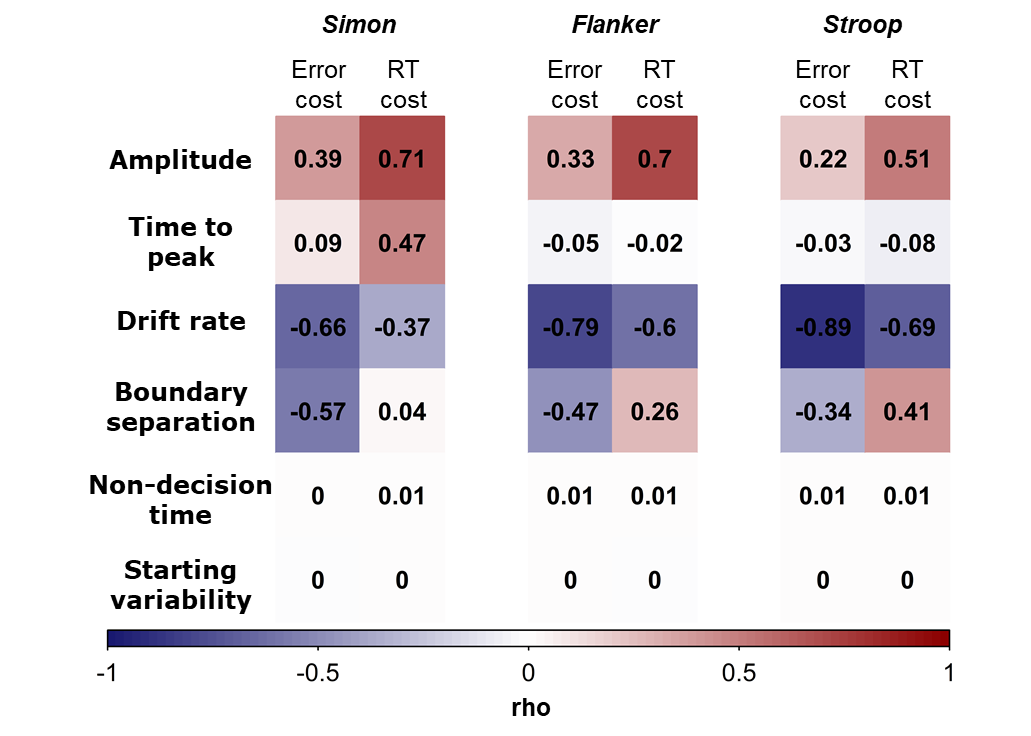
**

*Figure E1.* *Spearman’s rho correlations between parameters of the diffusion model for conflict tasks (rows) and behavioural measures derived from simulated data. Positive correlations are shown in red, negative correlations are shown in blue. Note that both RT costs and error costs show positive correlation (red) with either the amplitude and/or time-to-peak parameters, which correspond to conflict processing. However, they also all show correlation with either boundary separation or drift rate. The correlation between boundary separation and RT costs differs between the simulated tasks because it is dependent on the range of the time to peak parameter.*

**Correlations in different simulated tasks.**

The correlations in Figure 5 in the main text are between two variants of the same task (i.e. flanker with flanker, Stroop with Stroop). The reason for this choice is that we would expect correlations between tasks to be highest when the underlying mechanisms manifest in a similar way. For example, the Simon task typically shows fast errors on incongruent trials and negative going delta functions, whereas the Stroop shows neither. We consider these simulations to represent upper limits for what could be expected from correlations between different tasks.

Here we perform the same analysis looking across our different simulated tasks (Figure E2). As a reminder, we implemented different tasks by changing the average value of the time-to-peak parameter to match those observed in our empirical fits. We used the same means and standard deviations for the other parameters in each task. As we anticipated, these correlations did not exceed those we report in Figure 5. However, we see the same pattern of attenuated correlations in scenarios 1 to 3, and large correlations in the absence of correlated conflict parameters (scenario 4).

*
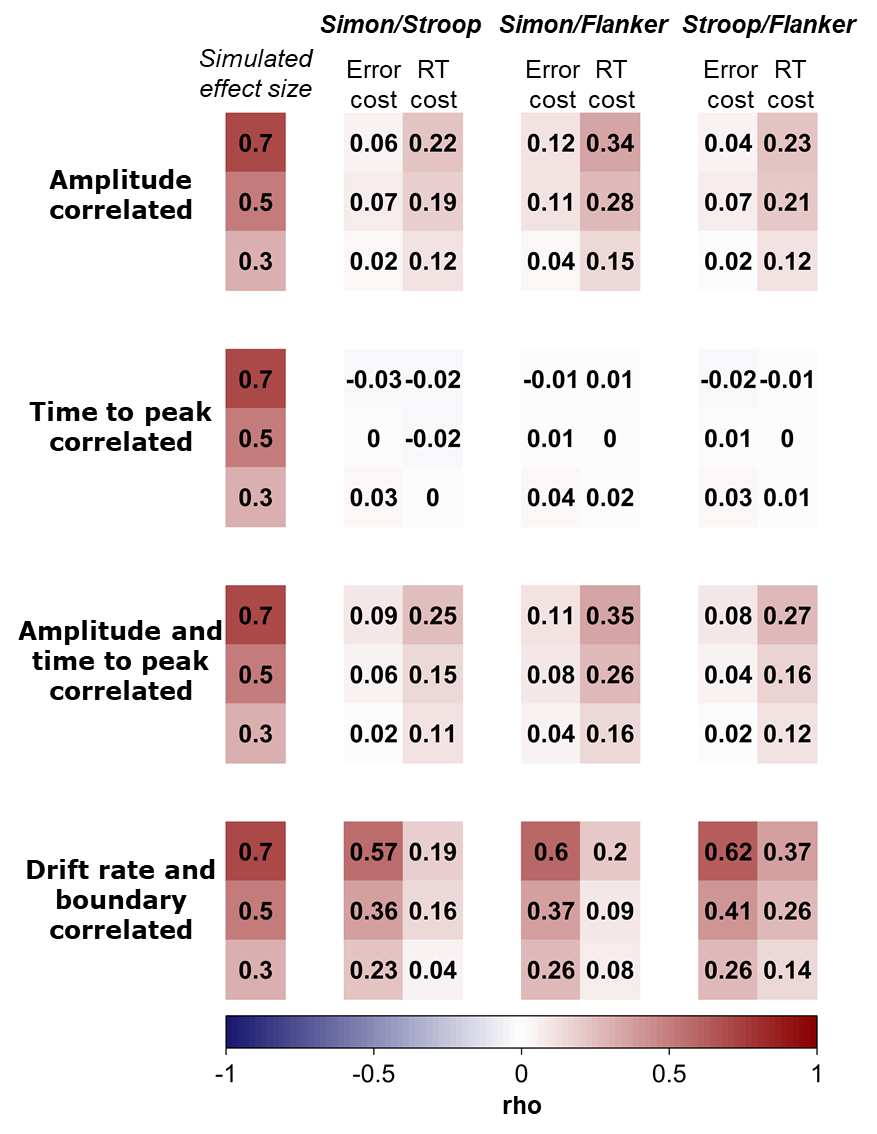
*

*Figure E2.* Spearman’s rho correlations between performance costs calculated from two simulated datasets using the diffusion model for conflict tasks. The strength of the between-task correlation in the conflict parameter(s) is given in the “Simulated effect size” column. The columns to the right of this show the between-task correlations in the simulated error and RT costs respectively. The correlation between other model parameters (boundary separation, drift rate and non-decision time) was set to 0 in the first three scenarios. In the fourth scenario, the correlation in conflict parameters was set to zero, and the simulated effect sizes were applied to the non-conflict parameter correlations.

**Correlations in simulated data using distributions observed in model fits.**

Figure E3 below complements Figure 5 in the main text. Here, we repeat the simulations assuming the strongest correlation of r=.7 in conflict processing parameters, though now we use the means and standard deviations we observed in our own fits of the flanker and Simon task (see Appendix C) to generate the data. The observed correlations do not exceed those in Figure 5 in the main text (compared to the scenario where a correlation of r=.7 was imposed in both A and tau).


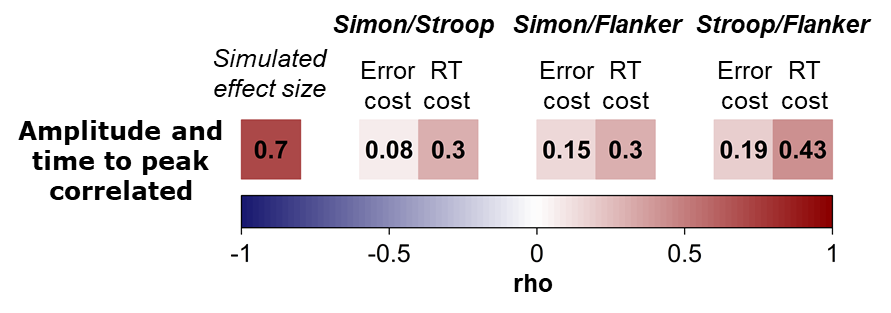


*Figure E3. Spearman’s rho correlations between performance costs calculated from two simulated datasets using the diffusion model for conflict tasks. A correlation of r=.7 was imposed on the generation of response conflict parameters (A and tau). The correlation between other model parameters (boundary separation, drift rate and non-decision time) was set to r=0*

**Supplementary Material F: Plots of model fits**

The following pages show scatter plots of the observed data against data simulated from the best fitting parameters for each dataset and task. The scatter plots show the data corresponding to the Pearson’s correlations reported in Tables C2 to C4. We plot each individual’s observed and simulated accuracy in each condition, along with the 25^th^, 50^th^ (median) and 75^th^ percentiles of the RT distribution. For RTs, the black circles reflect correct RTs and coloured crosses reflect error RTs.

The model fits capture the rank order of participants in all task/datasets (there is a positive correlation between the observed and simulated data points). The most notable deviations are in Datasets 5 to 7, where the speed of slower RTs is underestimated.

We also plot the conditional accuracy functions (CAFs)and delta functions for each task/dataset. The solid lines reflect the empirical data and the dashed lines reflect the model fits. The CAFs are generally captured well (the solid and dashed lines are closely aligned). The flanker and Simon tasks show the expected pattern of relatively fast errors to incongruent stimuli, whereas the CAFs are relatively flat for the Stroop task.

The DMC predicts different patterns of errors for congruent and neutral trials, whereas performance is similar in the observed data. This is because the DMC assumes that the automatic activation is symmetrical across congruent and incongruent trials. Future applications of the DMC may benefit from including a neutral condition and estimating the amplitude separately in incongruent and congruent trials.

The underestimation of slow reaction times can be clearly seen in the delta functions in datasets 5:7, and the Stroop task in dataset 2. This results of a reduction in the RT cost in slower RTs.


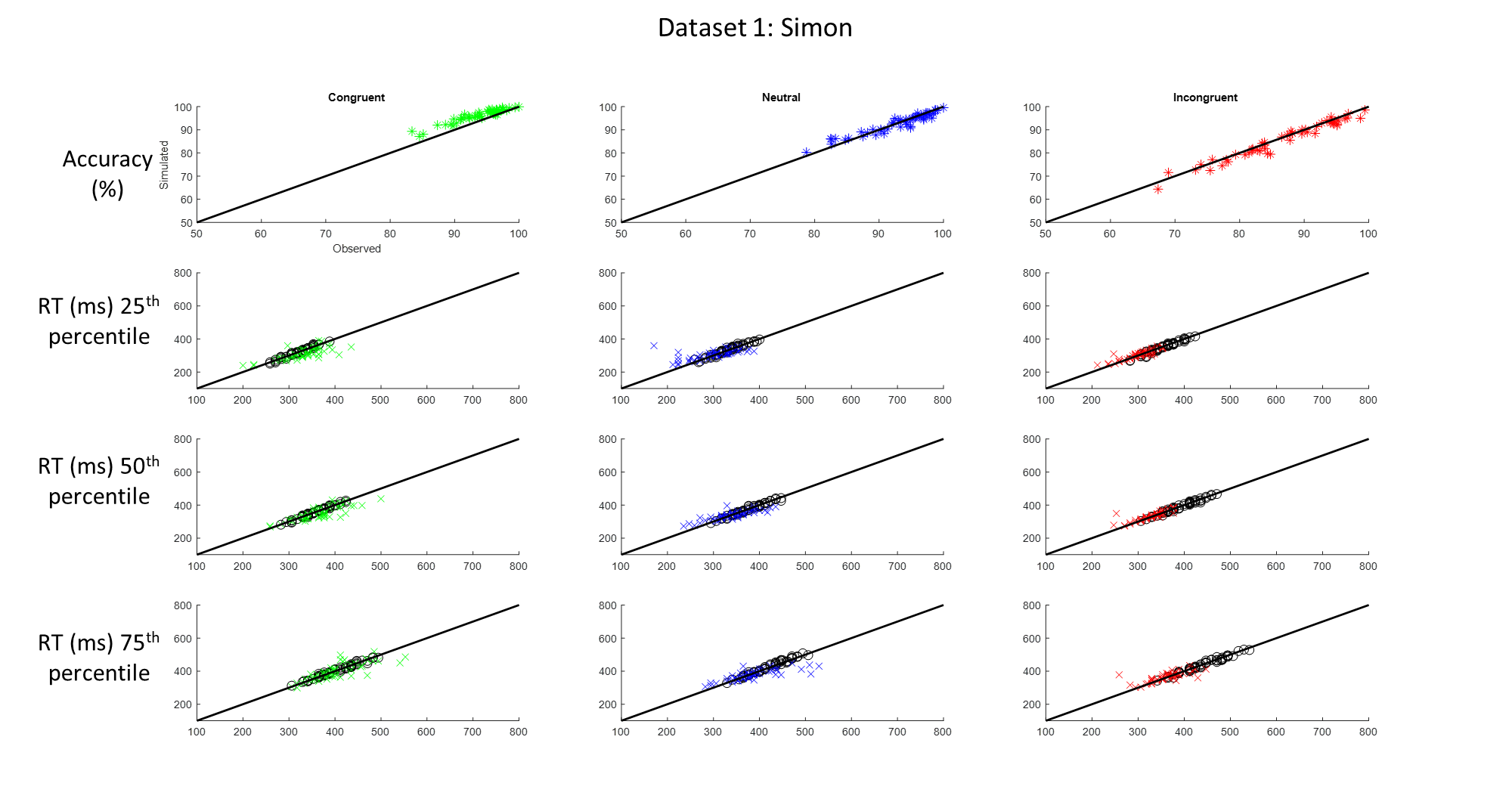


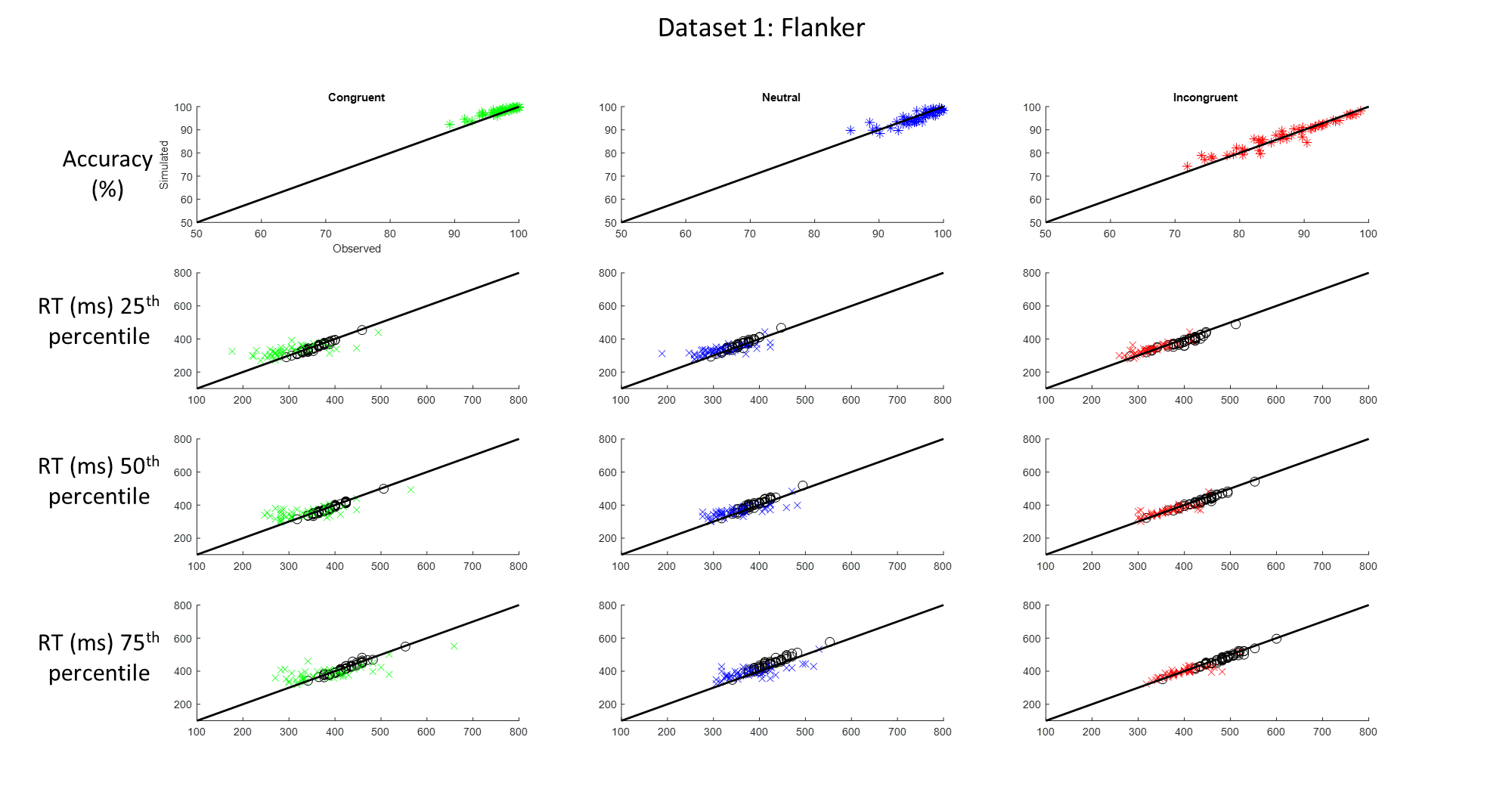


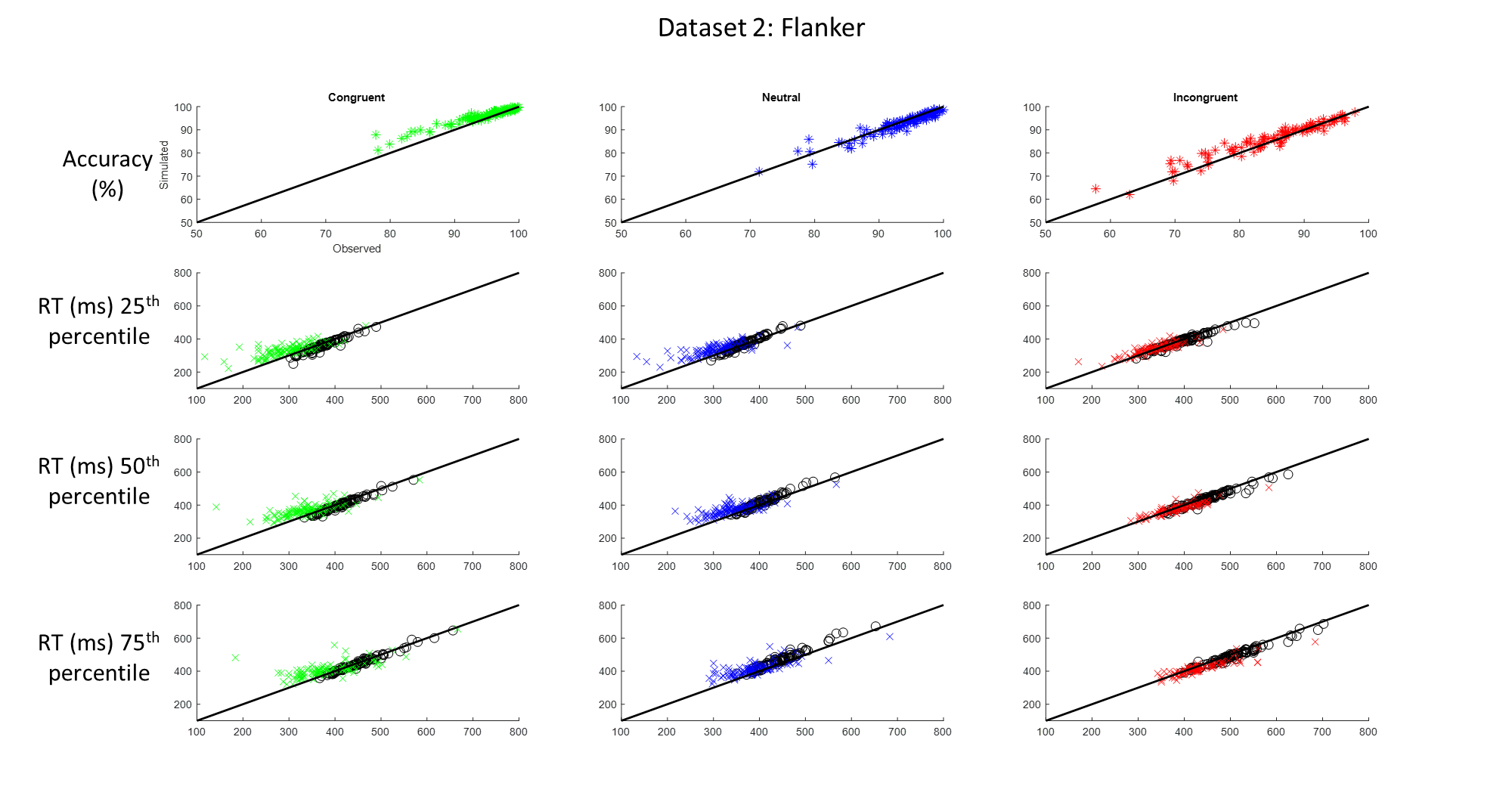


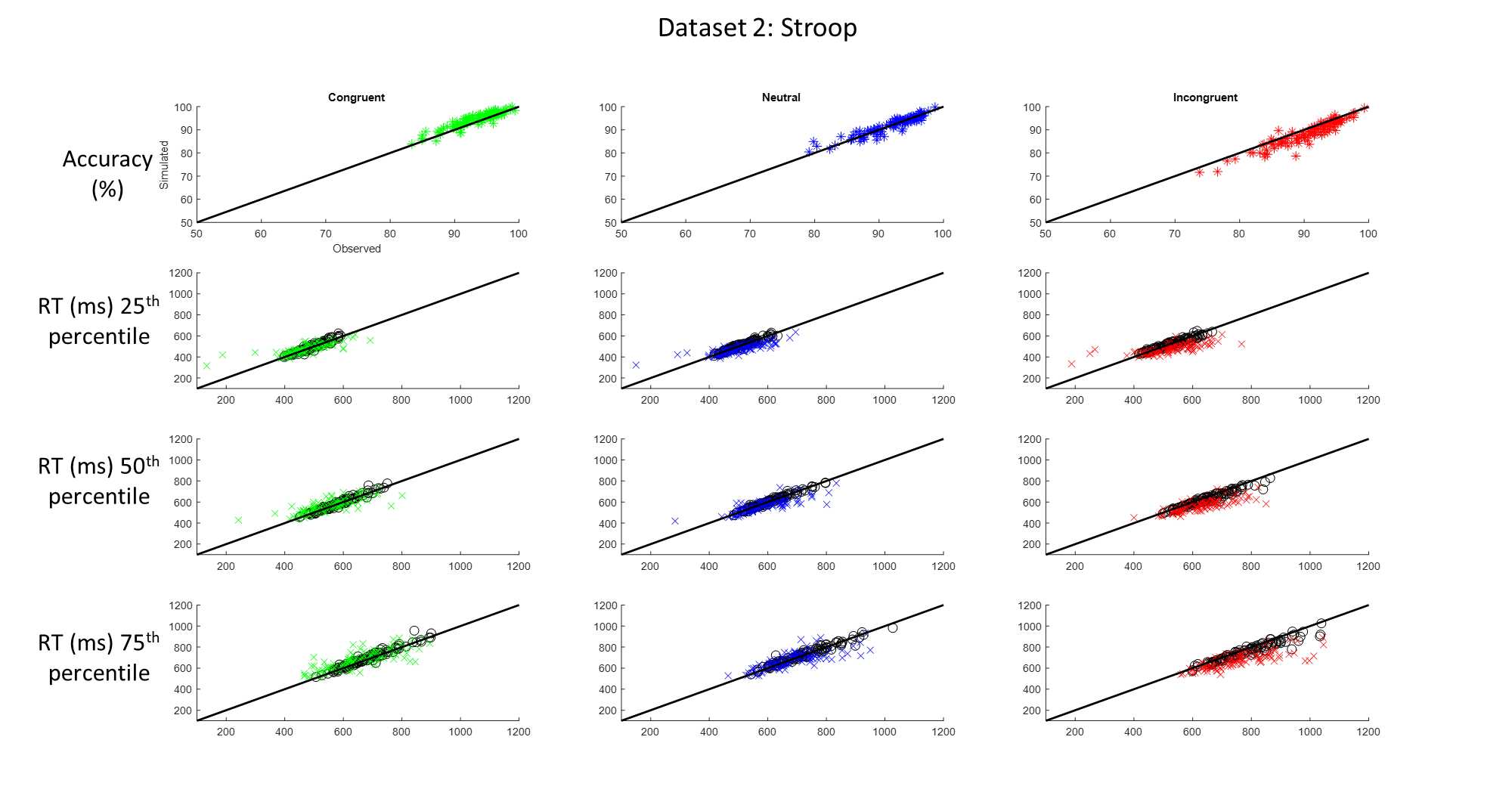


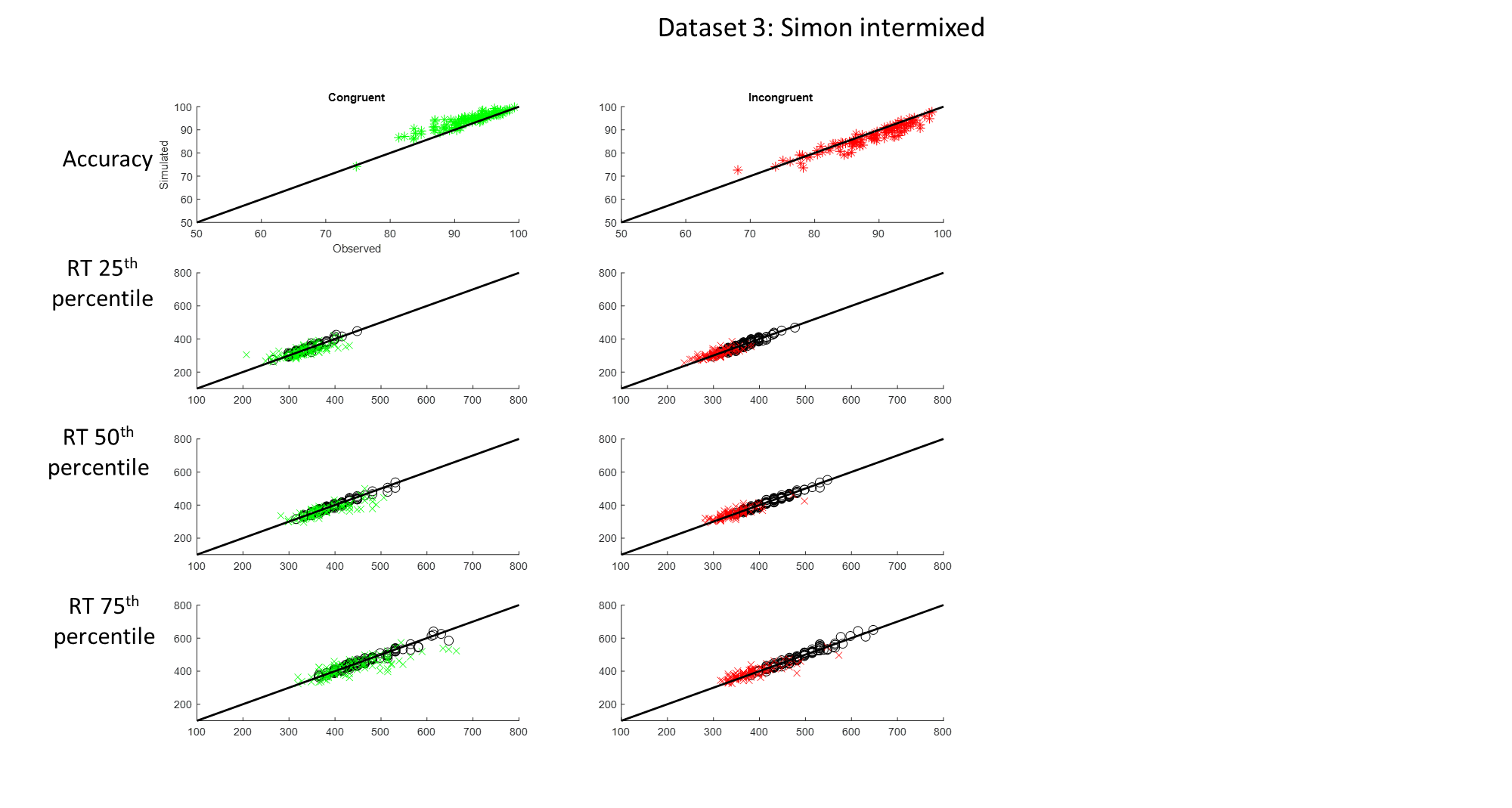


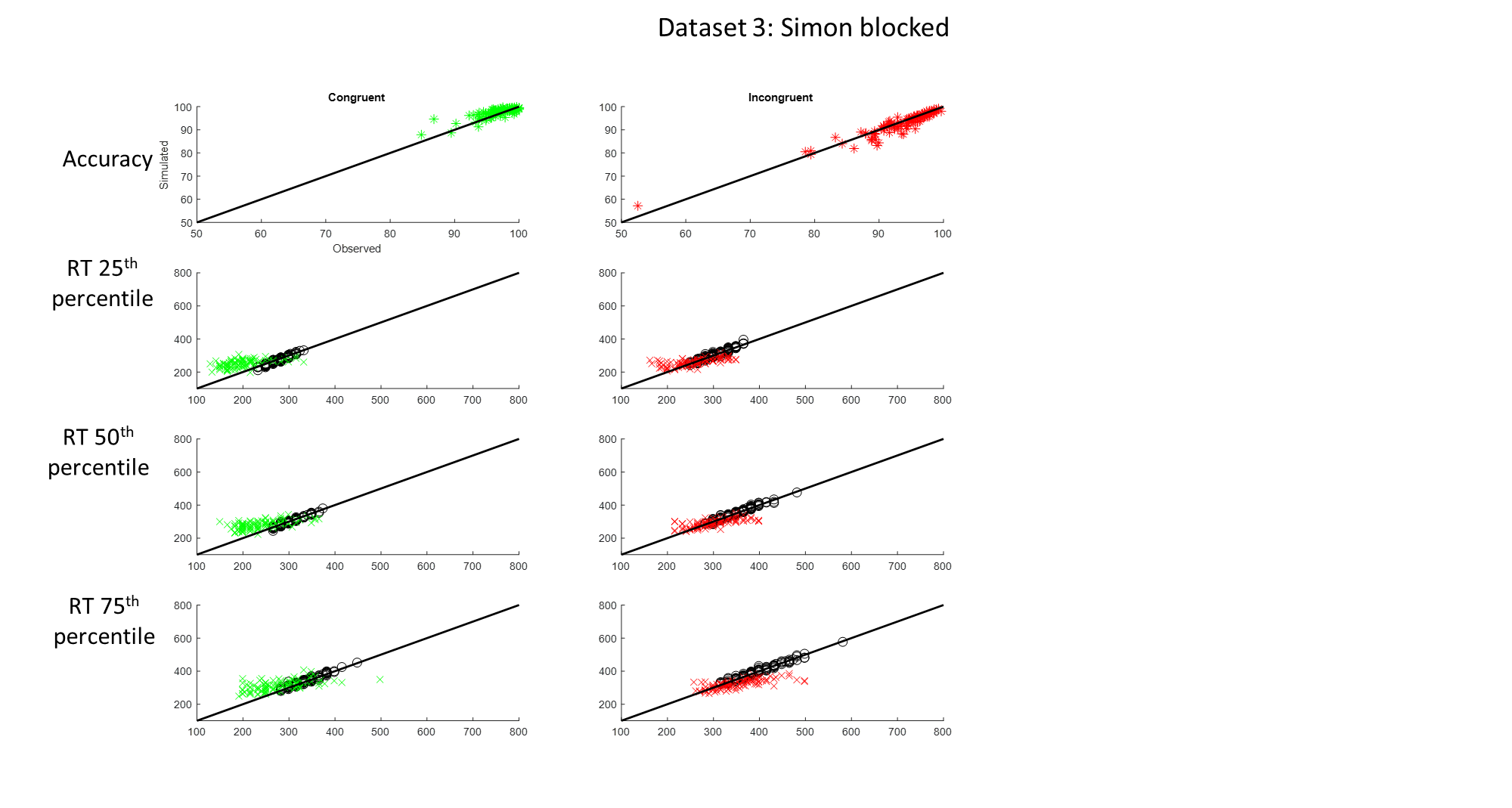


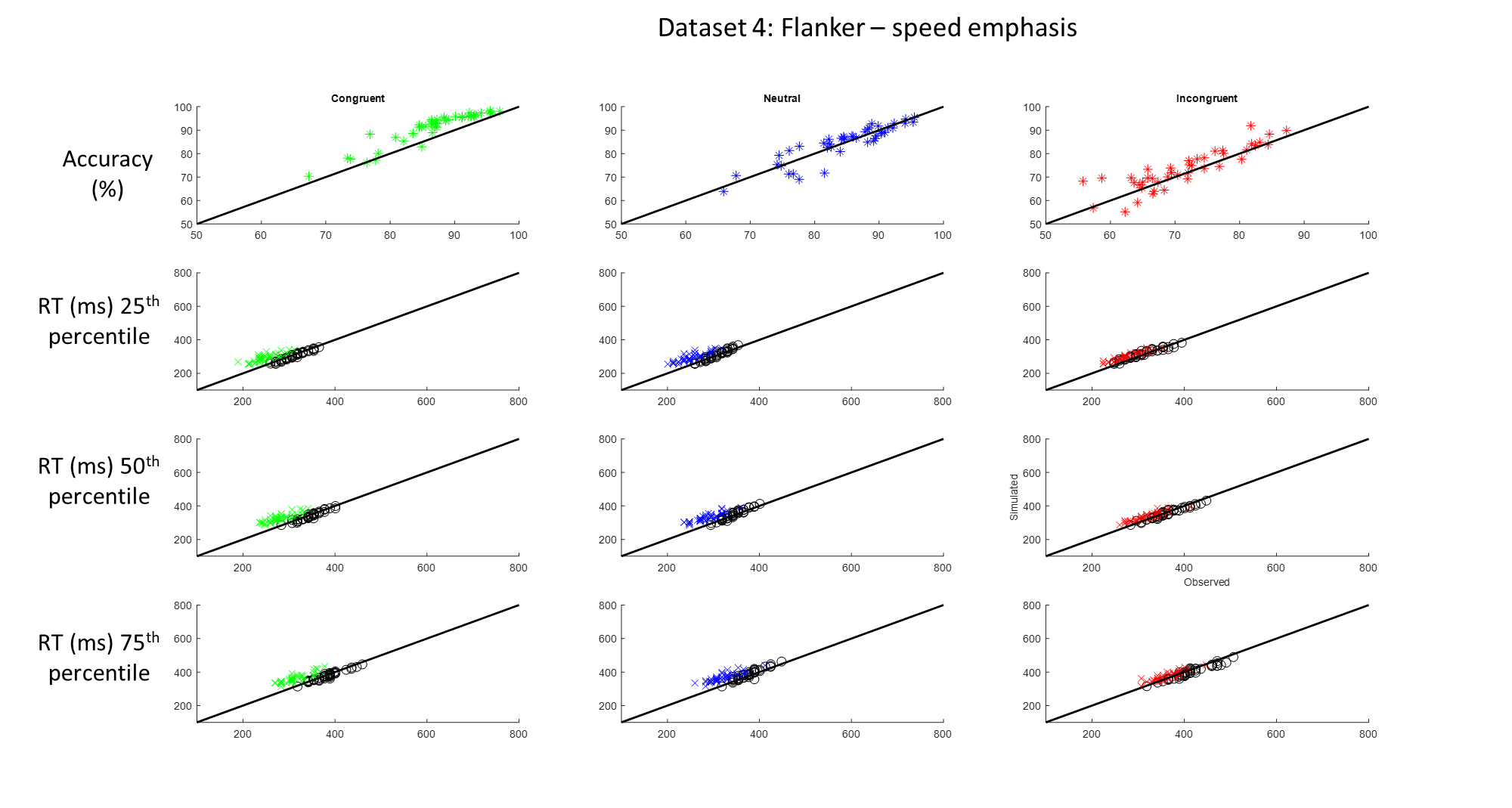


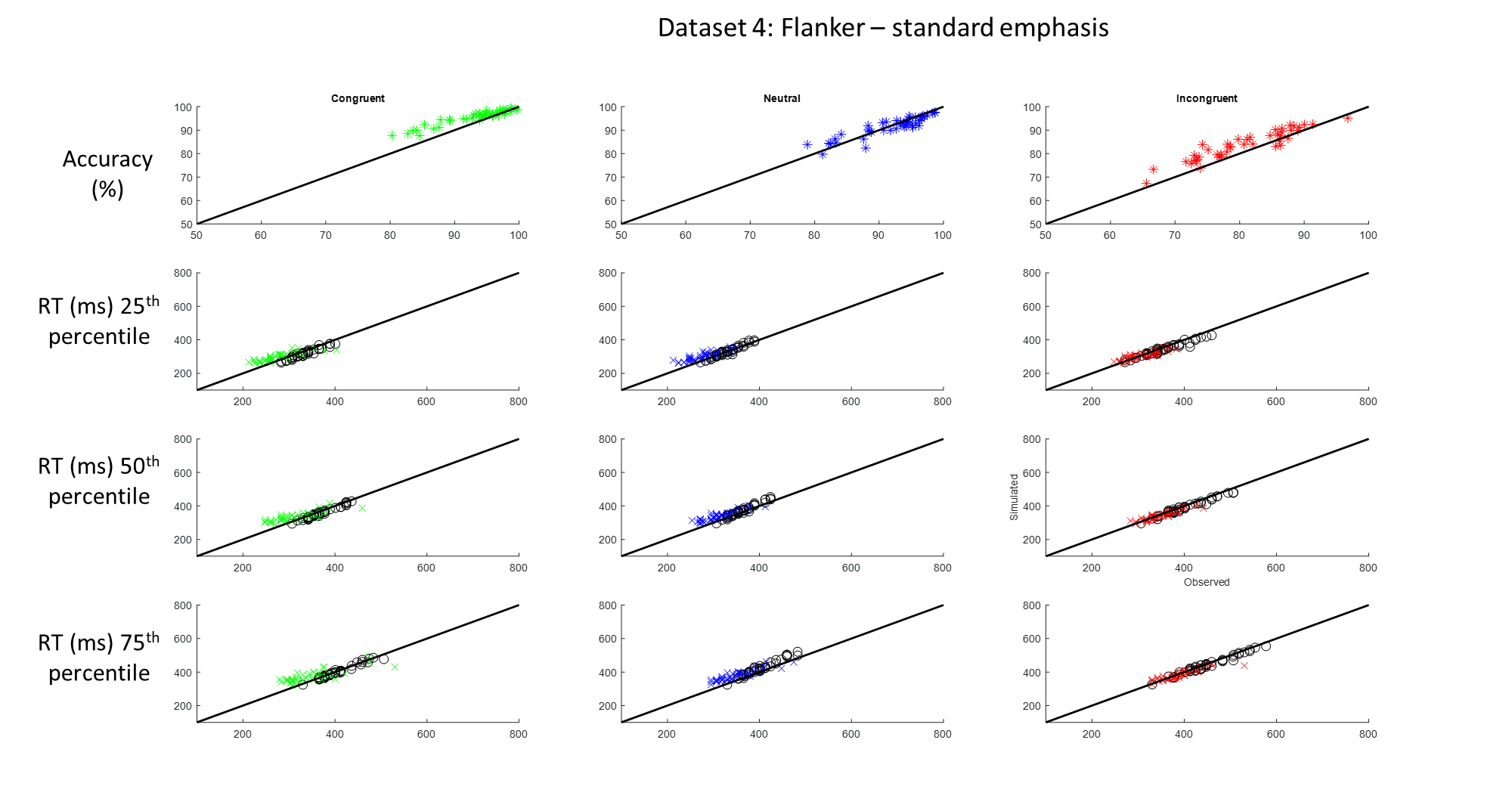


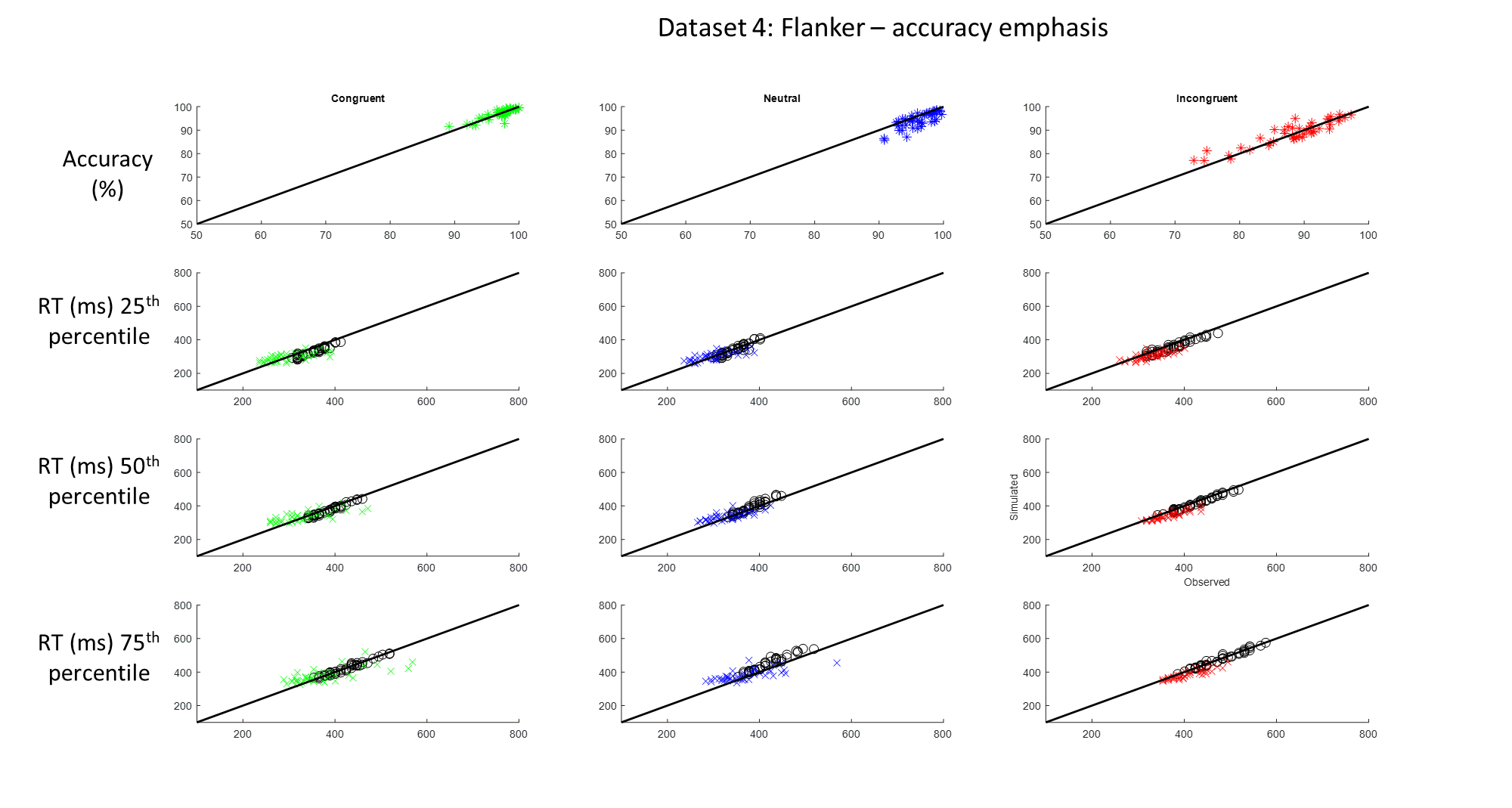


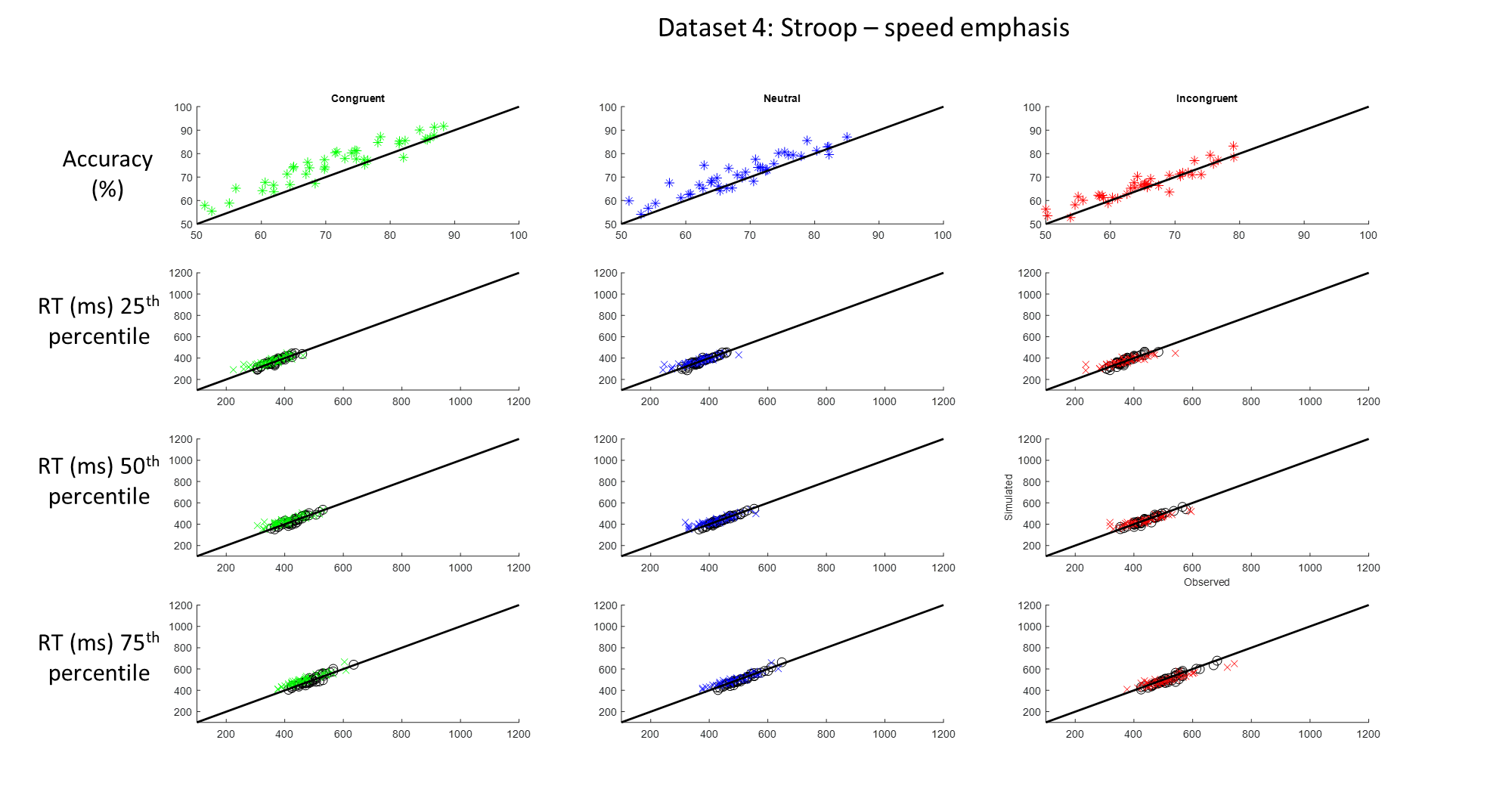


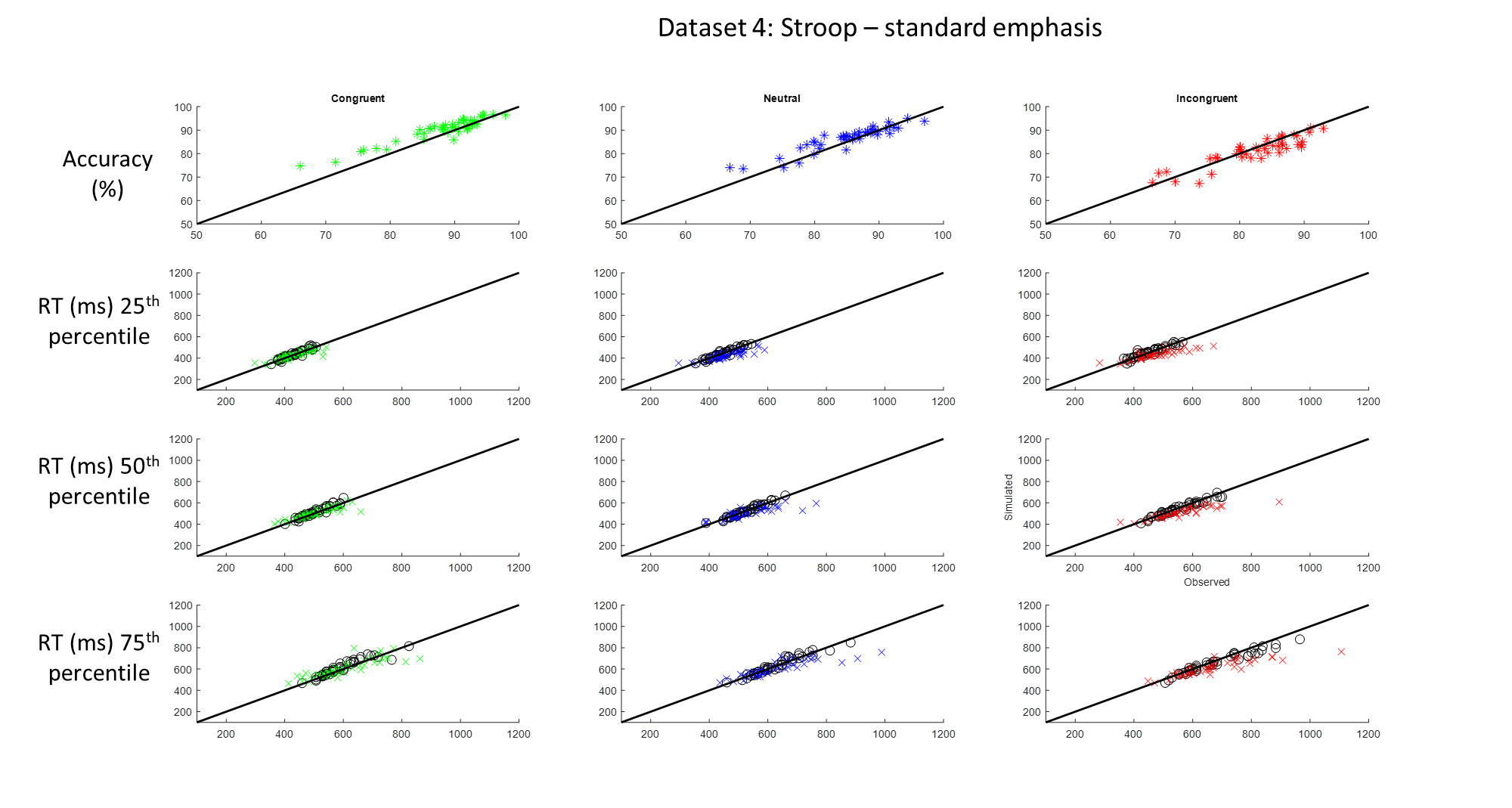


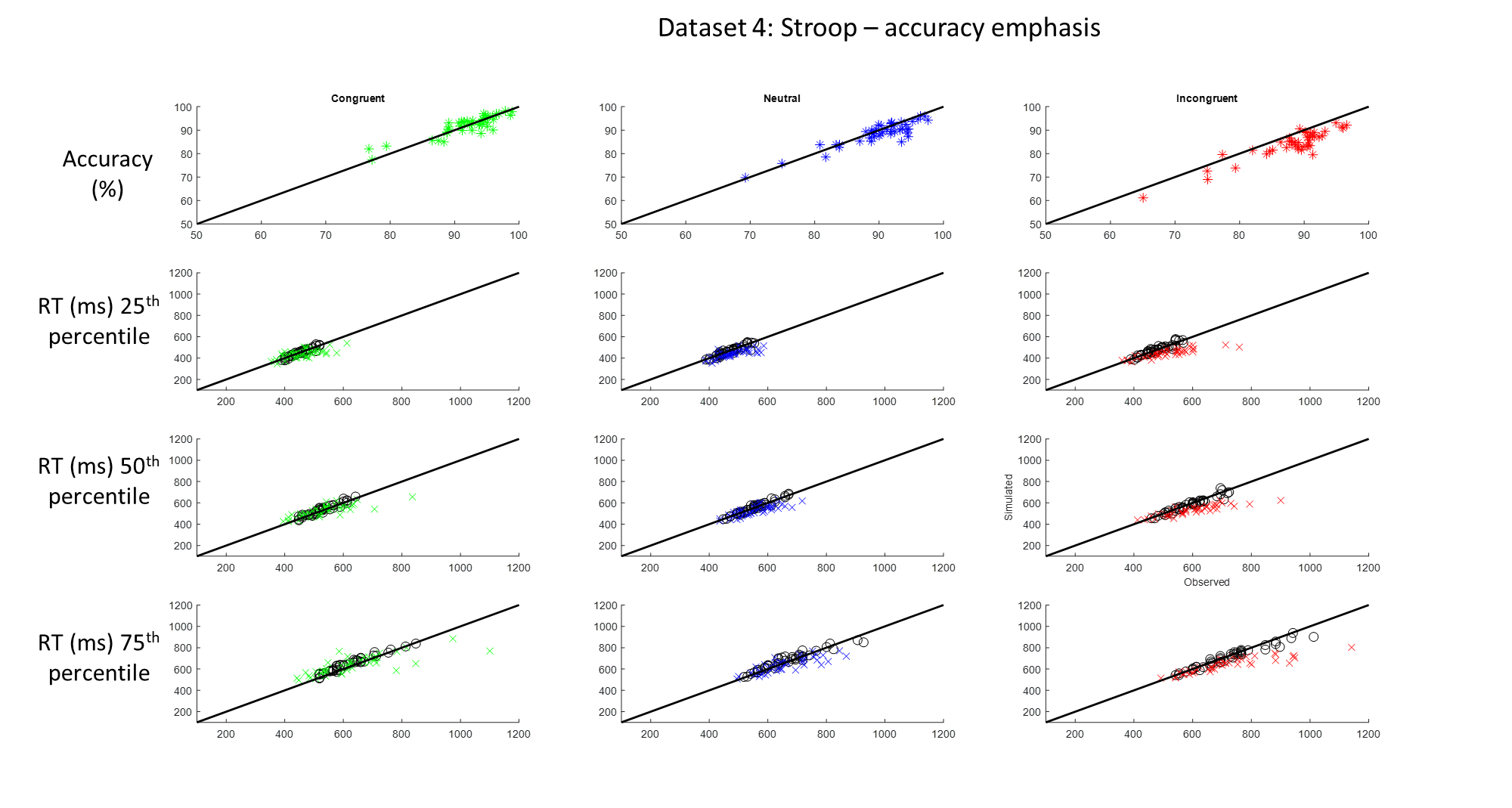


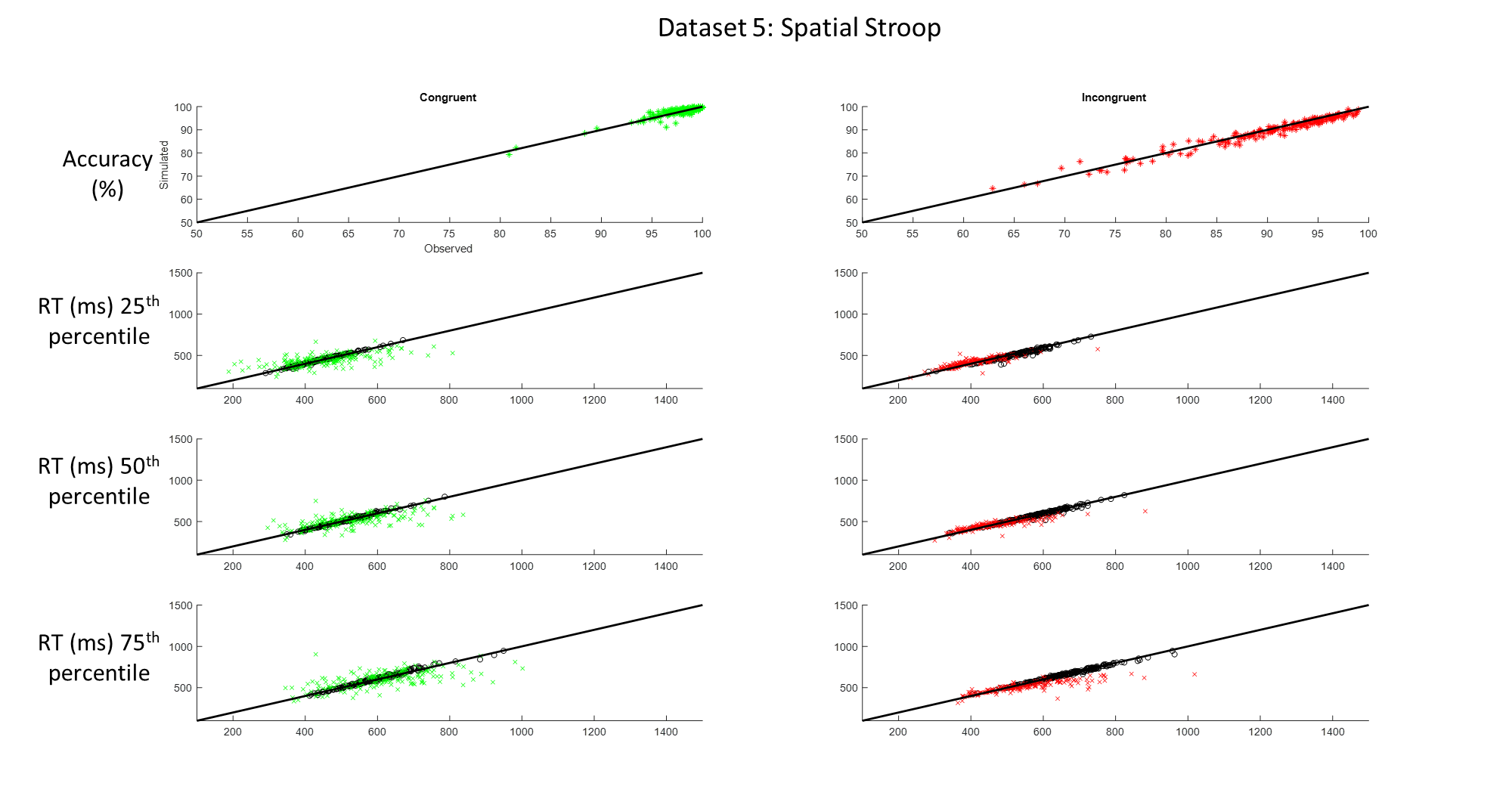


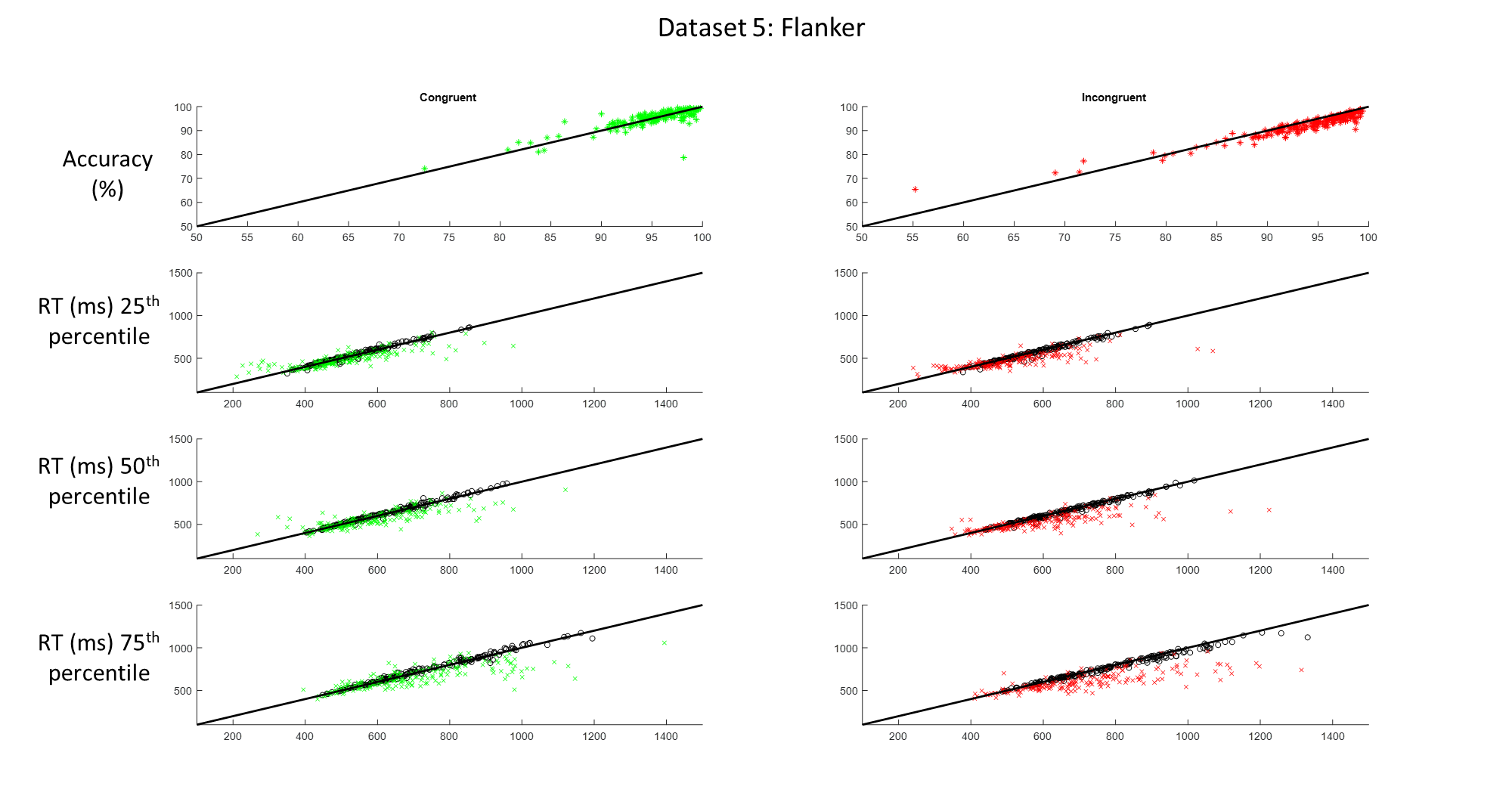


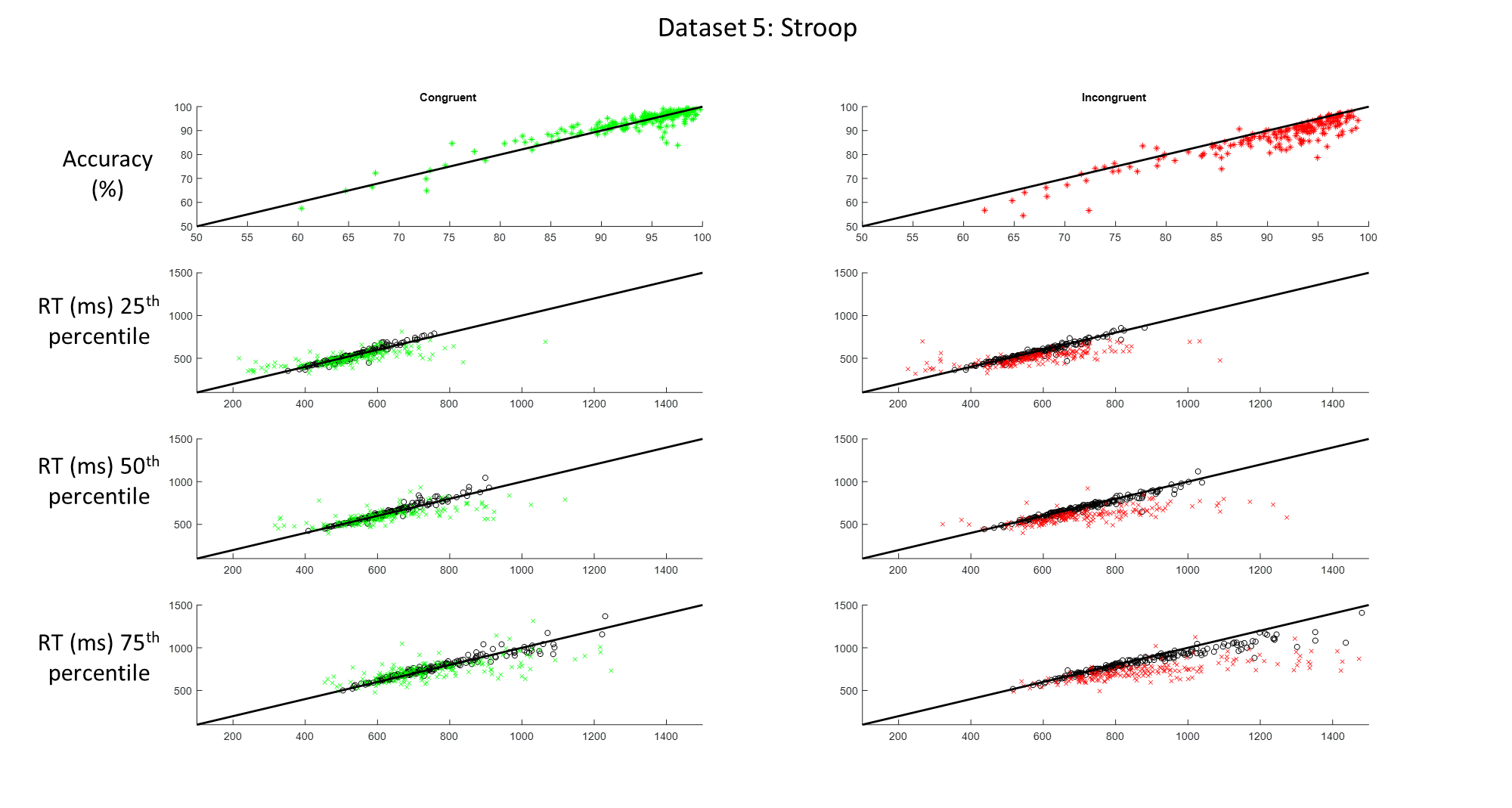


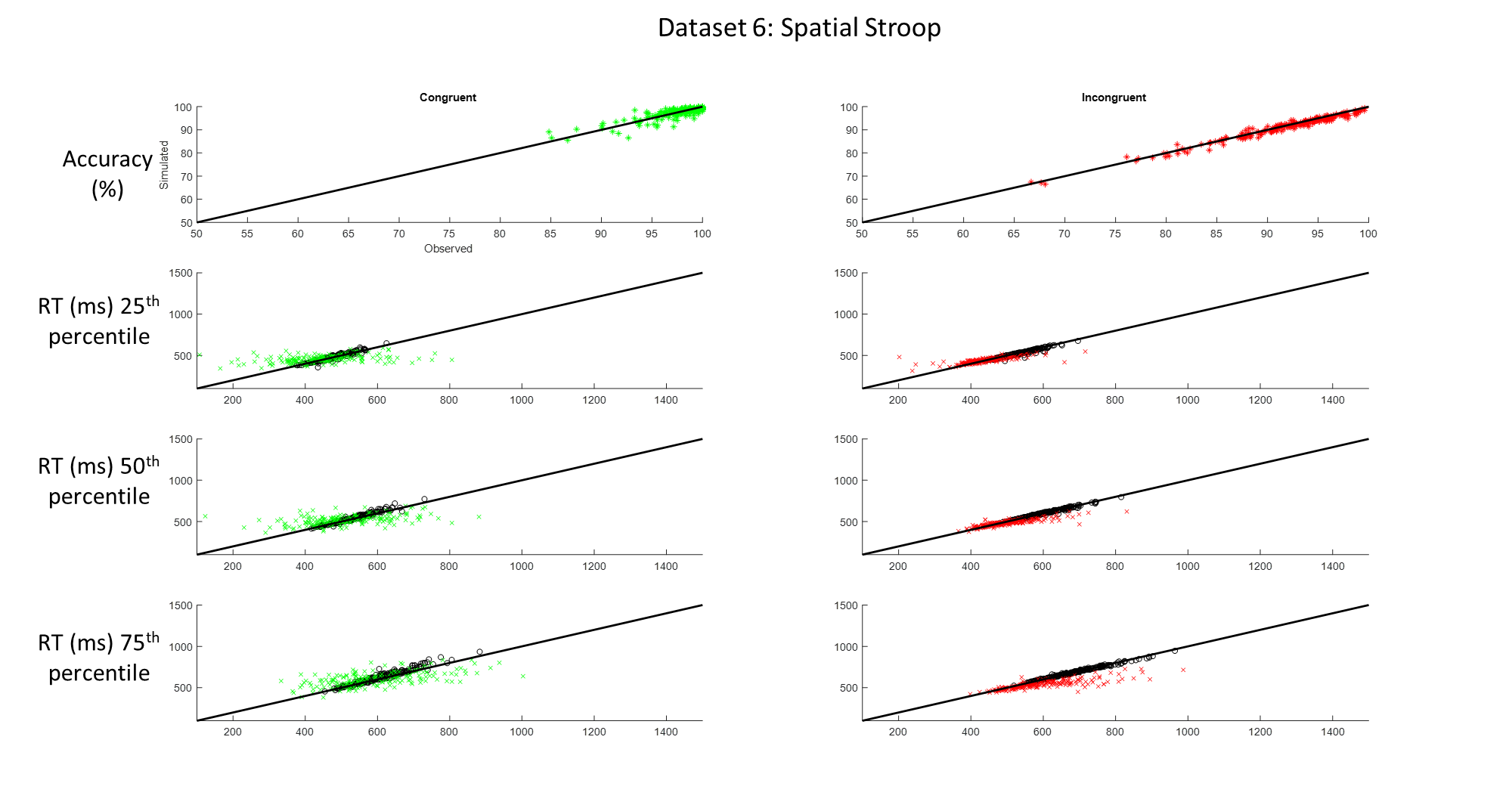


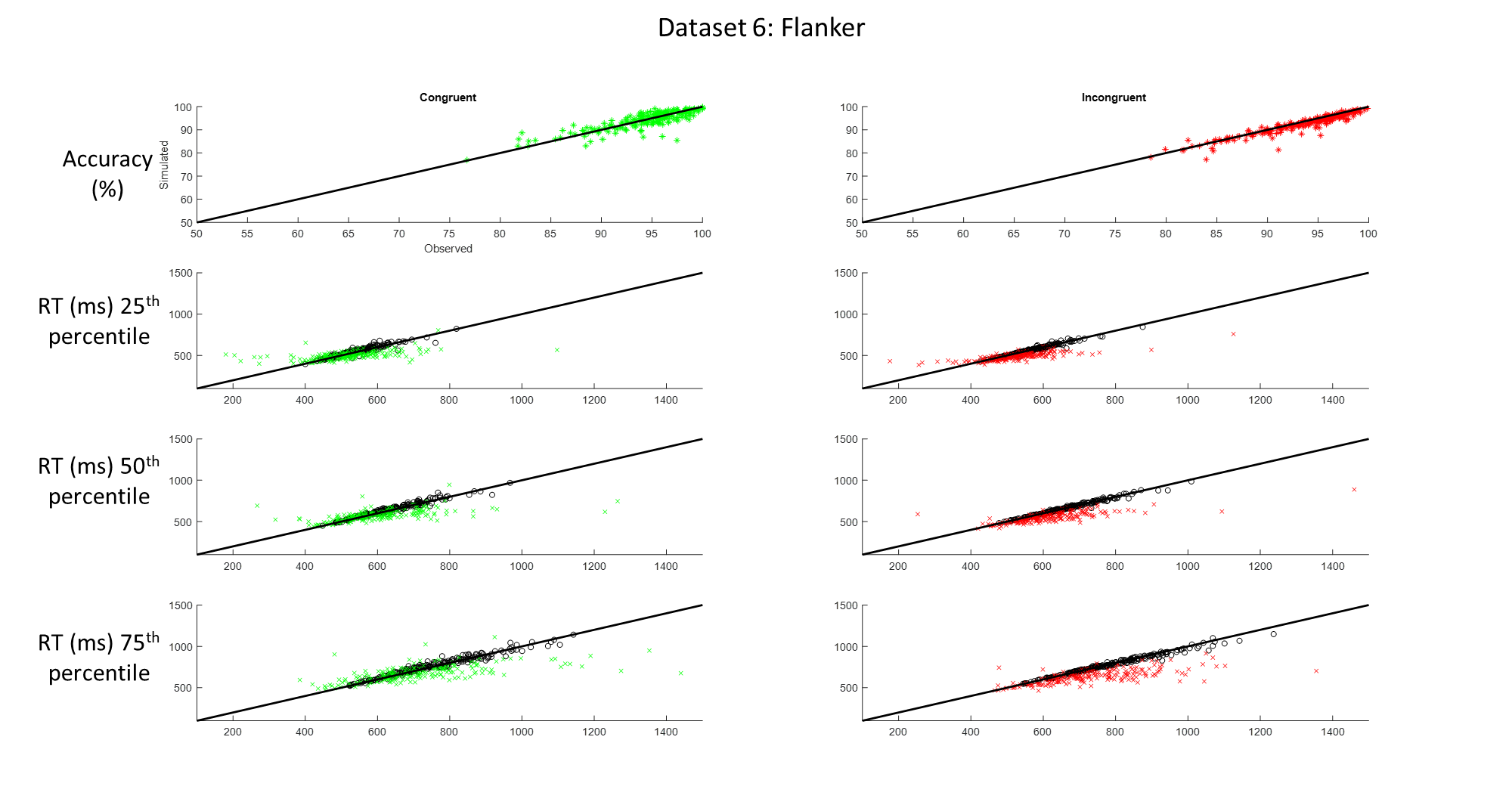


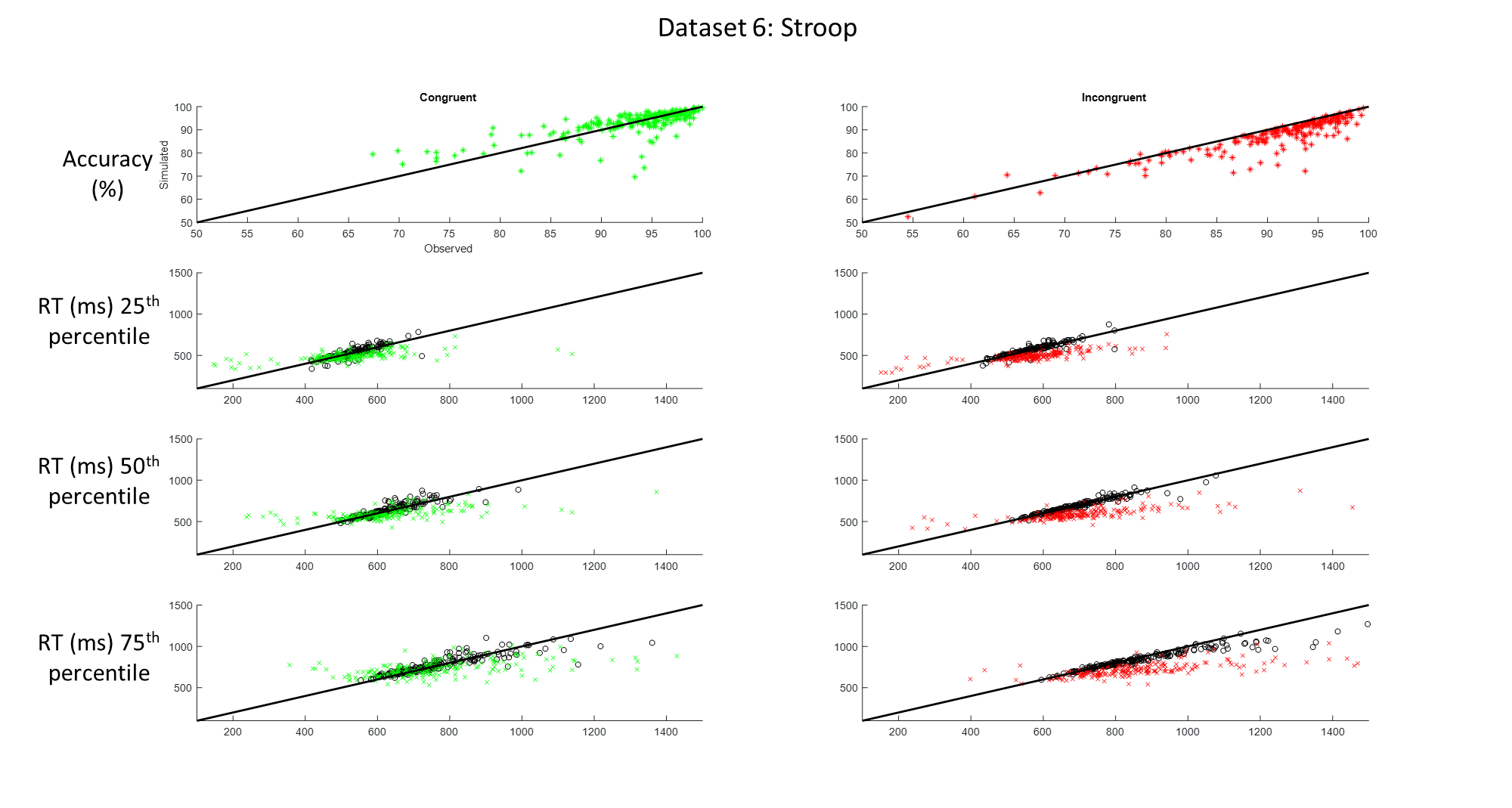


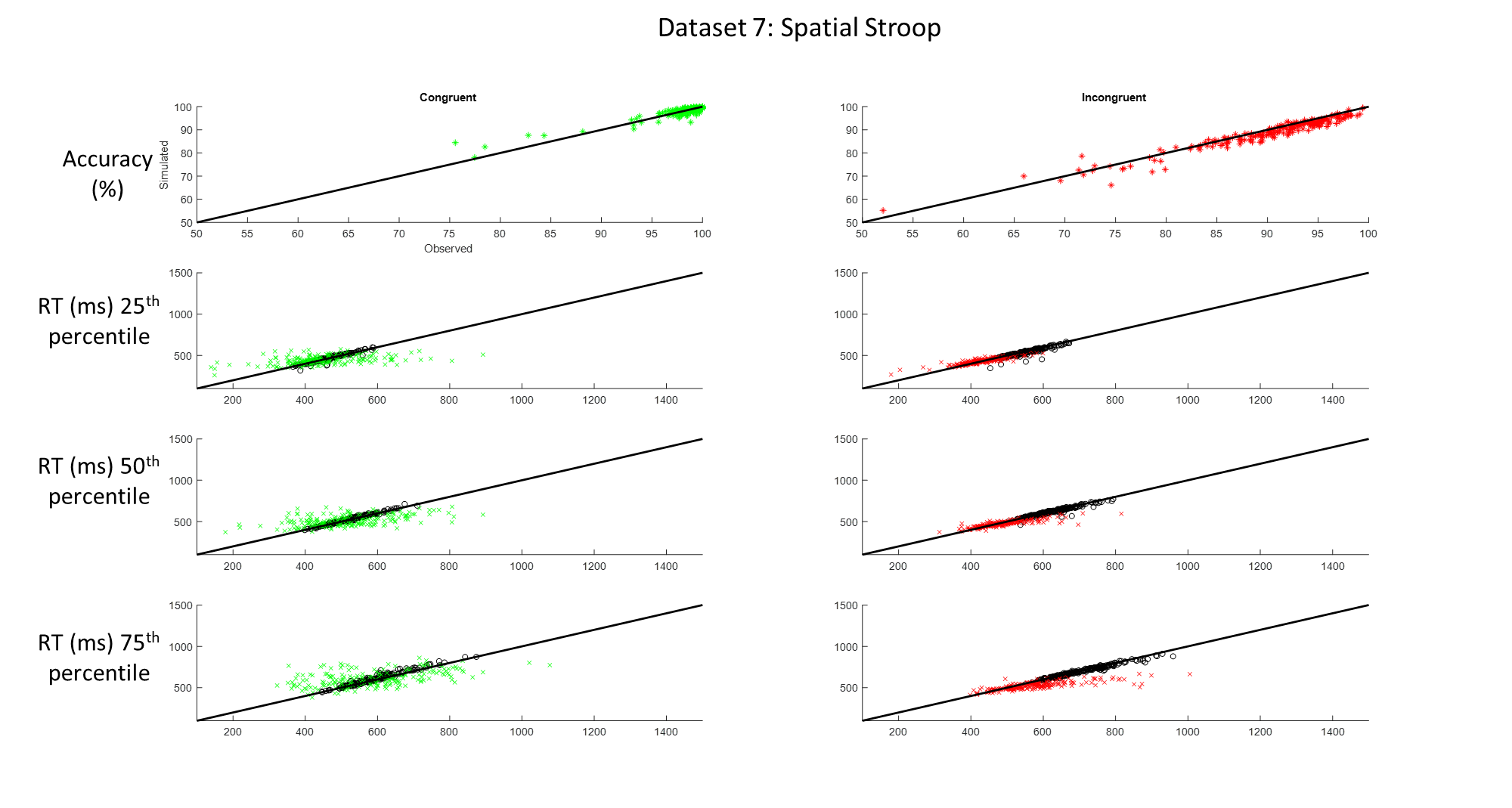


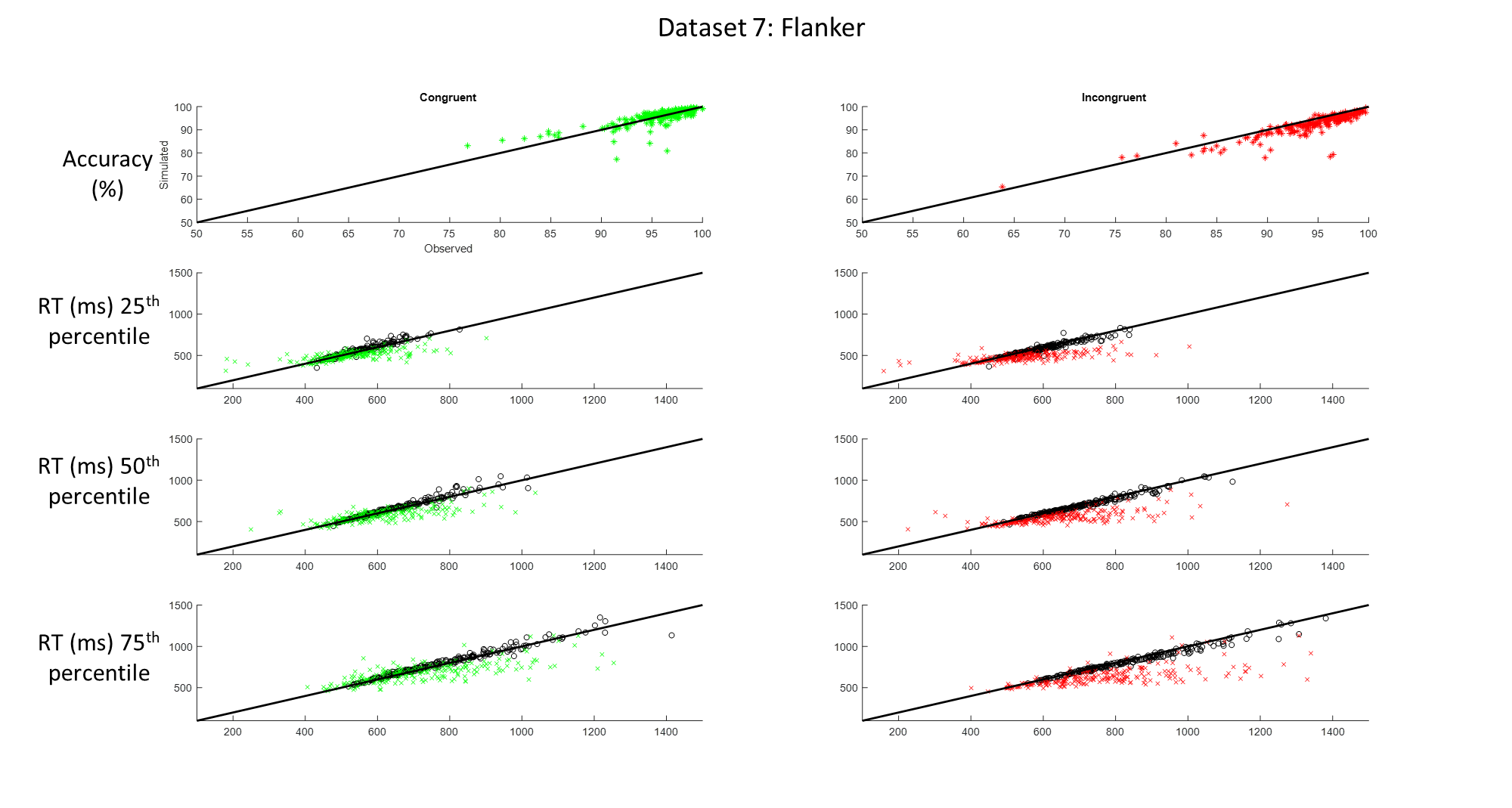


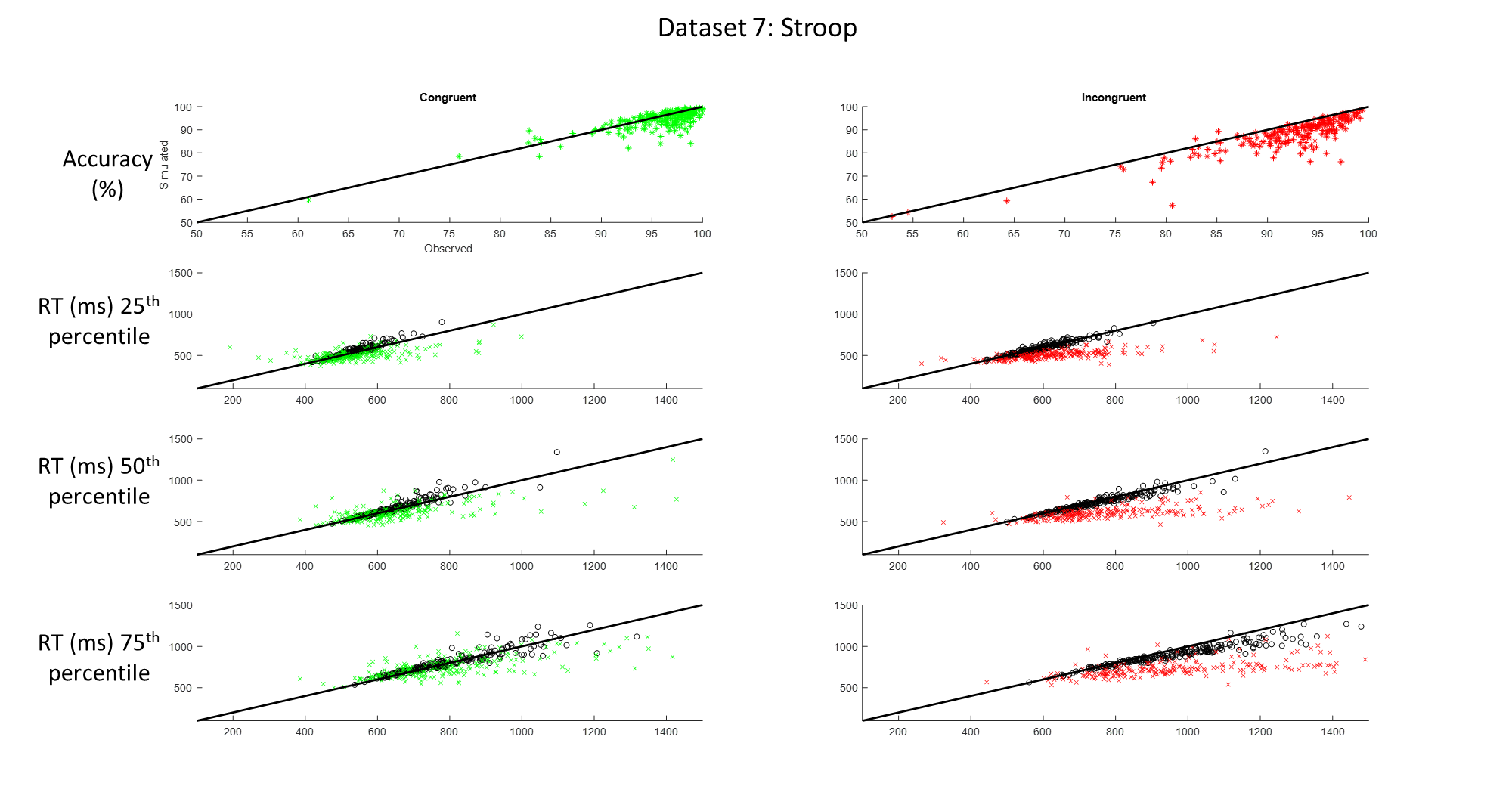


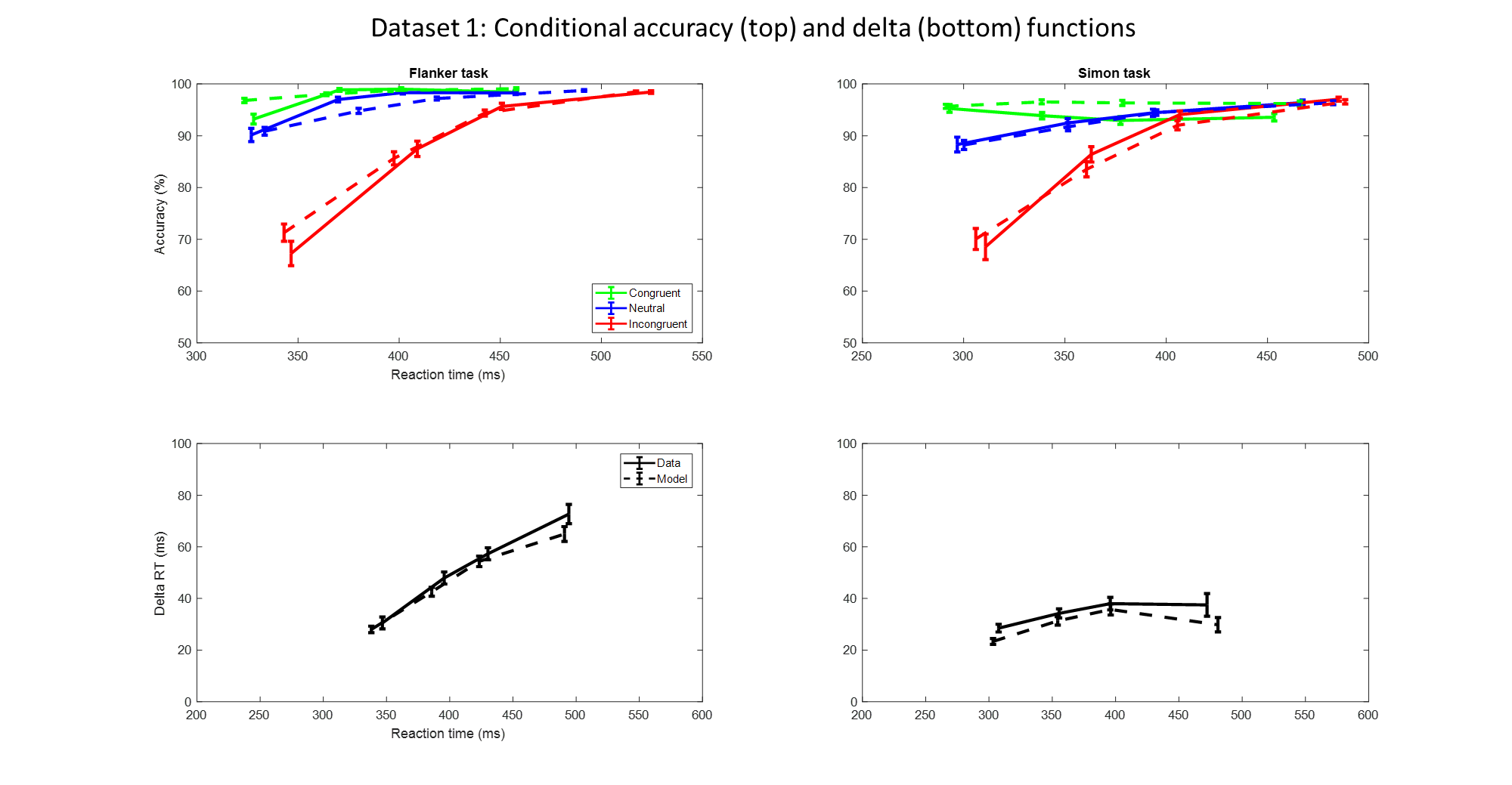


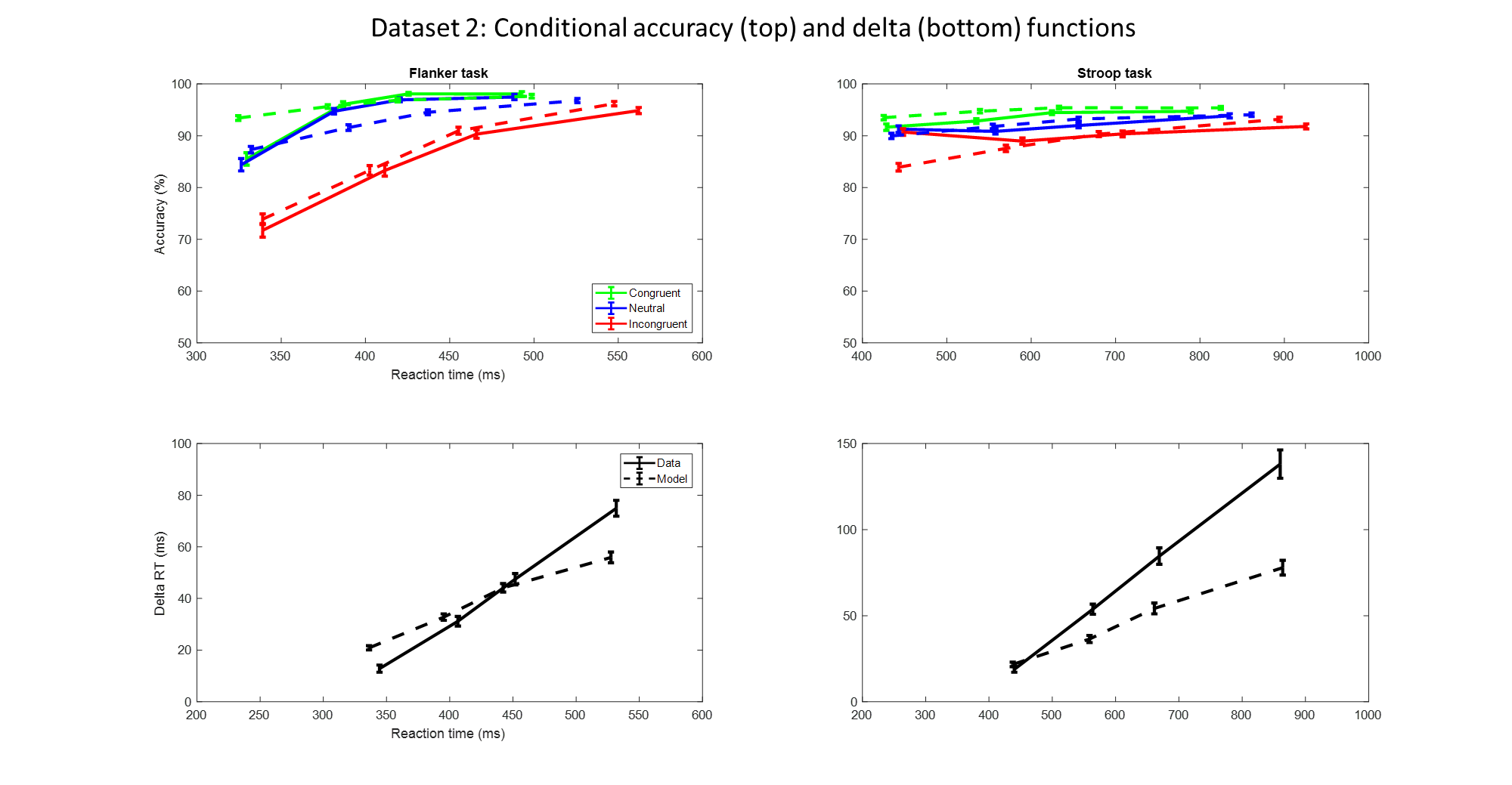


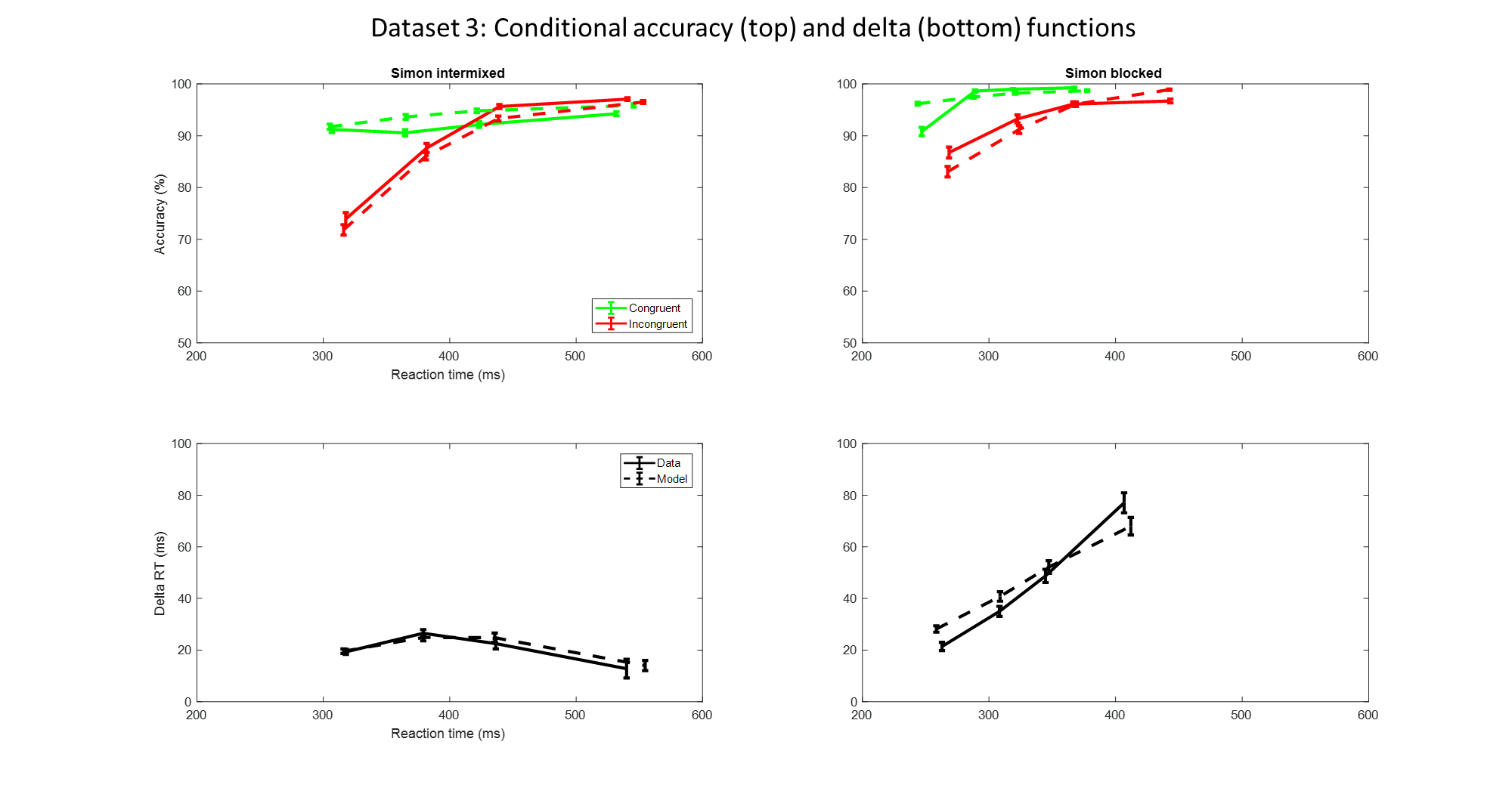


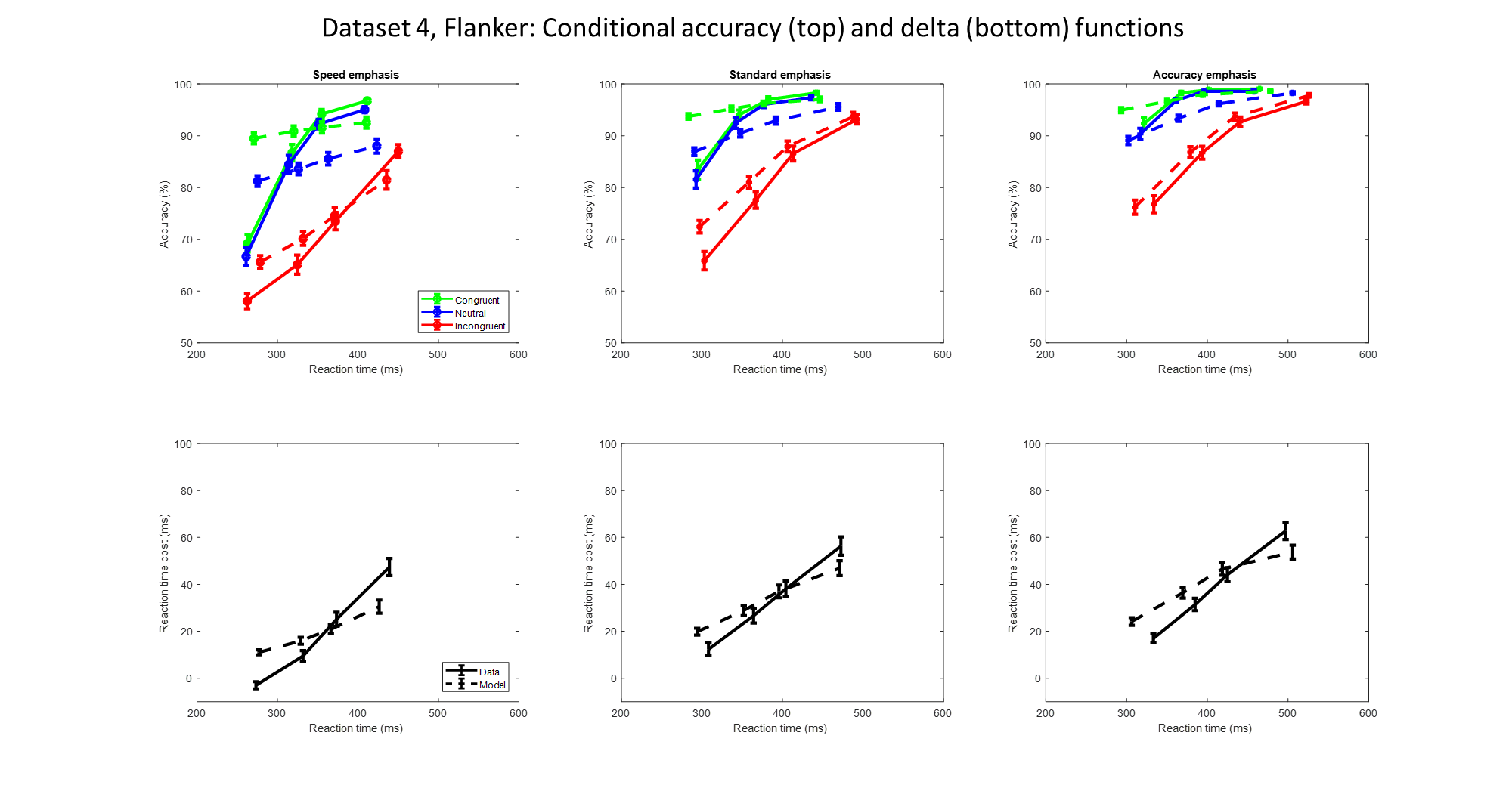


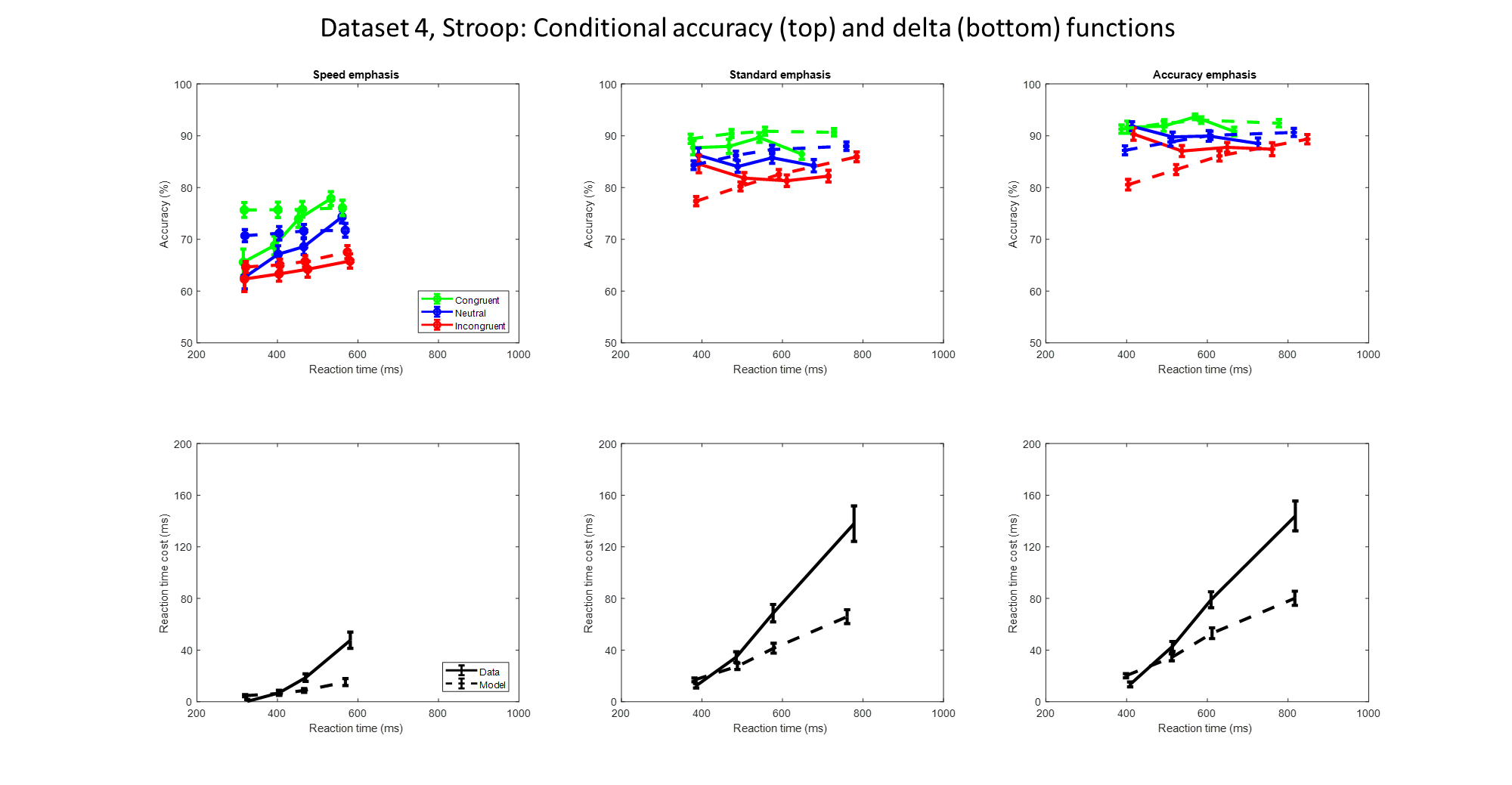


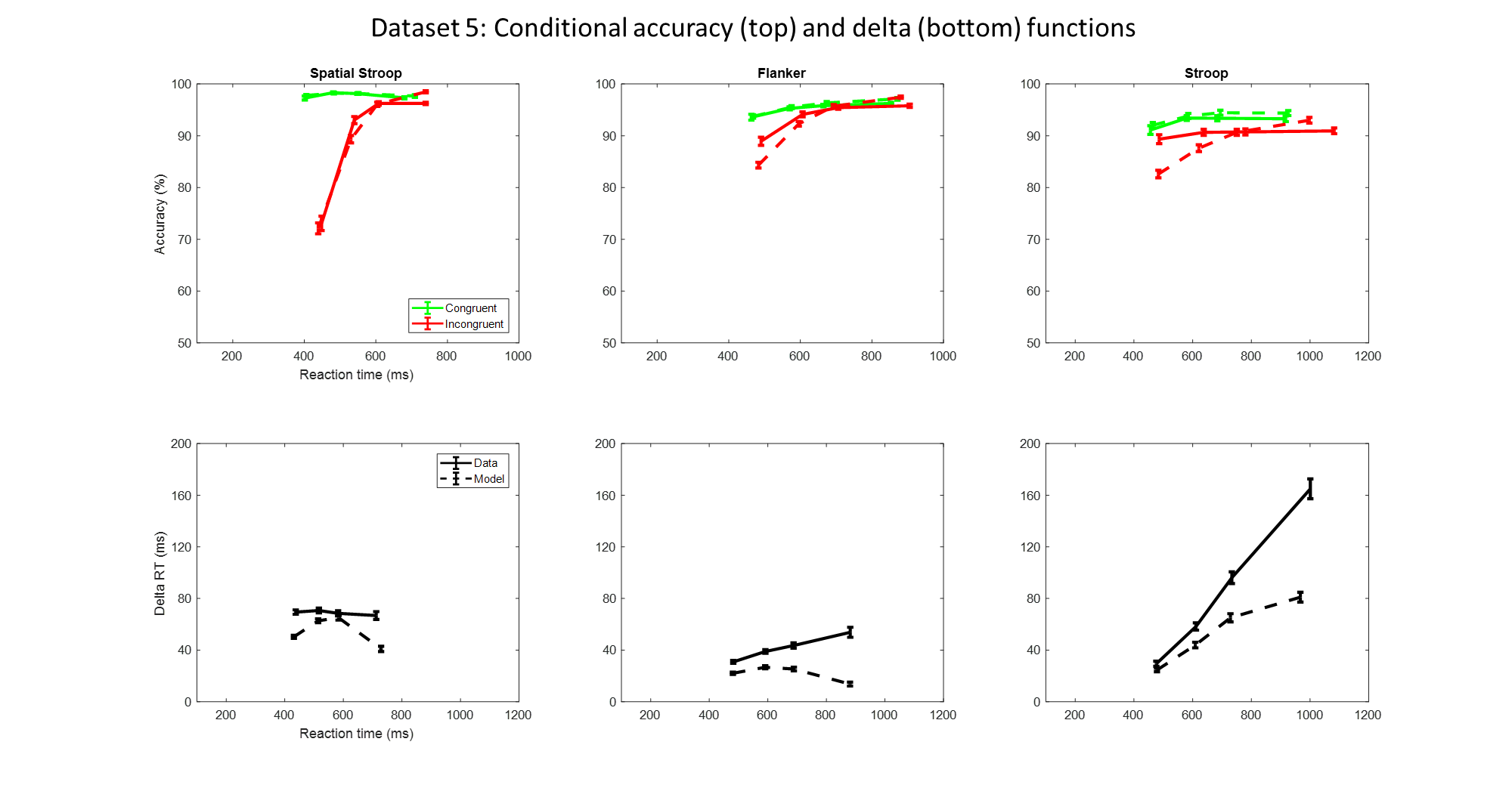


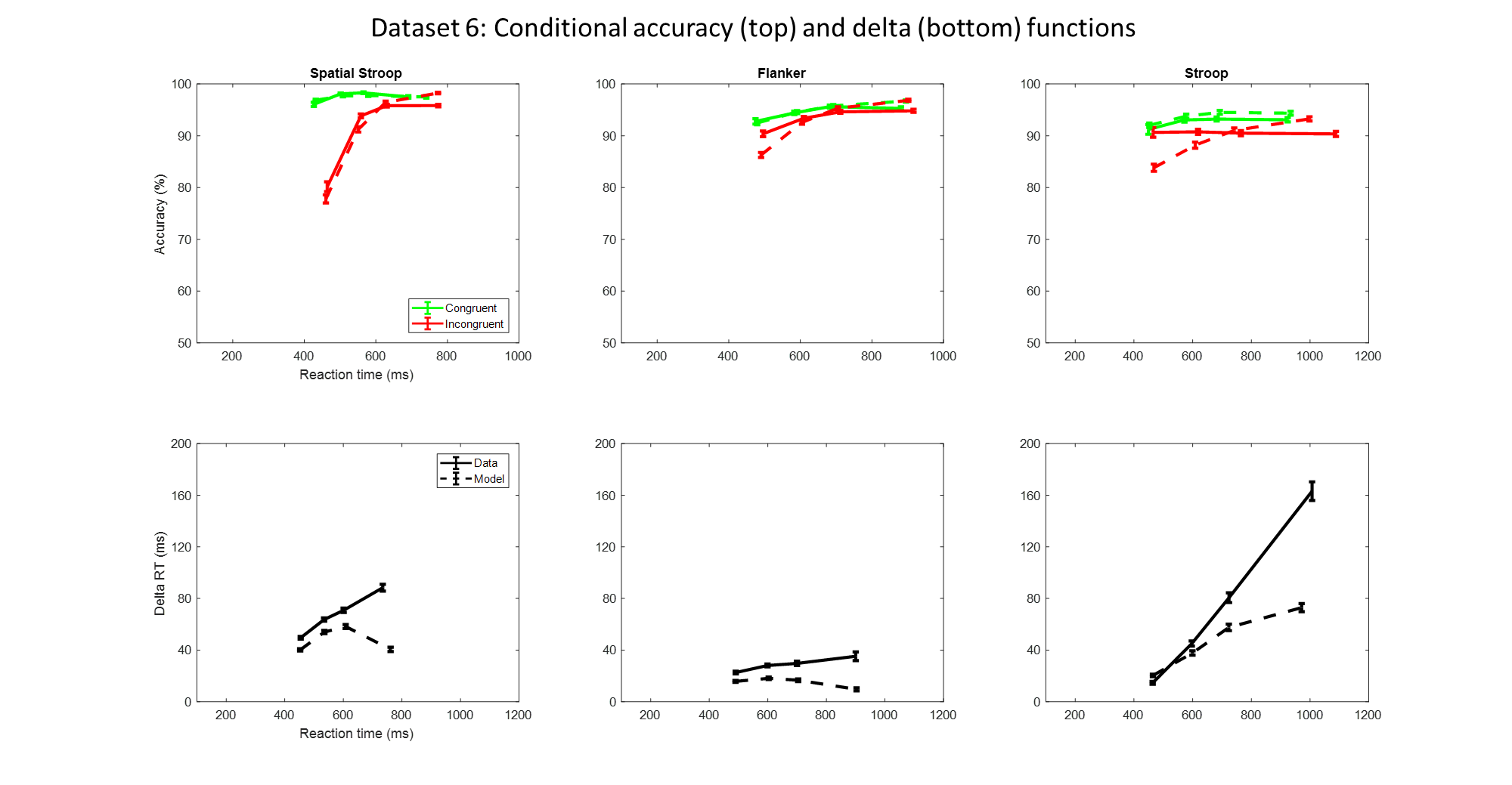


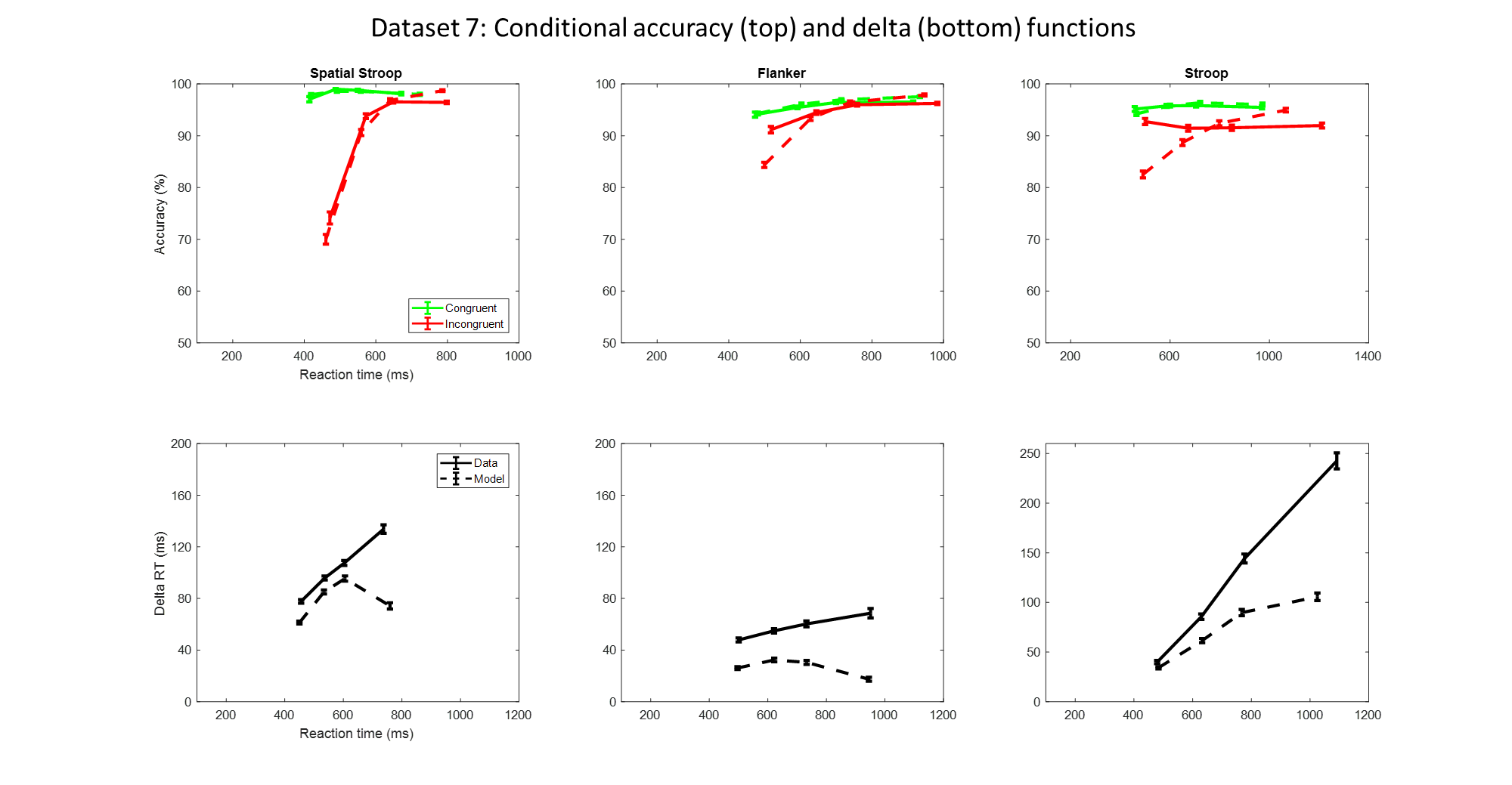

Supplement: Supplementary file 1 [file xlm0001028.docx]
